# Small-Molecule Inhibitors Targeting Lipolysis in Human Adipocytes

**DOI:** 10.1021/jacs.1c10836

**Published:** 2022-04-01

**Authors:** Gernot F. Grabner, Nikolaus Guttenberger, Nicole Mayer, Anna K. Migglautsch-Sulzer, Christian Lembacher-Fadum, Nermeen Fawzy, Dominik Bulfon, Peter Hofer, Thomas Züllig, Lennart Hartig, Natalia Kulminskaya, Gabriel Chalhoub, Margarita Schratter, Franz P. W. Radner, Karina Preiss-Landl, Sarah Masser, Achim Lass, Rudolf Zechner, Karl Gruber, Monika Oberer, Rolf Breinbauer, Robert Zimmermann

**Affiliations:** † Institute of Molecular Biosciences, University of Graz, Heinrichstrasse 31/2, 8010 Graz, Austria; ‡ Institute of Organic Chemistry, Graz University of Technology, Stremayrgasse 9, 8010 Graz, Austria; § BioTechMed-Graz, Mozartgasse 12/2, 8010 Graz, Austria; ∥ BioHealth Field of Excellence, University of Graz, Universitätsplatz 3, 8010 Graz, Austria

## Abstract

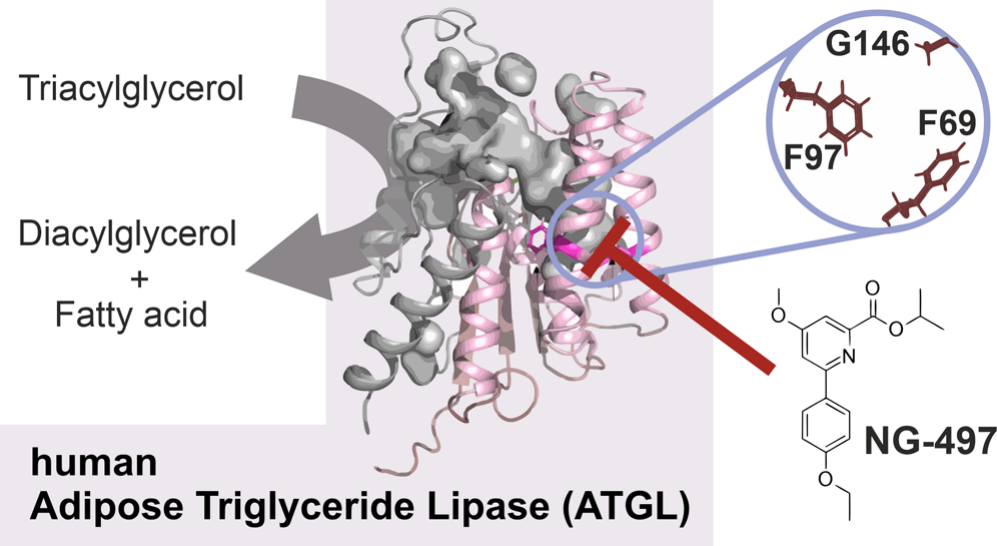

Chronically elevated circulating fatty acid levels promote lipid accumulation in nonadipose tissues and cause lipotoxicity. Adipose triglyceride lipase (ATGL) critically determines the release of fatty acids from white adipose tissue, and accumulating evidence suggests that inactivation of ATGL has beneficial effects on lipotoxicity-driven disorders including insulin resistance, steatohepatitis, and heart disease, classifying ATGL as a promising drug target. Here, we report on the development and biological characterization of the first small-molecule inhibitor of human ATGL. This inhibitor, designated NG-497, selectively inactivates human and nonhuman primate ATGL but not structurally and functionally related lipid hydrolases. We demonstrate that NG-497 abolishes lipolysis in human adipocytes in a dose-dependent and reversible manner. The combined analysis of mouse- and human-selective inhibitors, chimeric ATGL proteins, and homology models revealed detailed insights into enzyme–inhibitor interactions. NG-497 binds ATGL within a hydrophobic cavity near the active site. Therein, three amino acid residues determine inhibitor efficacy and species selectivity and thus provide the molecular scaffold for selective inhibition.

## Introduction

In mammals and many other vertebrates, energy is mainly stored as triacylglycerol (TAG) in white adipose tissue (WAT). Upon demand, fatty acids (FAs) are mobilized from TAG stores in a process termed intracellular lipolysis.^1^ FAs are then utilized as energy substrates, signaling molecules, or building blocks for complex lipids. TAG synthesis and lipolysis in WAT are tightly regulated processes ensuring an adequate FA supply according to the body’s demand.^2^ The dysregulation of these processes can result in elevated circulating FA concentrations leading to ectopic TAG accumulation in nonadipose tissues, which is associated with insulin resistance, local inflammation, and impaired tissue function.^3−5^ This pathological condition is commonly designated as lipotoxicity, frequently observed in obesity, and represents a serious health risk factor leading to life-threatening diseases such as type 2 diabetes and metabolic syndrome.^6^ Ectopic TAG accumulation per se is not the driving force promoting lipotoxicity, but rather reflects chronic oversupply with unesterified FAs promoting the formation of lipotoxic metabolites.^7^ In particular, the accumulation of diacylglycerols and ceramides in nonadipose tissues has been mechanistically linked with insulin resistance and inflammation.^8^

The lipotoxicity concept implicates that inhibition of lipolysis in WAT represents an attractive pharmacological strategy preventing the increased flux of FAs to nonadipose tissue and the concomitant accumulation of lipotoxic metabolites.^9^ Lipolysis in adipocytes primarily depends on two lipases, adipose triglyceride lipase (ATGL) and hormone-sensitive lipase (HSL).^10^ ATGL initiates TAG degradation by hydrolyzing the first ester bond generating diacylglycerols (DAG) and FAs.^11^ HSL subsequently converts DAGs into monoacylglycerol and FAs.^12^ The final step leading to the mobilization of glycerol is catalyzed by HSL and monoacylglycerol lipase (MGL).^13^ Accumulating evidence suggests that inactivation of lipolysis in WAT by genetic approaches reduces plasma FA concentrations and protects from ectopic lipid accumulation and co-morbidities.^14−16^ Similarly, the mouse-selective ATGL inhibitor Atglistatin (Ai) protects from high-fat diet-induced insulin resistance, liver steatosis, and liver inflammation.^17^ The improvement of liver pathology is of particular clinical importance as hepatic steatosis affects approximately 25% of the global population and can progress to steatohepatitis, cirrhosis, and hepatocellular carcinoma.^18^

In addition to obesity-driven metabolic diseases, ATGL inhibition could have beneficial effects in disorders associated with an uncontrolled loss of adipose tissues, such as lipodystrophy and cachexia. Lipodystrophy is caused by a group of genetic or acquired disorders and is characterized by defective production and maintenance of fat in WAT leading to severe ectopic lipid deposition.^19^ Cachexia is commonly observed in cancer and chronic pulmonary, cardiac, and renal disease. It is characterized by ongoing muscle and adipose tissue loss that cannot be entirely reversed with nutritional supplementation.^20^ Studies in mice suggest that inhibition of ATGL can counteract the unwanted loss of adipose tissues in cancer cachexia^21^ and Berardinelli–Seip congenital lipodystrophy (BSCL2).^22^ A very recent study investigated the role of ATGL in the recovery from severe burn injury in mice, which causes hypermetabolism, WAT loss, and ectopic lipid accumulation.^23^ These metabolic alterations are a hallmark of severe burn contributing to poor outcomes in humans.^24^ Notably, adipose tissue-specific deletion of ATGL and Atglistatin treatment reduced circulating FAs, ectopic lipid deposition, WAT browning, and liver dysfunction.^23^ The authors suggested that inhibition of ATGL in burn-injured hypermetabolic patients would improve outcomes.

Strikingly, several studies within recent years demonstrated that ATGL inactivation ameliorates heart failure in mice induced by pressure overload or chronic adrenergic stimulation.^25−29^ Protective effects were observed in adipose-specific ATGL-knockout and Atglistatin-treated mice, suggesting an important role of WAT lipolysis in the pathogenesis of heart disease. This observation could be of utmost importance, as heart failure is the leading cause of death worldwide and new therapeutic concepts are urgently needed.^30^

Despite the importance of ATGL in clinically highly relevant diseases, selective inhibitors of human ATGL are currently not available. Here, we report on the development and the biological characterization of the first small-molecule inhibitor of human ATGL termed **NG-497**. We demonstrate that **NG-497** is highly selective, nontoxic, and diminishes FA release from human adipocytes in a reversible manner. Using chimeric proteins and homology modeling, we identified a hydrophobic cavity near the active site of ATGL, in which three amino acids (aa) determine the efficacy and species selectivity of the inhibitor.

## Results

### Atglistatin Acts as Species-Selective ATGL Inhibitor

**Figure 1:**
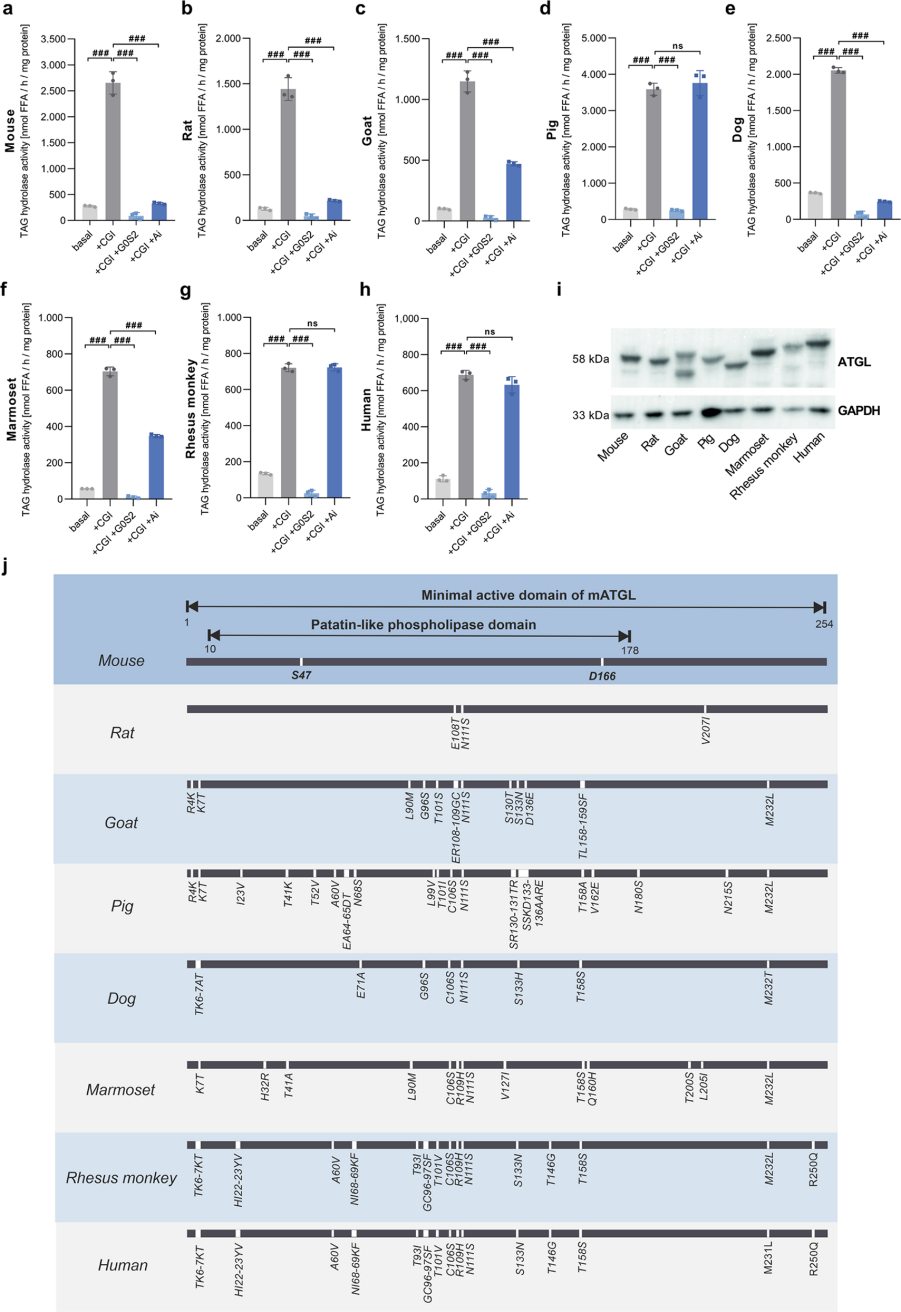
Atglistatin exhibits species selectivity. (a) TAG hydrolase activity detected in lysates of Expi293 cells expressing mouse, (b) rat, (c) goat, (d) pig, (e) dog, (f) marmoset, (g) rhesus monkey, and (h) human ATGL. Activity was determined in the absence (basal) or presence of purified mouse CGI-58, purified mouse G0S2, and Atglistatin (Ai) (40 μM). Data are shown as mean ± standard deviation (SD). Statistical significance was determined via analysis of variance (ANOVA) followed by Bonferroni *post hoc* test (^#^*p* < 0.05; ^##^*p* < 0.01; ^###^*p* < 0.001). (i) Protein expression of ATGL orthologues was confirmed by Western blotting analysis with GAPDH as loading control. (j) Sequence alignment of the minimal active domain of ATGL orthologues. Amino acid residues, which are not identical to mouse ATGL, are indicated for each species. Active site amino acids S47 and D166 are shown for mouse ATGL.

We previously developed Atglistatin (Ai), a selective inhibitor of mouse ATGL,^31,32^ which is ineffective against human ATGL.^17^ Ai acts as a competitive inhibitor targeting the minimal active domain of ATGL ranging from aa 1–254.^33^ This domain is highly conserved in different mammalian species (∼90% identity, Table 1) and comprises the patatin-like domain, eponymous for all members of the patatin-like phospholipase domain-containing (PNPLA) protein family.^34^ We first investigated the species selectivity of Ai by performing TAG hydrolase assays^35^ with lysates from Expi293 cells expressing ATGL orthologues of different species. The activity of ATGL is strongly stimulated by the co-activator protein comparative gene identification-58 (CGI-58, also known as α/β hydrolase domain containing 5, ABHD5)^36^ and can be suppressed by the inhibitory protein G0/G1 switch 2 (G0S2).^37^ We therefore determined ATGL activity in the presence and absence of mouse CGI-58, mouse G0S2, and Ai. As shown in Figure 1a–i, all ATGL orthologues were expressed, enzymatically active, stimulated by mouse CGI-58, and inactivated by mouse G0S2. Ai inhibited mouse (Figure 1a), rat (Figure 1b), goat (Figure 1c), dog (Figure 1e), and marmoset ATGL (Figure 1f), but not pig (Figure 1d), rhesus monkey (Figure 1g), or human ATGL (Figure 1h). These results suggest that small variations within the highly conserved minimal active domain of ATGL strongly affect inhibitor binding (Figure 1j).

**Table 1:** Protein Sequence Identity of Mouse ATGL and Species Orthologues

		identity (% of mouse)		
species	reference	aa 10–178	aa 1–254	whole protein
rat	NP_001101979.2	98.8	98.8	96.7
goat	NP_001272668.1	93.5	94.5	89.3
pig	XP_020932192.1	88.8	90.6	80.2
dog	XP_025307704.1	96.5	96.5	89.6
marmoset	XP_009006999.1	94.7	94.9	89.9
rhesus monkey	XP_014968767.1	91.1	92.5	86.0
human	NP_065109.1	91.1	92.5	88.6

### Development of NG-497, a Selective Inhibitor of Human ATGL

**Figure 2:**
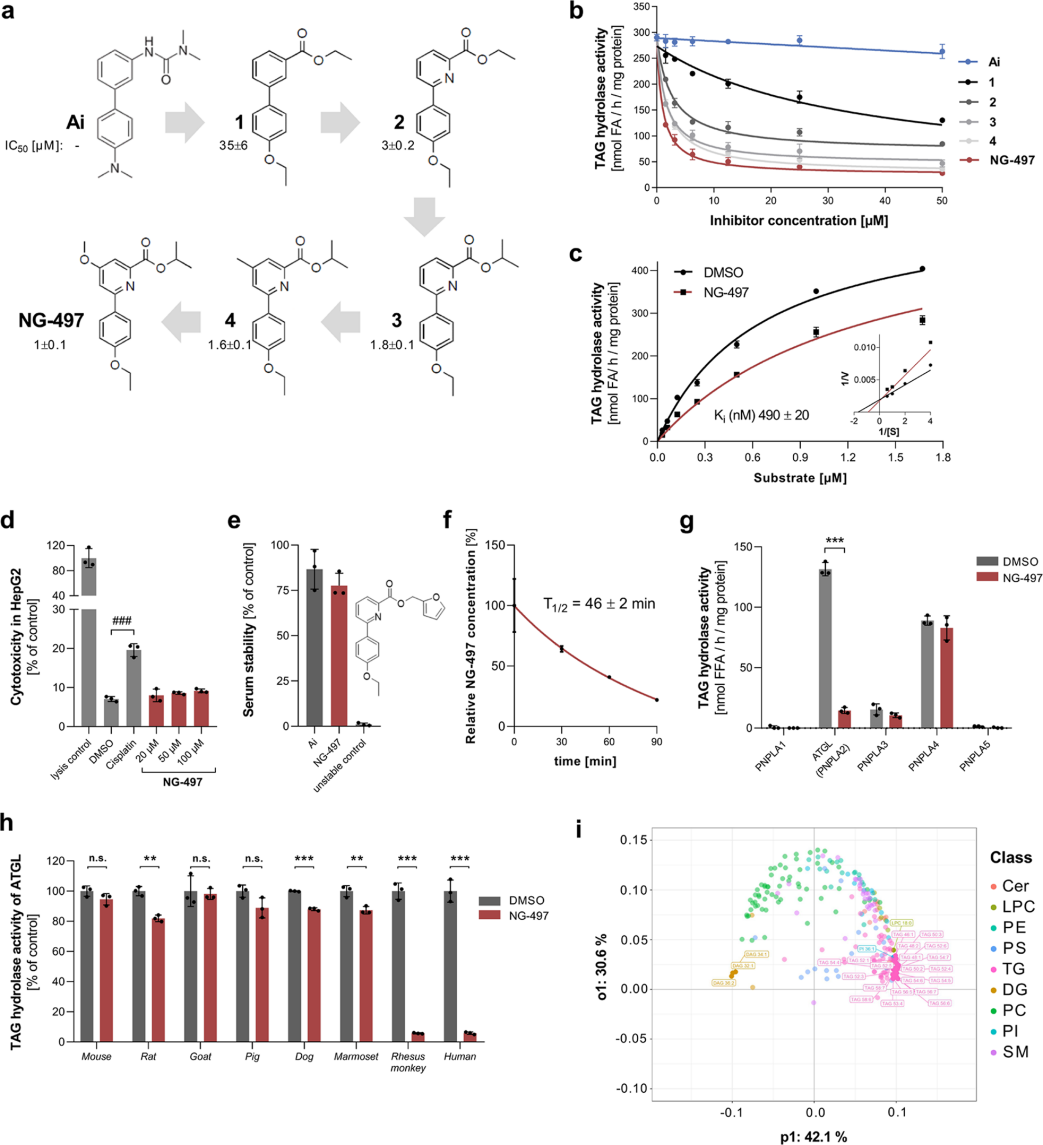
Development and characterization of the human ATGL inhibitor **NG-497**. (a) Structure and IC_50_ values of human ATGL inhibitors. (b) Dose-dependent inhibition of human ATGL by respective inhibitors. (c) ATGL activity was determined at the indicated substrate concentrations in the absence and presence of **NG-497** (0.5 μM). *K*_i_ was determined by nonlinear regression analysis using GraphPad Prism. Lineweaver–Burk analysis of ATGL inhibition is shown in the inset. (d) Toxicity of **NG-497** in HepG2 cells was determined after 24 h incubation at indicated concentrations. Cisplatin and DMSO were used as positive and negative controls, respectively. Cytotoxicity was determined using the Roche LDH Kit and calculated as relative LDH release compared to fully lysed cells. (e) Stability of **NG-497** was analyzed via ultrahigh-performance liquid chromatography–mass spectrometry (UHPLC-MS) after incubation in human serum for 0 or 3 h at 37 °C. Atglistatin and a chemically related unstable compound were used as controls. Data are presented as % decrease of initial inhibitor concentrations. (f) Stability of **NG-497** exposed to HepG2 cells. Cells were treated for 1 h with 40 μM NG-497. Subsequently, the inhibitor was removed and cells were incubated in Dulbecco’s modified Eagle’s medium (DMEM) 10% FCS. **NG-497** concentrations/well were determined at the indicated time points using UHPLC-MS. (g) TAG hydrolase activity of PNPLA1–5 in the absence and presence of **NG-497** (100μM). (h) Inhibition of ATGL orthologs from different species by **NG-497** (50 μM). Data are presented as mean ± SD. Statistical significance was determined via *t* test (*p < 0.05; **p < 0.01; ***p < 0.001) or ANOVA followed by Bonferroni *post hoc* test (^###^*p* < 0.001). (i) Lipidomic changes in NG-497-treated HepG2 cells. The cells were incubated with 40 μM inhibitor or DMSO as control for 3 h (*n* = 5). Subsequently, lipids were extracted and subjected to lipidomic analysis using UHPLC-MS. The detected 297 lipid species of both groups were analyzed with supervised multivariate analysis OPLS-DA and are shown as a loading plot. Lipids are colored by class, and the top 25 contributing lipid species are highlighted.

To develop an inhibitor for human ATGL, we screened compounds synthesized in the course of the development of Ai.^31^ Screenings were performed in the presence of purified CGI-58 using cell lysates of Expi293 cells overexpressing ATGL as a source of enzymatic activity and radiolabeled triolein as substrate as described above (Figure 1). These screening assays identified the biphenylester compound **1** as a suitable starting point (IC_50_ = 35 μM). In an iterative optimization process, which encompassed the synthesis and testing of >150 compounds (not shown), we varied the nature of the top aryl ring (Figure 2a). Replacement of the top phenyl group with a pyridine moiety (compound **2**) led to a significant improvement in inhibitor efficacy (IC_50_ = 3 μM), which we interpret as a result from a smaller angle between the 1,3-arrangement of the aryl- and the ester-substituents due to the shorter C–N bond length within the pyridine group. Further variations in the ester groups led to isopropylester **3** with improved efficacy (IC_50_ = 1.8 μM). While the variation of the bottom ring did not lead to any further improvement (not shown), introducing additional substituents in the 4-position of the pyridine ring moderately improved efficacy (compound **4**). The introduction of a methoxy residue at this position finally led to compound **NG-497** showing an IC_50_ value of 1 μM. Dose-dependent inhibition of TAG hydrolase activity of human ATGL by different inhibitors is shown in Figure 2b.

To investigate the mechanism of action, we performed kinetic analysis by varying substrate concentration in the absence and presence of **NG-497**. Using nonlinear regression analysis, we determined a *K*_i_ value of 0.5 μM (Figure 2c). Lineweaver–Burk analysis revealed that **NG-497** causes an increase of the apparent *K*_m_ value, but does not affect *V*_max_ indicating reversible and competitive inhibition (Figure 2c, inset). Next, we investigated whether **NG-497** affects the activation of ATGL by CGI-58 and its inhibition by G0S2. Partially purified ATGL was inactivated by **NG-497** with similar IC_50_ values in the presence and absence of CGI-58 suggesting that the inhibitor directly targets ATGL (Figure S1). To investigate whether **NG-497** compromises the protein–protein interaction of ATGL and G0S2, we performed co-immunoprecipitation experiments revealing that **NG-497** does not block the interaction. In contrast, we observed increased G0S2 binding (Figure S2) indicating that G0S2 and **NG-497** could synergistically inhibit ATGL in cells expressing G0S2.

To further characterize **NG-497**, we analyzed its toxicity, biological stability, and selectivity. Cytotoxicity assays revealed that **NG-497** has no toxic effects in HepG2 cells up to a concentration of 100 μM (Figure 2d). Similar to Ai, **NG-497** was not metabolized in human serum, while a control compound featuring a furfuryl ester was completely degraded after 3 h (Figure 2e). Conversely, **NG-497** showed a half-life of 46 min when exposed to HepG2 cells (Figure 2f). These observations indicate that the inhibitor shows high stability in serum but is degraded or modified after cellular internalization.

To investigate the selectivity of **NG-497**, we first tested whether it inactivates other TAG hydrolases of the PNPLA family. Besides ATGL (also referred to as PNPLA2), humans express 8 other PNPLAs, of which PNPLA1-5 exhibit the most similar domain architecture and the highest sequence similarity.^34^ We detected TAG hydrolase activity for human PNPLA2–4 but not for PNPLA1 and 5 and **NG-497** inactivated the TAG hydrolase activity of ATGL (PNPLA2) but not that of PNPLA3 and 4 (Figure 2g). PNPLA1 catalyzes the synthesis of ω-*O*-acylceramides,^38^ which was not affected by **NG-497** (Figure S3a). Furthermore, we did not observe inhibition of the more distantly related phospholipases PNPLA6, PNPLA7, PNPLA8, and PNPLA9^39^ (Figure S3b–e), of the human acylglycerol hydrolases DDHD domain-containing protein 2 (DDHD2),^40^ HSL,^41^ and carboxylesterase 2 (CES2)^42^ (Figure S3f–h), as well as pancreatic lipase (Figure S3i), the major TAG lipase of the intestine.^43^ Finally, we did not observe inhibition of heparin-releasable TAG hydrolase activity in human serum (Figure S3j) suggesting that **NG-497** does not affect the major circulating TAG hydrolases lipoprotein lipase (LPL) and hepatic lipase (HL).^44^ Next, we tested the species selectivity of **NG-497** and found that it inactivates ATGL from humans and rhesus monkeys. The inhibitor had no substantial effects on ATGL orthologues from mouse, rat, goat, pig, dog, and marmoset with less than 20% reduction in TAG hydrolase activity at a concentration of 50 μM (Figure 2h). In summary, our observations show that **NG-497** acts as a selective, nontoxic inhibitor of human and rhesus monkey ATGL.

To further address the effects of **NG-497** on cellular lipid metabolism, we performed untargeted lipidomic analysis of HepG2 cells treated with **NG-497** or DMSO as control. Using UHPLC-MS, we detected 219 lipid species and analyzed inhibitor-mediated changes using the supervised multivariate model “orthogonal partial least squares discriminant analysis” (OPLS-DA, Figure 2i; the score plot is shown in Figure S4). This model can be used to separate lipid species according to their contribution to lipidomic changes.^45^ 20 of the top 25 hits were due to an increase of TAG species, while three DAG species were decreased (highlighted in Figure 2i). Alterations in lipid species were also analyzed with univariate statistics and are shown in Table S1. In addition to acylglycerol species, we observed moderate changes in ceramide and glycerophospholipid species. Overall, however, the observed lipidomic changes are highly indicative of the lack of TAG hydrolase activity in **NG-497**-treated cells.

### NG-497 Inhibits Lipolysis in Human Adipocytes

**Figure 3:**
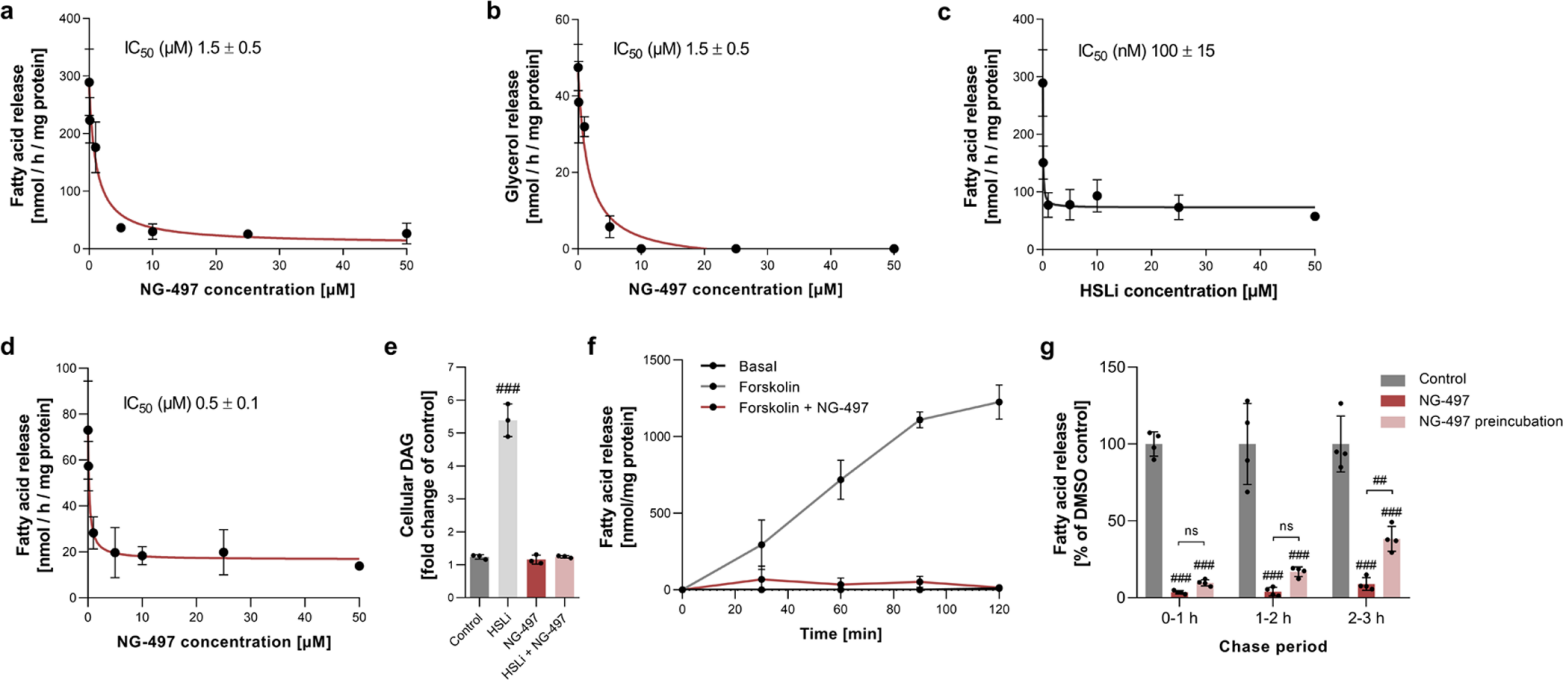
**NG-497** inhibits lipolysis in human SGBS and primary human adipocytes. SGBS adipocytes were preincubated with **NG-497** for 1 h at the indicated concentrations. Subsequently, lipolysis was stimulated with isoproterenol (1 μM) and (a) FA and (b) glycerol release was determined after 1 h using commercial kits. (c) Dose-dependent inhibition of isoproterenol-stimulated FA release from SGBS adipocytes by the HSL inhibitor 76-068 (HSLi). (d) Combined inhibition of HSL and ATGL in SGBS adipocytes using HSLi (25 μM) and the indicated concentrations of **NG-497**. (e) DAG accumulation in SGBS adipocytes was analyzed by thin-layer chromatography (TLC) following 1 h incubation with HSLi (25 μM), **NG-497** (40 μM), and a combination of both inhibitors. (f) Primary human adipocytes were prelabeled with 1 μCi [9,10-^3^H] oleic acid per well for 12 h. Subsequently, basal and Forskolin (5 μM)-stimulated release of radioactivity was determined via liquid scintillation over a period of 2 h in the presence or absence of **NG-497** (40 μM). FA release was calculated based on FA concentrations in conditioned media after 2 h as determined using a commercial kit. (g) Primary human adipocytes were preincubated in the presence or absence of **NG-497** (40 μM) for 1 h. Subsequently, the medium was changed and FA release was monitored over a period of 3 h under the indicated conditions. Data are presented as mean ± SD. Statistical significance was determined via ANOVA followed by Bonferroni *post hoc* test (^#^*p* < 0.05; ^##^*p* < 0.01; ^###^*p* < 0.001).

We next investigated the effect of **NG-497** on lipolysis in human Simpson–Golabi–Behmel syndrome (SGBS) adipocytes to assess its cellular efficacy.^46^ We observed a dose-dependent decrease of isoproterenol-stimulated FA (Figure 3a) and glycerol release (Figure 3b) with IC_50_ values of 1.5 μM. **NG-497** almost completely abolished lipolysis at concentrations ≥10 μM. In comparison, pharmacological inhibition of HSL using Hi 76-0079 (HSLi)^10^ decreased FA release by maximal 70% with an IC_50_ of 100 nM (Figure 3c). The remaining HSL-independent FA release was inhibited by **NG-497** with an IC_50_ of 0.5 μM (Figure 3d). Analysis of cellular lipids revealed that inhibition of HSL but not ATGL increased DAG levels 5-fold, while combined inhibition of HSL and ATGL prevented DAG accumulation (Figure 3e). These results confirm that the lipolytic cascade, in which ATGL performs the initial step of lipolysis generating DAG and HSL is rate-limiting for subsequent hydrolysis of DAG,^10^ is conserved from mice to humans.

We further investigated the cellular efficacy of **NG-497** in primary human adipocytes, which more closely resemble the metabolic characteristics of human WAT. We therefore labeled cellular lipids with [9,10-^3^H] oleic acid and analyzed time-dependent basal and Forskolin-stimulated release of radiolabeled FAs. While the basal release was barely detectable, Forskolin induced a pronounced increase of FA release over 2 h, and **NG-497** diminished Forskolin-induced FA release to basal level (Figure 3f). Similar results were obtained using primary human omental adipocytes, where NG-497 treatment extinguished Forskolin-induced TAG degradation and FA release (Figure S5a,b).

Next, we investigated the reversibility of ATGL inhibition in primary adipocytes. We therefore preincubated adipocytes with **NG-497** for 1 h and subsequently analyzed FA release in medium with and without inhibitor for 3 h. Inhibitor pretreatment caused a substantial reduction in FA release over the whole chase period (Figure 3g). Yet, pretreated cells regained ∼40% of their lipolytic activity within the third hour after pretreatment, indicating reversible inhibition of ATGL due to metabolization of the inhibitor.

### NG-497 Affects Lipolysis-Dependent Respiration in HepG2 Cells and Does Not Induce Mitochondrial Dysfunction

**Figure 4:**
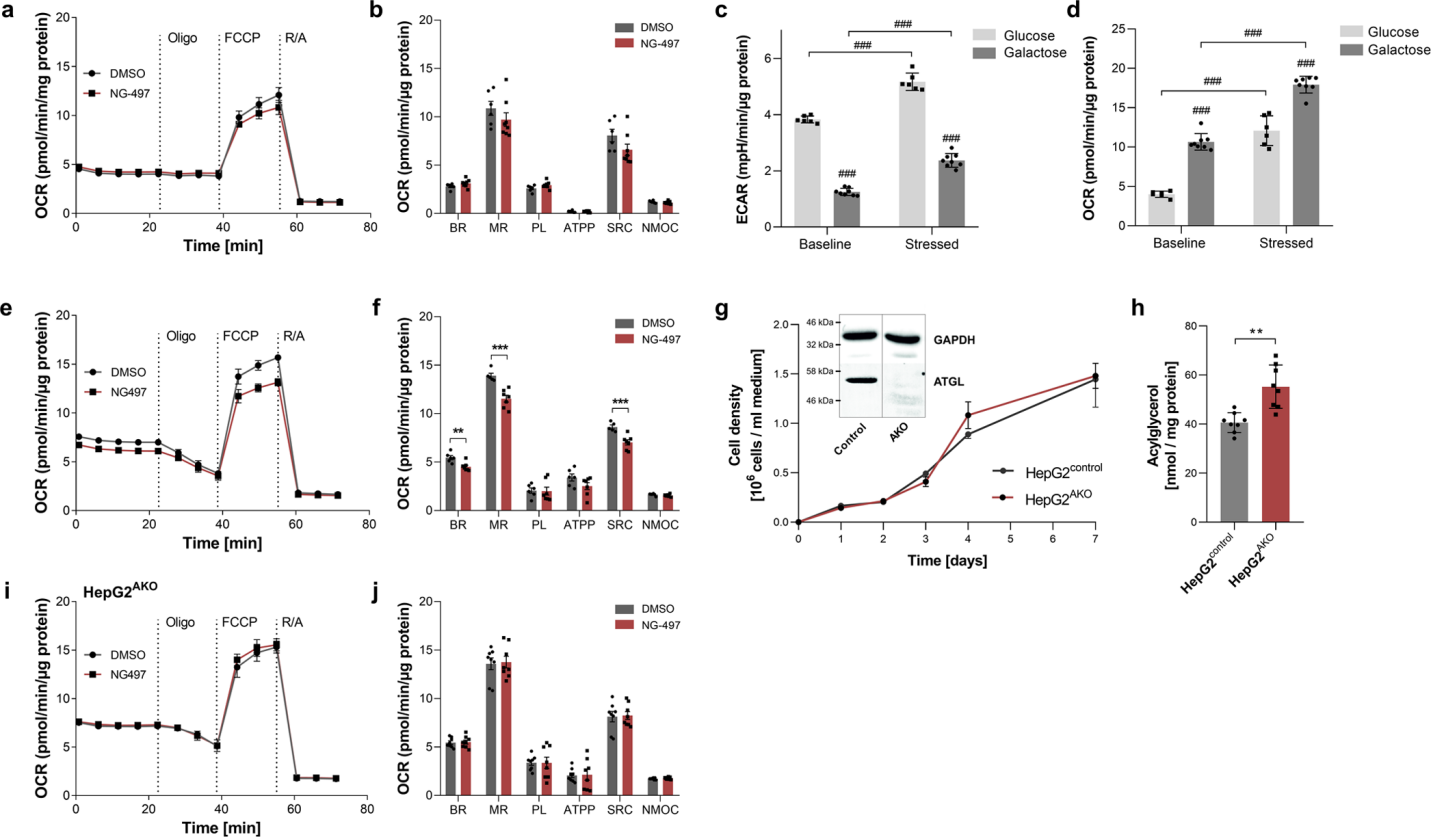
**NG-497** reduces lipolysis-dependent respiration of HepG2 cells and does not compromise mitochondrial function via off-target effects. (a) HepG2 cells were treated with **NG-497** (40 μM) or DMSO as control and oxygen consumption rate (OCR) was determined in Seahorse growth medium supplemented with glucose (25 mM) using the SeaHorse XF96 extracellular flux analyzer. OCR was determined after subsequent addition of 1 μM Oligomycin (ATP-synthase inhibitor), 1 μM FCCP (uncoupler), and 1 μM antimycin/rotenone (electron transport chain inhibitors). (b) Statistical analysis of basal respiration (BR), maximal respiration (MR), proton leak (PL), ATP production (ATPP), spare respiratory capacity (SRC), and nonmitochondrial oxygen consumption (NMOC) of control and **NG-497**-treated cells. (c) Extracellular acidification rate (ECAR) and (d) OCR of HepG2 cells were determined in Seahorse growth medium supplemented with glucose (25 mM) or galactose (10 mM). (e) Determination of the ORC of HepG2 cells grown in medium supplemented with galactose (10 mM) and NG-497 (40 μM) or DMSO as control with (f) respective statistical analysis. (g) *ATGL* gene was inactivated in HepG2 cells using the CRISPR/Cas9 system. Growth of cells expressing (HepG2^control^) or lacking functional ATGL (HepG2^AKO^) was determined using a BioRad cell analyzer. ATGL expression was analyzed by Western blotting analysis using an anti-ATGL antibody and GAPDH as a loading control (inset). (h) Acylglycerol content of HepG2^control^ and HepG2^AKO^ cells was determined by a commercial kit (TG Infinity reagent, Thermo Fisher Scientific). (i) OCR of galactose-supplemented HepG2^AKO^ cells and (j) the respective statistical analysis. Data are presented as mean ± standard error of the mean (SEM). Statistical significance was determined via Student’s *t* test (*p < 0.05; **p < 0.01; ***p < 0.001) or ANOVA followed by Bonferroni *post hoc* test (^#^*p* < 0.05; ^##^*p* < 0.01; ^###^*p* < 0.001).

**NG-497** could affect respiration by limiting the availability of FAs as an energy substrate or by off-target effects leading to mitochondrial dysfunction, which is a major mechanism of drug-induced toxicity.^47^ To monitor on- and off-target effects of **NG-497** on cellular respiration, we performed seahorse experiments using HepG2 cells endogenously expressing (HepG2^control^) or lacking ATGL (HepG2^AKO^). This method allows the measurement of basal respiration (BR), maximal respiration (MR), proton leak (PL), ATP production (ATPP), spare respiratory capacity (SRC), and nonmitochondrial oxygen consumption (NMOC) after successive addition of oligomycin (ATP-synthase inhibitor), carbonyl cyanide *p*-trifluoromethoxyphenylhydrazone (FCCP) (uncoupler), and antimycin/rotenone (electron transport chain inhibitors). The human hepatoma cell line HepG2 was selected as a model since it is frequently used to monitor drug-induced mitochondrial dysfunction.^48^ Like most cancer cell lines, HepG2 cells rely on anaerobic glycolysis for ATP generation, implicating that inhibition of lipolysis has only minor effects on respiration. Accordingly, we could not observe significant effects of **NG-497** on HepG2^control^ cells, when the culture medium was supplemented with glucose (Figure 4a,b). Yet, the cells showed a tendency for reduced MR (−11%) and SRC (−18%).

Replacement of glucose by galactose as an oxidizable substrate in the culture medium has been demonstrated to cause a metabolic shift from glycolysis to oxidative phosphorylation.^49^ Under these conditions, oxidation of FAs mobilized from TAG stores can significantly contribute to respiration. We first verified that galactose treatment leads to a reduced extracellular acidification rate (ECAR), which is a measure for the contribution of glycolysis to ATP production. The switch from glucose to galactose medium caused a 67 and 55% reduction in baseline and stressed (FCCP-induced) ECAR (Figure 4c). Consistently, galactose treatment increased basal and stressed OCR 2.6- and 1.5-fold, respectively (Figure 4d). In the presence of galactose, **NG-497** significantly reduced basal OCR (BR, −15%), maximal respiration (MR, −17%), and spare respiratory capacity (SRC, −20%) of HepG2^control^ cells (Figure 4e,f), while all other parameters remained unchanged.

To verify that the observed changes in respiration were caused by on-target effects, the experiments were repeated with HepG2^AKO^ cells, which were generated using the CRISPR/Cas9 system. These cells showed similar growth to control cells (Figure 4g), exhibited complete loss of ATGL protein (Figure 4g, inset), and showed a moderate increase in TAG stores (Figure 4h). **NG-497** had no effect on respiration in HepG2^AKO^ cells cultured in galactose medium (Figure 4i,j), demonstrating that the inhibitor reduced respiration via ATGL inhibition without causing off-target effects on respiration.

### NG-497 Binds within a Cavity Near the Active Site of ATGL

**Figure 5:**
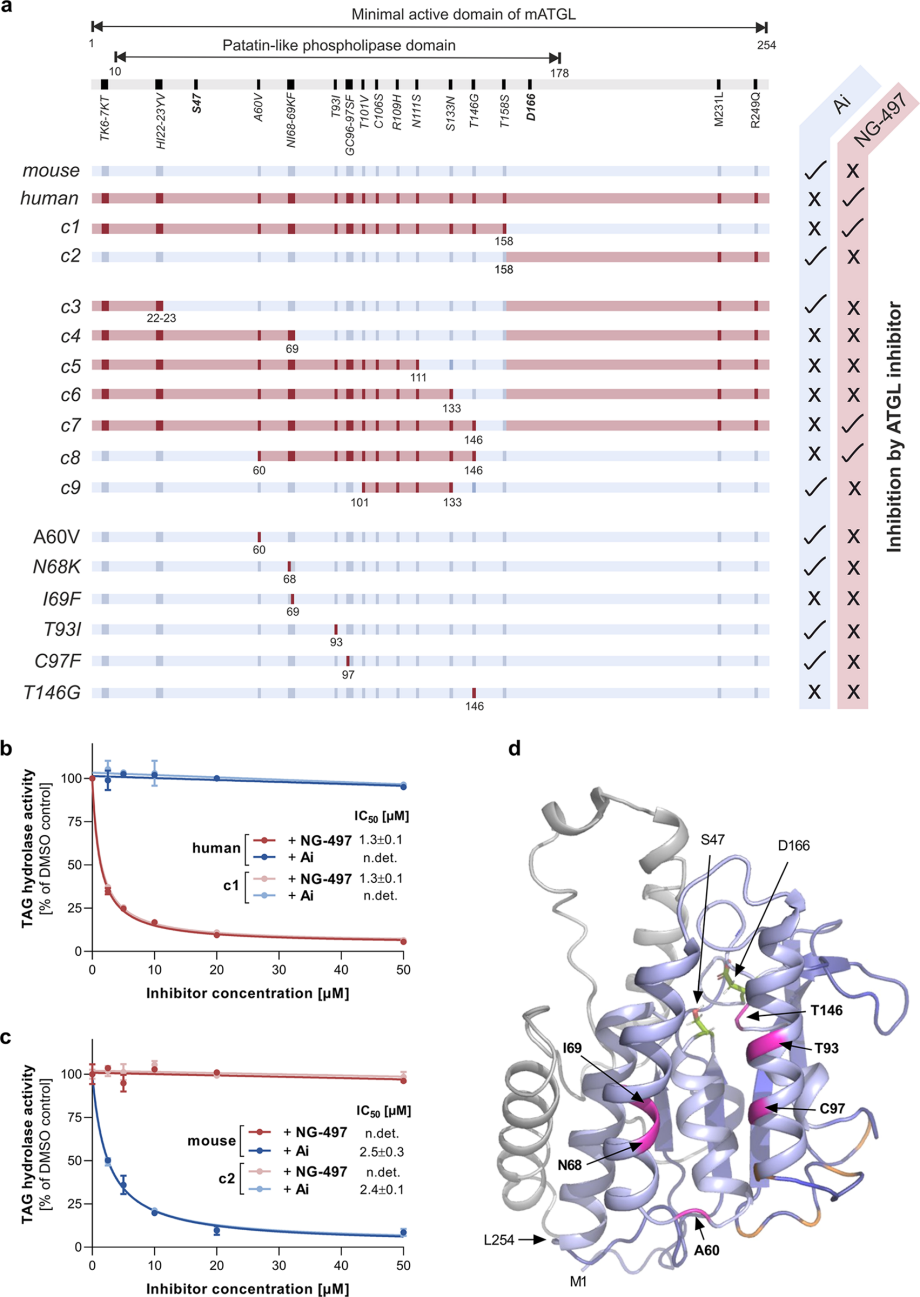
Analysis of chimeric proteins comprising human and mouse ATGL. (a) Schematic view of the minimal active domain of wild-type and chimeric ATGL variants (c1–c9). Nonidentical aa are indicated in blue and red for mouse and human ATGL, respectively. ATGL variants were expressed in Expi293 cells and TAG hydrolase activity was determined in cell lysates in the presence or absence of **NG-497** (10 μM) and Atglistatin (10 μM), respectively. Inhibition of ATGL variants by respective inhibitors (tick mark) and lack of inhibition (cross) is indicated at the right edge. The expression of all ATGL variants was confirmed by Western blotting analysis (Figure S6). (b) Dose-dependent inhibition of human ATGL (aa 1–288) and c1 (human aa 1–158 and mouse aa 159–288) by **NG-497**. (c) Dose-dependent inhibition of mouse ATGL (aa 1–288) and c2 (mouse aa 1–158 and human aa 159–288) by Ai. IC_50_ values are shown in the insets. Data are presented as relative inhibition of respective DMSO controls and are indicated as mean ± SD. (d) 3D homology model of the minimal active domain of mouse ATGL (M1–L254) depicted as a cartoon. Residues 1–178 are in blue, and residues 179–254 are in gray. S47 and D166 comprising the active site are represented as green sticks. T101, C106, R109, N111, and S133 are colored in orange, A60, N68, I69, T93, C97, and T146 are colored in magenta.

Despite the high sequence similarity of mammalian ATGL orthologues, both **NG-497** and Ai exhibit distinct species selectivity (Figures 1 and 2), which is likely caused by species-specific aa sequence variances within inhibitor binding sites. Since experimental three-dimensional (3D) structures for ATGL are currently not available, we utilized chimeric variants of mouse and human ATGL for the identification of binding sites. The constructs c1–c9 (Figure 5a) comprised the N-terminal 289 aa of ATGL, which are sufficient to retain enzymatic activity.^50^ In variants c1 and c2, exchange of the N-terminal 158 aa between human and mouse ATGL switched inhibitor reactivity. **NG-497** inhibited human ATGL and the chimeric variant c1 with identical IC_50_ values of 1.3 μM, while Ai was ineffective (Figure 5b). Conversely, Ai inactivated wild-type mouse ATGL and the chimeric variant c2 with similar efficacy (IC_50_ ∼ 2.5 μM), while **NG-497** was ineffective (Figure 5c). These results demonstrate that aa 1–158 entirely determine species selectivity of ATGL inhibitors.

We next generated variants by further stepwise humanization of c2. Variant c3 containing aa 1–23 of human ATGL was inhibited by Ai but not **NG-497** (Figure 5a). Variant c4 (aa 1–69 of human ATGL), c5 (aa 1–111 of human ATGL), and c6 (aa 1–133 of human ATGL) were neither inhibited by Ai nor by **NG-497**. Finally, variants of mouse ATGL containing aa 1–146 (c7) and 60–146 (c8) of human ATGL were inhibited by **NG-497** but not Ai (Figure 5a). These observations demonstrate that aa 60–146 of human ATGL determine **NG-497** binding.

**Figure 6:**
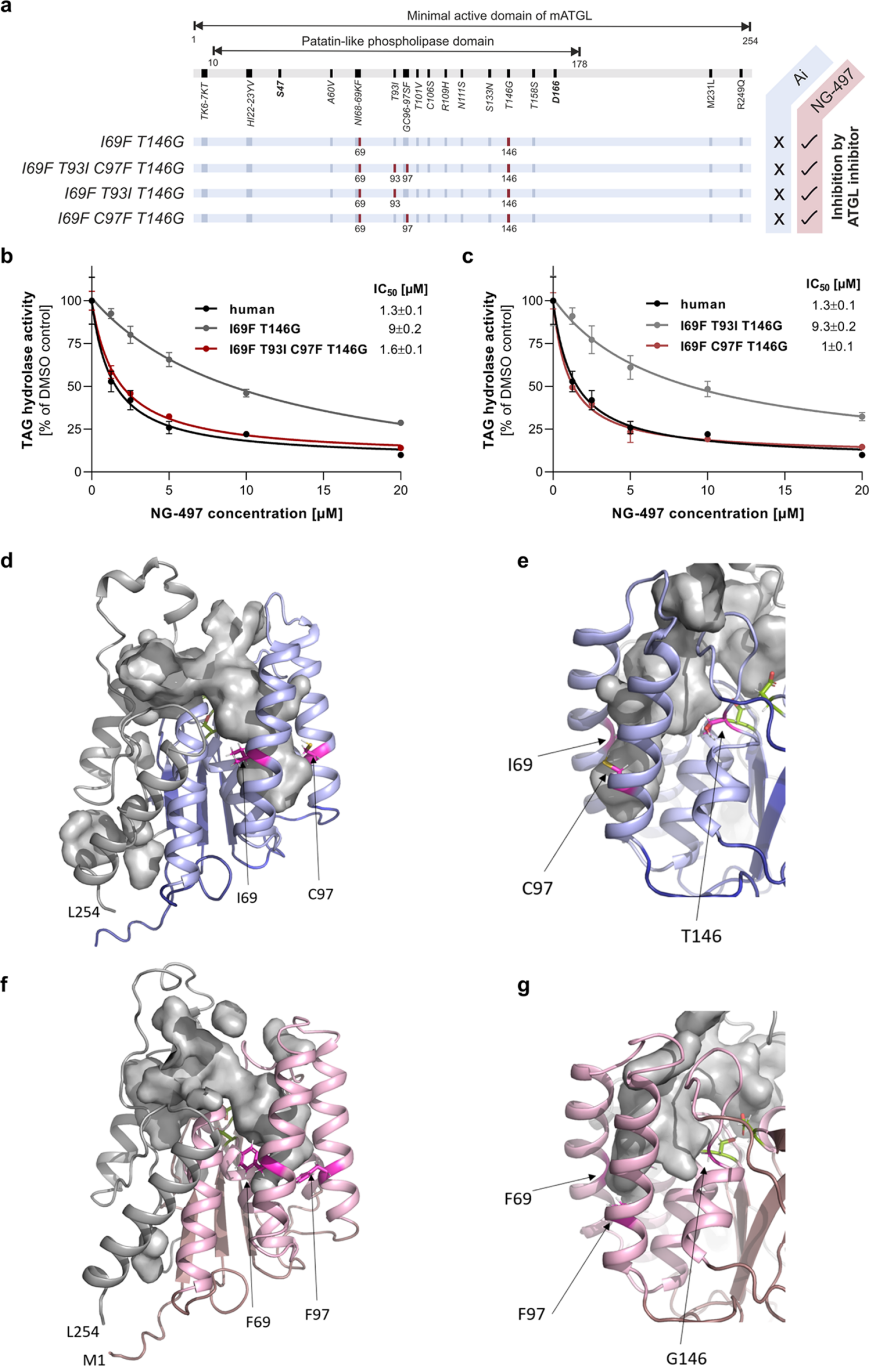
Identification of the molecular scaffold determining species selectivity. (a) Schematic view of mouse ATGL variants, which are sensitive to **NG-497** inhibition. Humanized aa are indicated in red and nonidentical aa are indicated in dark blue. (b) Dose-dependent inhibition of the I69F/T146G double mutant, the I69F/T93I/C97F/T146G quadruple mutant, and human ATGL. (c) Dose-dependent inhibition of triple mutants I69F/T93I/T146G and I69F/C97F/T146G, and human ATGL. IC_50_ values are shown in the insets. Data are presented as relative inhibition of respective DMSO controls and are indicated as mean ± SD of triplicate determinations. The expression of all ATGL variants was confirmed by Western blotting analysis (Figure S6). (d) 3D homology model of mouse ATGL residues M1–L254 depicted as a cartoon. Residues 1–178 are in blue, residues 179–254 are in gray, and S47 and D166 are represented as green sticks. I69, T93, and T146 are shown as magenta sticks and the cavity is shown as gray surface. The deeper right branch of the cavity is confined by I69 and C97 and (e) by T146 on the back side as shown in a close-up view. (f) 3D homology model of human ATGL residues M1–L254 depicted as a cartoon. Residues 1–178 are in pink, residues 179–254 are in gray, and S47 and D166 are represented as green sticks. F69, F97, and T146 are shown as magenta sticks, and the cavity is shown as a gray surface. (g) Close-up view of the homology model of human ATGL after rotation to highlight the position of G146.

The region between aa 60 and 146 of human and mouse ATGL comprises 12 nonidentical aa. We generated 3D homology models for the minimal active domain of mouse ATGL to obtain more detailed insight into the topology of the relevant region (Figure 5d). Based on this model, aa T101, C106, R109, N111, and S133 locate at peripheral (loop) regions (indicated in orange, Figure 5d). Accordingly, humanization of these aa preserved Ai binding, while **NG-497** was ineffective (c9, Figure 5a). For all other nonidentical aa (indicated in magenta, Figure 5d), we generated variants with single aa substitutions. The humanized variants A60V, N68K, T93I, and C97F were inactivated by Ai but not **NG-497** (Figure 5a). Variants I69F and T146G were neither inhibited by Ai nor by **NG-497** (Figure 5a). Based on these observations, we went on to test variants with combined aa exchanges and, strikingly, humanization of both I69F and T146G was sufficient to induce **NG-497** binding in mouse ATGL, suggesting that these two aa crucially determine species selectivity (Figure 6a). Nevertheless, the higher IC_50_ value of 9 μM observed for the double mutant indicated that **NG-497** binds the mutant with lower affinity than human ATGL (IC_50_ = 1.3 μM) (Figure 6b).

According to our 3D model, aa T93 and C97 are located in close spatial proximity to I69. We thus generated a quadruple mutant comprising mutations I69F/T93I/C97F/T146G and found that **NG-497** inhibits this mutant with similar IC_50_ values as detected for human ATGL (Figure 6b). Further analysis revealed that humanization of C97 to F97 (IC_50_ = 1 μM), but not T93 to I93 (IC_50_ = 9.3 μM), is sufficient to increase **NG-497** affinity (Figure 6c). According to our 3D models, the overall topologies of the minimal active domains of mouse and human ATGL are identical, with S47 and D166 as active site residues in close spatial proximity (depicted as green sticks, Figure 6d,f). Residues S10–L178 (depicted in blue for mouse and pink for human) build a relatively compact half of the protein, while residues K179–L254 (depicted in gray) are less compact. The minimal active domain of both mouse and human ATGL is predicted to have a large, two-pronged fork cavity. The “left,” shallow prong is formed mainly by residues S10–L17 and S170–L254. The “right,” deeper prong is formed by five different helices (depicted in light blue and pink for mouse and human ATGL, respectively). The first helix is assembled by the N-terminal residues G16-E31. A47–L104 form three other helices and residues L137–F147 assemble a short helix with an adjacent unstructured stretch. In the orientation shown in Figure 6d,f, the active site is located in the middle part of the cavity connecting both prongs to the exterior. I69, C97, T146, and their human counterparts F69, F97, and G146, are all located in the right prong of the cavity. T(G)146 is located near the active site, while I(F)69 and C(F)97 are located on the opposite side of the cavity in close spatial proximity (Figure 6e,g). Our 3D homology models indicate that these residues change the widths and depths of the right prong, which can explain their crucial contribution to inhibitor selectivity and efficacy.

## Discussion

Here, we report on the development and characterization of **NG-497**, a competitive small-molecule inhibitor targeting the enzymatically active patatin-like domain of human ATGL. **NG-497** completely abolishes hormone-stimulated FA release in human adipocytes demonstrating the critical role of ATGL in human lipolysis. HSL inhibition leads to accumulation of DAGs confirming its rate-limiting role in the second step of lipolysis. The application of ATGL inhibitors as scientific tools and their potential clinical use requires highly selective compounds. PNPLA family members exhibit high sequence and structural similarity with ATGL, particularly within the patatin-like domain.^34^ Since **NG-497** targets this domain, it is reasonable to assume that off-target inhibition most likely occurs in proteins with structural similarity catalyzing acylglycerol degradation. Accordingly, we first tested whether **NG-497** inhibits the most similar TAG hydrolases of the PNPLA family. Both PNPLA3 and PNPLA4 were previously reported to possess TAG hydrolase activity. PNPLA3 was identified as multiple risk allele for NAFLD development,^51^ while the pathophysiological relevance of PNPLA4 is unknown. We could not observe inhibition of these TAG hydrolases and we also could not observe inhibition of the more distantly related enzymes PNPLA6-9, which possess important functions in brain phospholipid metabolism,^39^ or PNPLA1, which has an essential role in skin lipid metabolism.^52^**NG-497** even selectively inhibited human and rhesus monkey ATGL, but not orthologues from other species. Furthermore, the inhibitor did neither inactivate other human intracellular acylglycerol hydrolases including CES2,^42^ DDHD2,^40^ and HSL^41^ nor the major circulating TAG hydrolases and pancreatic lipase. Additionally, untargeted lipidomic analysis of **NG-497**-treated cells revealed that most significant alterations occur in TAG and DAG species, arguing for on-target inhibition. Overall, our observations suggest that **NG-497** shows high selectivity for ATGL and does inactivate structurally and functionally related lipid hydrolases.

Based on the combined analysis of species-selective inhibitors and chimeric proteins, we were able to identify the molecular scaffold responsible for the high selectivity of **NG-497**. This scaffold comprises three aa within a hydrophobic cavity near the active site of ATGL. Our homology model suggests that these aa critically affect the size and shape of the cavity and thus determine inhibitor efficacy and selectivity. These observations provide a structural basis for further inhibitor development by rational design.

Accumulating evidence suggests that ATGL represents a promising pharmacological target for the treatment of common and rare disorders in humans. Inactivation of ATGL in adipose tissue by pharmacological and genetic approaches counteracts ectopic lipid accumulation and lipotoxicity in mice.^14−17,53^ ATGL inhibition thus represents a novel strategy for the treatment of lipotoxicity, which is frequently observed in obesity^54^ and very pronounced in several forms of congenital lipodystrophy.^55^ Additionally, several independent studies reported that adipose-specific deletion and pharmacological ATGL inactivation ameliorate experimental heart failure in mice.^25−29^ The beneficial effects of ATGL inhibition in this context are incompletely understood. Yet, heart failure is associated with an increased adrenergic drive compensating for reduced cardiac function.^30^ Elevated catecholamine levels also affect other organs and promote lipolysis in adipose tissue.^56^ It has been suggested that the clinical benefit of nonselective β-blockers, used for the treatment of cardiac insufficiency, partially derives from β-adrenergic receptor blockade on adipocytes resulting in reduced lipolysis.^26^ Additionally, changes in cardiac energy substrate availability and usage may represent potential protective mechanisms, as *Atgl* deletion reduces lipid oxidation and increases glucose disposal.^57^ This switch in substrate usage may have beneficial effects in the hypoxic heart since glucose utilization improves oxygen efficiency for ATP synthesis compared to FAs.^58^ A very recent study suggested the role of galectin-3 in Atglistatin-mediated cardioprotection.^27^ This protein belongs to the family of β-galactoside-binding proteins and is known to promote inflammation and fibrosis in the heart and other organs.^59^ Notably, ATGL inactivation reduced galectin-3 levels *in vivo* and ameliorated isoproterenol-induced galectin-3 secretion in adipose tissue organ cultures. The authors concluded that reduced galectin-3 secretion in response to ATGL inhibition contributes to the protective effects of Ai.^27^

Despite the beneficial effects of reduced lipolysis observed in disease models, inhibition of ATGL may also exert unfavorable effects. Since ATGL is rate-limiting for TAG hydrolysis in many tissues, a complete loss of ATGL activity leads to systemic TAG accumulation. Mice with global genetic *Atgl* deletion suffer from cardiomyopathy characterized by severe cardiac steatosis leading to premature death. Similarly, patients with loss-of-function mutations in the *ATGL* gene develop neutral lipid storage disease with myopathy (NLSDM), which is characterized by progressive skeletal- and cardiomyopathy in the adult state.^60,61^ Mouse studies suggested that myopathies are caused by defective mitochondrial function since ATGL mobilizes FAs required for activation of the PPAR-α–PGC-1 complex promoting mitochondrial biogenesis.^62^ In contrast to global deficiency, pharmacological inhibition of ATGL in mice does not lead to ectopic lipid accumulation or adverse effects in the heart even upon long-term treatment.^17^ It must be considered that pharmaceutical compounds allow the dose- and time-dependent inhibition of target proteins, which can avoid severe phenotypic abnormalities observed in global genetic deficiency. Ai is a competitive inhibitor of ATGL, leading to timely restricted inhibition of lipolysis.^31^ In mouse studies investigating the effect of Ai on heart failure and insulin sensitivity,^17,26,27,29^ the inhibitor was administered with the diet leading to postprandial suppression of ATGL activity. These observations indicate that partial inhibition of ATGL activity is sufficient for the beneficial effects in different disease models.

In recent years, the role of ATGL in the lipid metabolism of cancer cells has also gained attention.^63^ Cancer cells share the hallmark of metabolic reprogramming to sustain their high-energetic demand and proliferation rate, which includes a switch from oxidative to glycolytic energy production and aberrant fatty acid metabolism.^64^ ATGL provides fatty acids from endogenous TAG stores of cancer cells or tumor-surrounding tissues, which could be utilized for membrane synthesis in rapidly proliferating cells. However, ATGL is consistently less expressed in most investigated cancer specimens^65^ and silencing of ATGL has a minor effect on fatty acid metabolism and proliferation of cancer cell lines.^66^ Yet, ectopic overexpression of ATGL induces a glycolytic-to-oxidative metabolic switch in hepatocarcinoma cells and reduces the proliferation rate of several cancer cell lines.^66−68^ A very recent study has reported that loss of ATGL function causes a pro-Warburg effect in lung cancer cell spheroids but not in two-dimensional (2D) cell cultures.^69^ These studies indicate a tumor-suppressive function of ATGL. Conversely, other studies have suggested that elevated ATGL levels promote tumorigenesis of non-small-cell lung carcinomas and colon cancer cells.^70,71^ These controversial observations suggest an opposite role of ATGL in the different tumor types. The availability of **NG-497** will facilitate the investigation of the ATGL’s role in human cancer metabolism, growth, and malignancy.

## Conclusions

We demonstrate that **NG-497** acts as a selective, reversible, and nontoxic ATGL inhibitor, suitable for investigating ATGL function in human and nonhuman primate cells. Our data provide detailed insights into enzyme–inhibitor interactions, which can be used as a basis for further improvement of inhibitors by rational design. Current investigations focus on developing cross-species inhibitors allowing the detailed analysis of on- and off-target effects in rodent- and nonrodent animal models. Preclinical studies will unveil whether ATGL inhibitors allow safe inhibition of ATGL in humans.

Species-selective inhibition of orthologous enzymes is frequently observed for small-molecule inhibitors due to variations within binding scaffolds. In our study, we combined the analysis of species-selective inhibitors and chimeric enzymes for the detailed investigation of enzyme–inhibitor interactions. This procedure can be universally applied and represents an efficient tool for the identification of inhibitor binding sites. The described methodology could be highly relevant in the field of medicinal chemistry, especially when experimental 3D structures of the investigated proteins are not available.

## Supporting Information Available

Inhibition of semi-purified ATGL by **NG-497**; effect of **NG-497** on the protein–protein interaction of ATGL and G0S2; investigation of off-target effects of **NG-497**; scoreplot of the OPLS-DA model; effect of **NG-497** on lipolysis in human omental adipocytes; expression of chimeric ATGL proteins; overview and statistics of the lipidomic analysis; experimental details, materials, and methods; synthesis and full spectroscopic characterization of inhibitors; and NMR spectra for all compounds; supplemental references (PDF)
