# Supplementary material for: Small-Molecule Inhibitors Targeting Lipolysis in Human Adipocytes
Source: J Am Chem Soc. 2022 Apr 1;144(14):6237–50. doi: 10.1021/jacs.1c10836 (PMC9011347; doi:10.1021/jacs.1c10836)
Supplement: Supplementary file 1 — ja1c10836_si_001.pdf [file ja1c10836_si_001.pdf]

Small Molecule Inhibitors Targeting Lipolysis in Human Adipocytes

Gernot F. Grabner[1], Nikolaus Guttenberger[2], Nicole Mayer[2], Anna K. Migglautsch-Sulzer[2], Christian Lembacher-Fadum[2], Nermeen Fawzy[1], Dominik Bulfon[1], Peter Hofer[1], Thomas Züllig[1], Lennart Hartig[1], Natalia Kulminskaya[1], Gabriel Chalhoub[1], Margarita Schratter[1], Franz P.W. Radner[1], Karina Preiss-Landl[1], Sarah Masser[1], Achim Lass[1,3], Rudolf Zechner[1,3,4], Karl Gruber[1,3,4], Monika Oberer[1,3,4], Rolf Breinbauer[2,3,*], and Robert Zimmermann[1,3,4,*]

[1]Institute of Molecular Biosciences, University of Graz, Heinrichstrasse 31/2, 8010 Graz, Austria

[2]Institute of Organic Chemistry, Graz University of Technology, Stremayrgasse 9, 8010 Graz, Austria.

[3]BioTechMed-Graz, Mozartgasse 12/2, 8010 Graz, Austria.

[4]BioHealth Field of Excellence, University of Graz, Graz

[*]correspondence should be addressed to RB (breinbauer@tugraz.at) or RZi (robert.zimmermann@uni-graz.at).

SUPPORTING INFORMATION

TABLE OF CONTENTS **PAGE**

Supplemental Figures S1-S6 2-7
Supplemental Table S1 8
Experimental details, materials, and methods 14
Synthesis and full spectroscopic characterization of inhibitors 19
Supplemental references 36

Supplemental Figures S1-S6

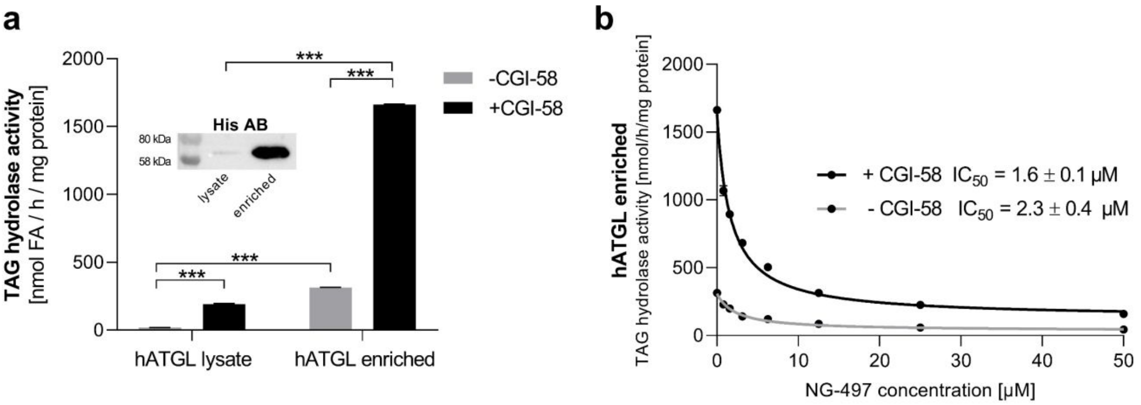

Fig. S1: Inhibition of semi-purified ATGL by NG-497. (a) His-tagged human ATGL was enriched from Expi293 lysates by affinity chromatography using ÄKTA pure chromatography system equipped with a His-Trap column. The enrichment from ATGL lysates was confirmed in TAG hydrolase assays and by Western blotting analysis (Insert) using an anti-His antibody. **(b) NG-497** inhibited semi-purified ATGL with similar IC_{50} values in the presence or absence of CGI-58 suggesting that the inhibitor directly targets ATGL. Statistical significance was determined via ANOVA followed by Bonferroni *post hoc* test (***, $p < 0.001$).

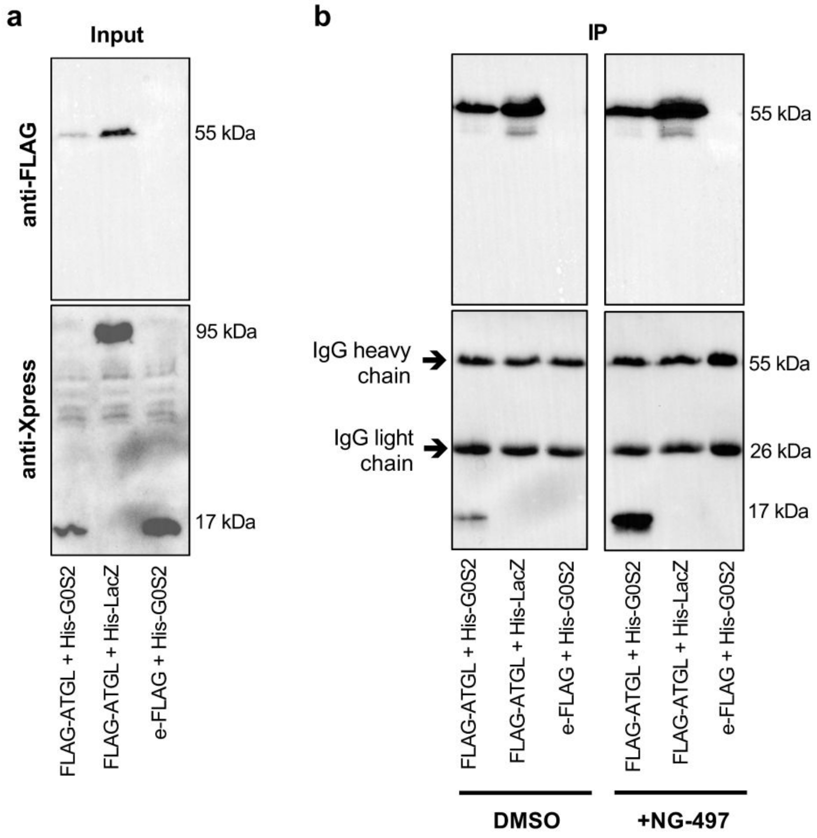

Fig. S2: Effect of NG-497 on the protein-protein interaction of ATGL and G0S2 investigated by co-immunoprecipitation. HEK293 cells were co-transfected with FLAG-tagged ATGL and His-tagged G0S2 (both human). As controls, FLAG-tagged ATGL was co-expressed with His-LacZ, and His-tagged G0S2 with empty FLAG-vector (e-FLAG). (**a**) Protein expression of the indicated plasmid combinations was confirmed using anti-Xpress and anti-Flag antibodies. (**b**) Cell lysates were incubated with anti-FLAG M2 affinity beads in the presence or absence of **NG-497** (40 µM). After extensive washing, bound proteins were eluted by boiling in SDS-containing sample buffer and subjected to Western blotting analysis. His-G0S2 was exclusively detected in the presence of ATGL and the addition of **NG-497** increased G0S2 binding. Data are representative for two independent experiments.

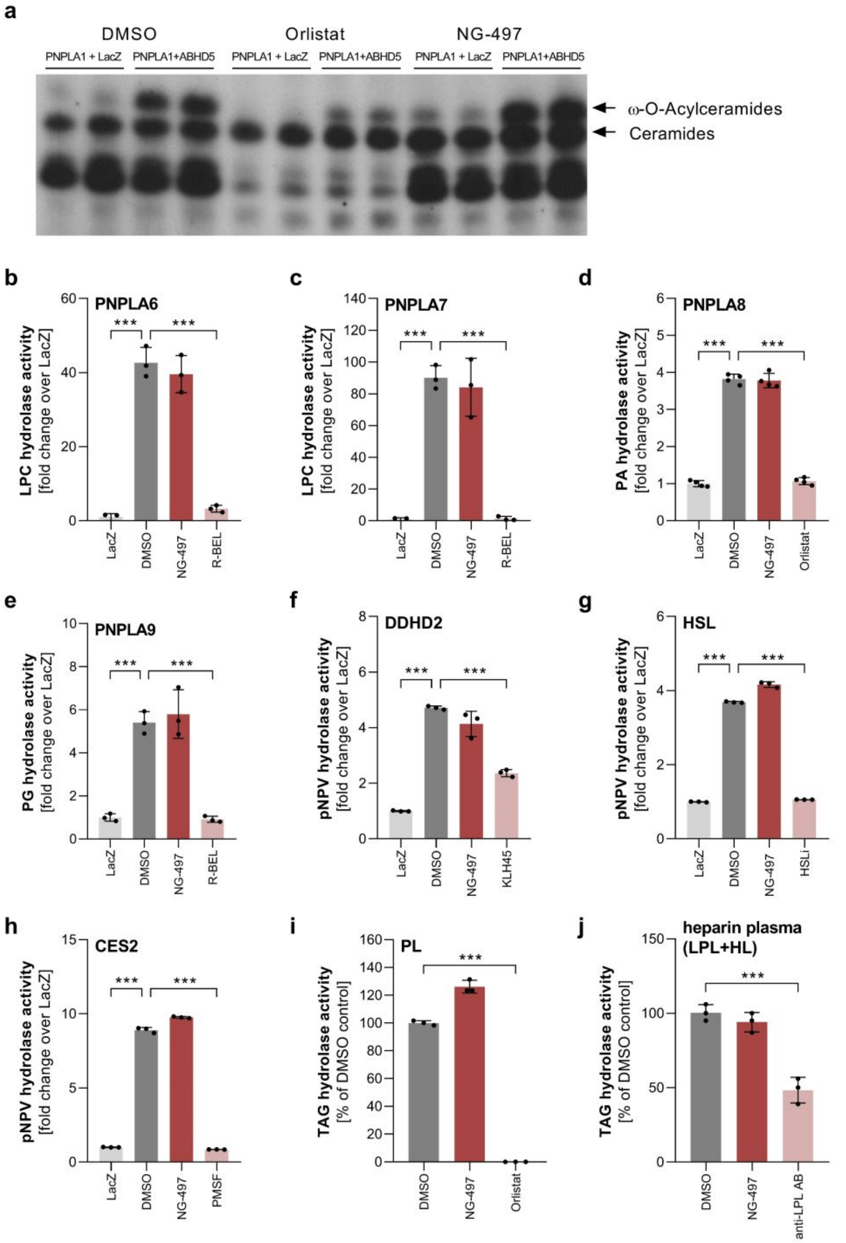

Fig. S3: Investigation of off-target effects of NG-497 on structurally and functionally related lipid hydrolases. (a) PNPLA1-dependent formation of acylceramides was monitored in a cell-based assay in HEK293T cells. Experiments were performed in the presence or absence of **NG-497** (40 µM) and the non-selective lipase inhibitor Orlistat (40 µM). Orlistat, but not **NG-497**, inhibited PNPLA1/CGI-58-dependent ω-*O*-acylceramide formation. (**b-h)** Enzymes were overexpressed in Expi293 cells and cell lysates were used as source of enzymatic activity. Cell lysates were incubated with the indicated substrates 1-oleoyl phosphatidylcholine (LPC), di-oleoyl phosphatidic acid (PA), di-octanoyl phosphatidylglycerol (PG), or para-nitrophenylvalerate (pNPV). Experiments were performed in the presence or absence of **NG-497** (100 µM) and the formation of reaction products was monitored. Cell lysates of LacZ-transfected cells were used to detect basal cellular activity. As positive controls, enzymes were inactivated with lipase inhibitors Orlistat (40 µM), R-BEL (10 µM), KLH-45 (20 nM), HSLi (10 µM), or PMSF (10µM). Data were calculated relative to the basal activity detected in LacZ-transfected controls. **(i)** Activity of purified human pancreatic lipase in the presence or absence of **NG-497** (100 µM) and Orlistat (20 µM) using radiolabeled triolein as substrate. **(j)** Inhibition of LPL and HL by **NG-497** (100 µM) was investigated in human post-heparin plasma using radiolabeled triolein as substrate. LPL activity was inhibited using an anti-LPL antibody. All data are presented as mean ± S.D. Statistical significance was determined via ANOVA followed by Bonferroni *post hoc* test (***, $p < 0.001$).

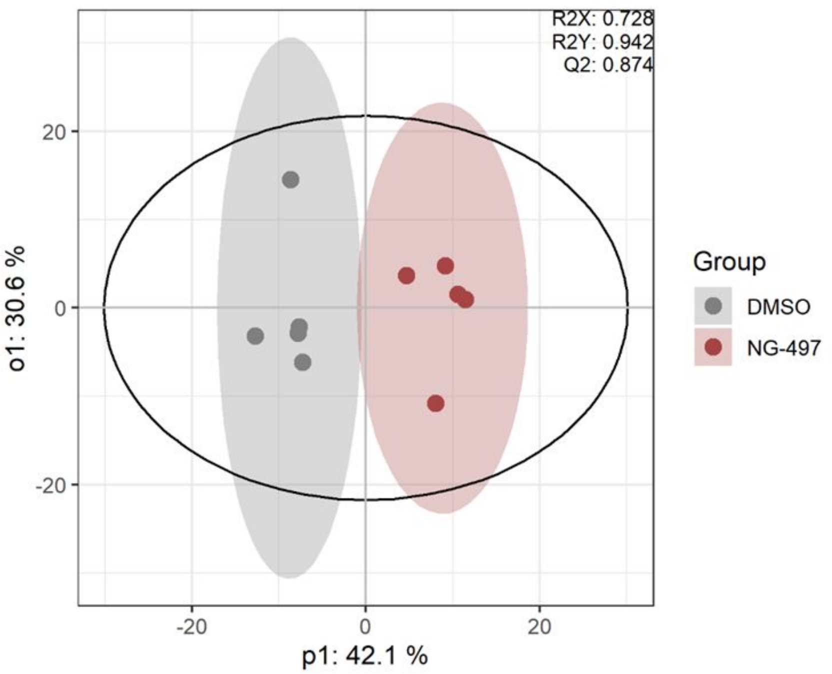

Fig. S4: Scoreplot of the OPLS-DA model obtained from 219 detected lipid species of the NG-497 treated group (red) vs. DMSO controls (grey). The within-group variation (o1) was 30.6% and the between-group variation 42.1% (p1). The diagnostic parameters of the model, the goodness of fit with 94.2% (R2Y(cum)) and the prediction goodness with 87.4% (Q2(cum)) are within the acceptable range. To avoid overfitting, PCA clustering was additionally checked before OPLS-DA analysis (data not shown).

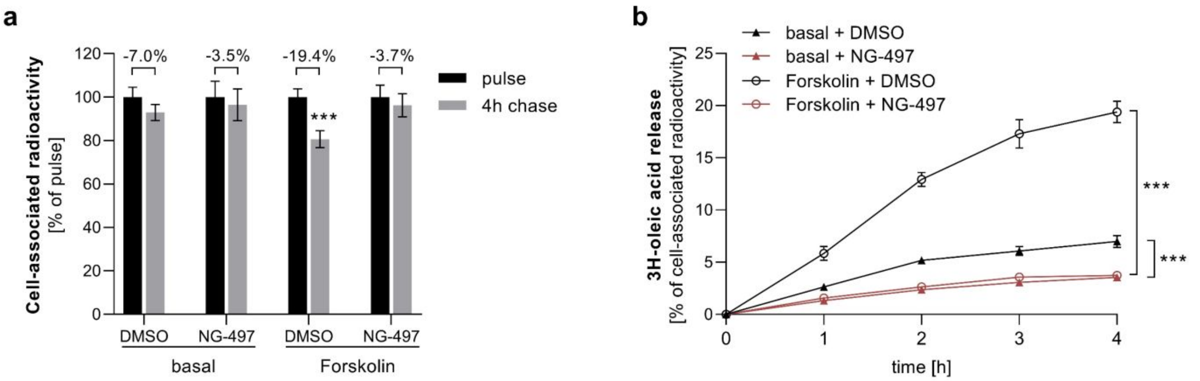

Fig. S5: Effect of NG-497 on lipolysis in human omental adipocytes. Omental preadipocytes were obtained from Zen-Bio and differentiated into adipocytes in 96-well trays according to the manufacturer's instructions (OP-F-SL, ZenBio). Cells were then labeled with [9,10-^{3}H] oleic acid (1µCi/well) in full medium for 12 h, washed, and preincubated for 1 h with **NG-497** (40 µM) or DMSO (carrier) as a control. Subsequently, the medium was replaced with DMEM containing 2% FA-free BSA and basal as well as Forskolin-stimulated release of radioactivity was monitored in the presence or absence of **NG-497**. After a 4 h incubation period, cells were lysed in NaOH/SDS (0.3 M/0.1%) and cell-associated radioactivity was determined by liquid scintillation. Cell-associated radioactivity at time-point 0 (pulse) was calculated as sum of the radioactivity detected in medium and cells after the 4 h chase. The release of fatty acids is shown as % of cell-associated radioactivity. **(a)** Cell-associated radioactivity did not significantly change under basal conditions during the 4 h incubation period. In the presence of Forskolin, cells lost ~ 20% of the radiolabel, which was completely prevented by the addition of **NG-497**. **(b)** Time course of FA release during the 4 h chase period. **NG-497** reduced basal lipolysis by ~50% and extinguished Forskolin-stimulated lipolysis. Data are presented as mean ± S.D (n = 4). Statistical significance was determined via ANOVA followed by Bonferroni *post hoc* test (***, $p < 0.001$).

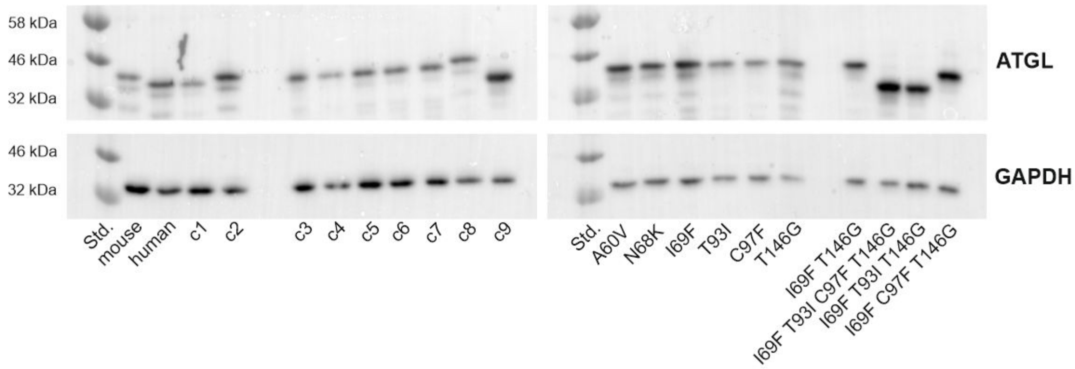

Fig. S6: Expression of chimeric ATGL proteins. Immunoblot of lysates from Expi293 cells expressing chimeric ATGL proteins using an anti-ATGL antibody. An anti-GAPDH antibody was used as loading control.

Table S1: Overview and statistics of the 219 lipid species detected in lipidomic analysis of **NG-497**-treated HepG2 cells (S6-S10) versus DMSO controls (S1-S5). The OPLS-DA ranking shows the lipid species ordered according to their contribution to the separation between the two groups. The significance level was tested with the nonparametric Wilcoxon test because a part of the data did not have a normal distribution, which was tested with the Shapiro-Wilk test (data not shown). Data are log2 transferred after normalization with cell protein and lipid class specific internal standards (IS). OPLS-DA ranking are the order of the contributing lipid species of the OPLS-DA model.

	DMSO (log2(AU/(protein*IS)))					**NG-497** (log2(AU/(protein*IS)))							
Lipid species	S1	S2	S3	S4	S5	S6	S7	S8	S9	S10	p-value	p-signif	OPLS-DA ranking
TAG 48:1	8.17	8.20	8.19	8.37	7.96	8.82	8.84	9.12	8.95	9.08	0.008	**	1
TAG 56:6	5.46	5.43	5.59	5.60	5.28	6.02	6.18	6.51	6.20	6.26	0.008	**	2
TAG 56:7	5.33	5.29	5.41	5.48	5.15	5.95	6.07	6.36	5.99	6.17	0.008	**	3
TAG 52:6	1.60	1.70	1.89	1.91	1.37	2.67	2.61	2.79	2.97	3.11	0.008	**	4
TAG 50:2	9.41	9.45	9.49	9.60	9.19	9.87	9.99	10.30	10.04	10.20	0.008	**	5
DAG 36:2	7.03	6.98	6.93	7.16	6.96	5.37	5.21	5.37	5.48	5.31	0.008	**	6
TAG 54:7	3.31	3.30	3.46	3.59	3.21	3.99	4.03	4.28	3.99	4.19	0.008	**	7
TAG 54:5	5.15	5.10	5.19	5.35	4.98	5.72	5.87	6.16	5.80	5.87	0.008	**	8
TAG 54:6	4.32	4.59	4.66	4.81	4.33	5.27	5.56	5.88	5.40	5.65	0.008	**	9
TAG 50:3	6.44	6.48	6.49	6.66	6.36	6.94	6.96	7.21	6.95	7.06	0.008	**	10
TAG 52:4	5.23	5.24	5.31	5.44	5.15	5.75	5.83	6.13	5.77	5.92	0.008	**	11
TAG 48:2	6.69	6.75	6.77	7.00	6.63	7.30	7.30	7.60	7.33	7.47	0.008	**	12
TAG 52:3	9.01	9.02	9.10	9.16	8.71	9.36	9.52	9.84	9.53	9.64	0.008	**	13
DAG 34:1	7.31	7.22	7.27	7.37	7.17	6.52	6.33	6.53	6.41	6.42	0.008	**	14
TAG 52:5	2.78	2.86	3.00	3.15	2.79	3.65	3.63	3.85	3.55	3.71	0.008	**	15
TAG 46:1	5.70	5.71	5.82	6.08	5.67	6.35	6.39	6.73	6.49	6.56	0.008	**	16
TAG 58:6	5.73	5.71	5.85	5.94	5.54	6.09	6.27	6.49	6.16	6.30	0.008	**	17
TAG 52:1	8.28	8.24	8.11	8.27	7.68	8.70	8.58	9.02	8.74	8.79	0.008	**	18
PI 36:1	3.26	3.21	3.06	3.15	2.57	3.97	3.65	4.04	4.55	4.24	0.008	**	19
TAG 54:4	7.00	7.02	7.08	7.19	6.84	7.35	7.49	7.88	7.44	7.59	0.008	**	20
LPC 18:0	4.54	4.55	4.49	4.88	4.70	5.55	5.27	5.58	5.72	5.67	0.008	**	21
TAG 56:5	5.60	5.65	5.79	5.86	5.48	6.15	6.29	6.56	6.06	6.25	0.008	**	22
TAG 53:4	2.78	2.83	2.93	2.99	2.77	3.25	3.38	3.69	3.27	3.33	0.008	**	23
DAG 32:1	5.69	5.59	5.67	5.89	5.64	4.82	4.88	5.09	4.82	4.92	0.008	**	24
TAG 58:7	5.89	5.90	6.01	6.11	5.73	6.30	6.43	6.68	6.24	6.44	0.008	**	25
Cer d34:1	7.33	7.44	7.35	7.64	7.35	8.37	8.20	8.22	8.27	8.04	0.008	**	26
TAG 51:2	6.08	6.09	6.14	6.34	6.03	6.39	6.53	6.89	6.54	6.63	0.008	**	27
TAG 44:0	3.16	3.21	3.21	3.65	3.32	3.91	3.87	4.04	4.04	3.99	0.008	**	28
PS 36:1	4.39	4.22	4.27	4.26	4.01	4.71	4.58	4.61	4.54	4.62	0.008	**	29
TAG 52:2	10.89	10.93	11.06	11.23	10.77	11.21	11.31	11.63	11.38	11.50	0.016	*	30
TAG 53:3	5.56	5.56	5.66	5.81	5.43	5.82	5.94	6.27	5.92	6.05	0.008	**	31
TAG 51:1	5.39	5.42	5.56	5.73	5.25	5.82	5.75	6.10	5.85	5.94	0.008	**	32
TAG 50:4	2.77	2.89	2.78	3.22	2.94	3.31	3.42	3.65	3.41	3.45	0.008	**	33
Cer d34:2	3.99	4.25	4.06	4.45	4.21	5.42	5.12	5.19	5.22	4.90	0.008	**	34
PI 34:1	3.96	4.09	4.31	4.55	4.16	4.88	4.63	4.81	4.91	4.91	0.008	**	35

LPC 16:0	6.23	6.38	6.11	6.65	6.38	7.12	6.74	7.05	7.29	7.12	0.008	**	36
TAG 49:1	5.00	5.01	5.02	5.29	4.99	5.29	5.39	5.79	5.41	5.47	0.008	**	37
TAG 54:2	9.08	8.99	9.04	9.05	8.45	9.23	9.29	9.76	9.32	9.43	0.008	**	38
TAG 58:8	5.04	5.03	5.16	5.28	4.94	5.41	5.60	5.83	5.32	5.45	0.008	**	39
PI 38:3	6.72	6.63	6.52	6.96	6.53	7.30	6.94	7.20	7.39	7.20	0.016	*	40
TAG 56:4	6.30	6.34	6.40	6.45	6.04	6.54	6.67	7.04	6.52	6.70	0.008	**	41
PI 38:2	4.76	4.78	5.12	4.87	4.75	5.48	5.22	5.26	5.78	5.41	0.008	**	42
PC 37:4	1.73	1.62	1.68	1.89	1.84	1.71	1.46	1.55	1.47	1.39	0.032	*	43
Cer d42:1	7.19	7.26	7.21	7.54	7.13	7.76	7.48	7.68	7.86	7.68	0.016	*	44
LPC 18:1	5.43	5.52	5.24	5.83	5.45	6.26	5.80	6.16	6.39	6.21	0.016	*	45
TAG 46:0	5.00	5.15	4.92	5.48	5.13	5.92	5.56	5.69	5.94	5.77	0.008	**	46
TAG 54:3	10.01	10.03	10.17	10.26	9.83	10.16	10.30	10.61	10.34	10.40	0.032	*	47
TAG 51:3	4.06	4.09	4.18	4.45	4.07	4.45	4.54	4.81	4.44	4.50	0.032	*	48
TAG 53:2	6.36	6.37	6.50	6.53	6.08	6.61	6.70	7.03	6.56	6.70	0.008	**	49
TAG 54:1	5.09	5.19	4.95	5.23	4.47	5.40	5.17	5.87	5.59	5.57	0.032	*	50
Cer d40:1	6.33	6.47	6.29	6.68	6.30	6.85	6.66	6.75	6.82	6.77	0.016	*	51
PS 38:4	2.66	2.49	2.60	2.61	2.49	2.94	2.62	2.81	2.91	3.00	0.016	*	52
TAG 60:7	5.45	5.50	5.54	5.64	5.23	5.69	5.82	6.10	5.62	5.74	0.016	*	53
TAG 56:0	6.27	6.31	6.28	6.31	5.61	6.41	6.39	7.20	6.66	6.73	0.008	**	54
TAG 56:3	7.82	7.73	7.81	7.72	7.19	7.89	7.97	8.50	7.91	8.11	0.008	**	55
PS 38:5	2.71	2.65	2.53	2.58	2.59	2.89	2.65	2.84	2.82	2.86	0.016	*	56
PC 35:3	2.03	2.05	1.98	2.39	2.14	2.00	1.76	1.92	1.81	1.70	0.016	*	57
TAG 60:6	4.22	4.19	4.14	4.24	3.70	4.48	4.52	5.03	4.24	4.55	0.016	*	58
TAG 53:1	3.54	3.76	3.53	3.68	3.19	3.99	3.55	4.14	3.92	4.28	0.032	*	59
PI 36:2	6.58	6.69	6.56	7.06	6.60	7.28	6.97	7.21	7.44	7.07	0.016	*	60
TAG 42:0	1.11	1.27	1.01	1.72	1.47	1.69	1.83	2.21	1.87	1.88	0.016	*	61
PC 40:6	3.57	3.54	3.52	3.90	3.69	3.42	3.36	3.46	3.39	3.38	0.008	**	62
LPC 20:1	1.89	2.07	1.80	2.31	2.11	2.74	2.20	2.69	2.93	2.65	0.016	*	63
TAG 58:4	4.59	4.44	4.56	4.56	3.97	4.66	4.77	5.34	4.62	4.91	0.008	**	64
TAG 58:5	5.01	4.63	4.63	4.75	4.21	4.97	5.05	5.66	4.92	5.10	0.032	*	65
PC 35:2	4.77	4.80	4.75	5.09	4.90	4.80	4.50	4.72	4.57	4.46	0.032	*	66
Cer d41:1	5.42	5.62	5.53	5.72	5.41	5.85	5.68	5.76	5.99	5.78	0.016	*	67
PC 36:0	-0.20	-0.13	-0.35	0.05	-0.31	0.13	-0.09	0.15	0.20	0.09	0.016	*	68
LPC 16:1	2.98	3.26	2.99	3.55	3.15	3.82	3.38	3.71	3.84	3.71	0.016	*	69
TAG 55:4	3.37	3.46	3.58	3.82	3.33	3.69	3.74	4.14	3.74	3.87	0.056	ns	70
TAG 56:2	6.38	6.26	6.32	6.28	5.56	6.35	6.33	7.18	6.63	6.74	0.032	*	71
TAG 48:3	3.70	3.73	3.84	4.25	3.89	4.17	4.30	4.55	4.16	4.26	0.032	*	72
PC 44:7	-0.60	-0.57	-0.57	-0.25	-0.49	-0.55	-0.93	-0.78	-0.75	-0.72	0.056	ns	73
TAG 55:2	4.30	4.05	3.78	3.86	3.59	4.29	4.36	4.84	4.04	4.34	0.056	ns	74
PC 36:3	7.17	7.11	7.13	7.48	7.25	7.19	6.92	7.06	6.96	6.90	0.056	ns	75
Cer d42:2	8.41	8.53	8.42	8.77	8.39	8.85	8.64	8.78	8.86	8.73	0.032	*	76
Cer d40:2	5.70	5.69	5.60	5.97	5.64	6.08	5.87	5.93	6.15	5.99	0.032	*	77

TAG 60:4	3.31	3.07	3.23	3.17	2.47	3.14	3.23	4.07	3.41	3.70	0.151	ns	78
Cer d42:3	6.57	6.69	6.58	6.93	6.63	7.06	6.86	6.88	7.09	6.96	0.032	*	79
TAG 58:2	5.77	5.73	5.67	5.69	4.84	5.67	5.64	6.59	6.09	6.21	0.310	ns	80
PC 39:6	0.23	0.21	0.35	0.64	0.33	0.12	0.14	-0.18	0.19	0.07	0.008	**	81
DAG 32:0	6.28	6.25	6.17	6.48	6.16	6.01	5.88	6.15	5.99	5.99	0.008	**	82
PS 36:4	2.79	2.69	2.61	2.79	2.68	3.14	2.76	3.04	2.94	2.92	0.032	*	83
PC 34:4	1.37	1.30	1.27	1.67	1.48	1.37	1.02	1.27	1.12	1.14	0.056	ns	84
DAG 34:2	5.66	5.63	5.35	5.05	4.71	4.56	4.20	4.66	4.63	4.40	0.008	**	85
SM d41:0	1.65	1.47	1.69	1.96	1.67	1.91	1.88	1.86	2.07	2.05	0.056	ns	86
SM d34:2	6.45	6.44	6.43	6.74	6.55	6.78	6.59	6.69	6.95	6.99	0.032	*	87
Cer d36:1	4.87	4.89	4.86	5.20	4.84	5.19	5.09	5.13	5.27	5.12	0.095	ns	88
PE 32:1	6.46	6.52	6.29	6.86	6.48	6.93	6.63	6.79	7.11	7.06	0.032	*	89
TAG 55:3	5.31	5.26	5.30	5.42	4.93	5.34	5.44	5.90	5.35	5.52	0.032	*	90
TAG 58:3	5.58	5.75	5.54	5.45	4.83	5.52	5.56	6.43	5.81	5.95	0.151	ns	91
TAG 58:1	3.99	4.25	3.71	4.28	3.35	4.15	3.90	4.76	4.63	4.59	0.151	ns	92
TAG 60:3	5.00	4.93	4.82	4.76	4.06	4.77	4.87	5.74	5.09	5.29	0.151	ns	93
TAG 62:4	1.94	1.92	1.70	1.78	1.28	1.78	1.77	2.78	2.10	2.31	0.222	ns	94
LPC 14:0	2.28	2.44	2.22	2.76	2.34	2.93	2.46	2.82	2.98	2.84	0.016	*	95
SM d42:0	2.42	2.47	2.35	2.85	2.33	2.73	2.63	2.65	3.10	2.99	0.056	ns	96
PC 37:3	2.01	1.88	1.97	2.38	2.05	1.99	1.68	1.89	1.81	1.64	0.056	ns	97
PS 40:7	2.46	2.34	2.26	2.61	2.58	2.41	2.05	2.45	2.01	2.01	0.095	ns	98
TAG 62:3	3.22	3.24	3.15	3.18	2.24	3.01	3.09	4.09	3.47	3.79	0.421	ns	99
PC 33:2	3.19	3.25	3.11	3.52	3.34	3.25	2.93	3.15	3.10	2.98	0.056	ns	100
PC 36:2	9.00	9.03	9.02	9.31	9.08	9.07	8.82	8.99	8.87	8.78	0.056	ns	101
PI 40:5	3.96	4.10	4.14	4.49	3.98	4.50	4.17	4.40	4.58	4.56	0.032	*	102
SM d42:1	6.80	6.74	6.71	7.05	6.77	7.02	6.84	6.95	7.28	7.30	0.056	ns	103
PI 38:4	8.33	8.44	8.30	8.73	8.22	8.71	8.44	8.62	8.95	8.77	0.095	ns	104
PC 34:3	4.49	4.55	4.45	4.79	4.61	4.55	4.28	4.46	4.43	4.34	0.095	ns	105
PC 42:5	0.54	0.45	0.44	0.60	0.57	0.46	0.16	0.34	0.44	0.45	0.056	ns	106
PE 32:2	3.94	3.95	3.70	4.14	3.85	4.25	3.86	4.14	4.49	4.40	0.056	ns	107
SM d41:1	5.27	5.21	5.23	5.54	5.27	5.48	5.34	5.40	5.72	5.73	0.056	ns	108
PC 33:1	5.62	5.65	5.59	5.92	5.73	5.69	5.45	5.62	5.55	5.43	0.095	ns	109
Cer d41:2	5.15	5.26	4.86	5.49	5.35	5.63	5.49	5.56	5.59	5.51	0.008	**	110
SM d40:0	3.45	3.37	3.30	3.79	3.53	3.73	3.57	3.68	3.94	3.91	0.056	ns	111
PC 42:1	-1.27	-1.36	-1.38	-1.06	-1.21	-0.85	-1.23	-0.96	-0.94	-1.02	0.032	*	112
PC 34:2	8.76	8.78	8.71	9.07	8.85	8.83	8.54	8.76	8.63	8.54	0.151	ns	113
SM d32:1	6.35	6.34	6.30	6.65	6.45	6.61	6.46	6.52	6.87	6.89	0.056	ns	114
SM d38:0	3.09	3.03	2.93	3.40	3.11	3.27	3.16	3.29	3.54	3.48	0.056	ns	115
SM d40:1	7.00	6.95	6.91	7.24	6.95	7.18	7.00	7.12	7.45	7.43	0.095	ns	116
SM d32:0	3.20	3.17	3.01	3.49	3.22	3.52	3.29	3.29	3.67	3.76	0.032	*	117
PC 40:1	-0.08	-0.11	-0.17	0.22	-0.04	0.28	0.03	0.19	0.19	0.16	0.095	ns	118
PC 31:1	3.38	3.39	3.32	3.67	3.50	3.47	3.14	3.34	3.33	3.21	0.151	ns	119

PC 34:1	9.98	9.99	9.97	10.35	10.09	10.11	9.84	10.02	9.83	9.70	0.310	ns	120
SM d42:2	8.78	8.72	8.68	9.02	8.74	8.95	8.76	8.90	9.20	9.16	0.095	ns	121
TAG 49:2	4.46	4.50	4.52	4.90	4.61	4.79	4.84	5.15	4.71	4.73	0.095	ns	122
SM d43:1	2.49	2.54	2.52	2.87	2.68	2.77	2.68	2.76	3.01	2.89	0.095	ns	123
Cer d38:1	4.76	4.80	4.19	4.92	4.70	5.17	4.82	5.00	5.25	4.95	0.016	*	124
PC 32:0	7.71	7.74	7.68	8.01	7.79	8.20	7.86	8.07	8.03	7.89	0.032	*	125
PE 33:3	3.98	3.96	3.74	4.24	4.04	4.17	3.93	4.11	5.13	5.03	0.222	ns	126
PC 39:4	-0.12	0.03	0.11	0.19	0.20	0.06	-0.06	0.00	0.03	-0.12	0.151	ns	127
PI 38:5	7.05	6.97	6.94	7.31	6.98	7.34	7.02	7.17	7.59	7.35	0.056	ns	128
PC 38:6	5.41	5.41	5.37	5.66	5.50	5.52	5.30	5.39	5.39	5.20	0.222	ns	129
SM d38:1	5.97	5.93	5.89	6.23	5.96	6.15	5.96	6.08	6.34	6.37	0.095	ns	130
PC 38:7	1.76	1.80	1.70	2.11	1.92	1.90	1.59	1.76	1.72	1.61	0.151	ns	131
PC 32:2	6.00	6.06	5.93	6.27	6.09	6.09	5.83	6.00	5.93	5.89	0.222	ns	132
PE 34:3	4.16	4.38	4.12	4.72	4.34	4.63	4.37	4.54	4.82	4.72	0.095	ns	133
SM d44:2	2.33	2.23	2.38	2.59	2.41	2.56	2.36	2.56	2.57	2.64	0.222	ns	134
PC 39:3	-0.47	-0.48	-0.39	-0.16	-0.24	-0.23	-0.63	-0.41	-0.49	-0.66	0.222	ns	135
PE 34:2	8.09	8.16	7.96	8.47	8.05	8.41	8.11	8.25	8.58	8.54	0.095	ns	136
PI 38:6	4.20	4.09	4.11	4.23	4.03	4.41	4.06	4.20	4.70	4.36	0.151	ns	137
PC 38:4	5.89	5.81	5.90	6.15	5.93	6.01	5.67	5.86	5.80	5.69	0.151	ns	138
PC 44:8	-1.26	-1.18	-0.93	-0.61	-1.02	-1.02	-1.65	-1.26	-1.06	-1.37	0.222	ns	139
PC 32:1	9.32	9.36	9.26	9.65	9.42	9.44	9.14	9.36	9.26	9.13	0.421	ns	140
SM d41:2	5.48	5.49	5.43	5.80	5.52	5.69	5.49	5.64	5.90	5.89	0.095	ns	141
SM d34:0	6.88	6.87	6.77	7.11	6.88	7.06	6.88	6.99	7.13	7.16	0.151	ns	142
TAG 62:2	2.00	2.11	1.87	2.56	1.17	1.70	1.70	3.05	2.63	2.67	0.421	ns	143
TAG 56:1	4.40	4.25	3.95	4.52	3.85	4.49	3.93	4.70	4.86	4.53	0.151	ns	144
SM d33:1	6.10	6.10	6.01	6.43	6.14	6.32	6.08	6.25	6.51	6.57	0.222	ns	145
PE 36:2	9.05	9.09	8.89	9.42	8.95	9.35	9.01	9.21	9.50	9.42	0.151	ns	146
PE 40:6	7.06	7.06	6.91	7.45	6.97	7.46	7.08	7.17	7.51	7.40	0.056	ns	147
PS 36:3	3.68	3.52	3.60	3.73	3.66	3.69	3.53	3.66	4.19	4.23	0.310	ns	148
PC 37:2	2.92	2.94	2.89	3.25	3.03	2.96	2.71	2.94	2.94	2.87	0.421	ns	149
PC 35:1	4.35	4.34	4.34	4.67	4.44	4.44	4.19	4.39	4.34	4.25	0.310	ns	150
PC 39:5	0.21	0.21	0.29	0.61	0.34	0.36	0.15	0.22	0.32	0.01	0.548	ns	151
SM d34:1	10.54	10.54	10.48	10.81	10.60	10.76	10.51	10.67	10.90	10.87	0.222	ns	152
TAG 44:1	3.36	3.59	3.60	4.17	3.98	3.94	4.17	4.39	3.89	3.90	0.310	ns	153
SM d40:2	6.22	6.21	6.19	6.51	6.28	6.46	6.23	6.33	6.54	6.56	0.095	ns	154
PE 38:4	8.52	8.51	8.34	8.88	8.40	8.83	8.48	8.61	8.90	8.79	0.222	ns	155
PI 40:6	4.11	4.10	4.05	4.38	3.94	4.33	4.10	4.12	4.47	4.30	0.222	ns	156
PE 34:1	7.58	7.62	7.42	7.93	7.47	7.83	7.50	7.67	7.98	7.92	0.222	ns	157
PC 32:3	0.06	0.15	-0.01	0.49	0.26	0.15	-0.17	0.02	0.19	0.06	0.548	ns	158
PC 36:4	5.88	5.87	5.85	6.15	5.99	6.04	5.81	5.94	5.81	5.76	0.310	ns	159
TAG 48:0	5.97	6.20	5.69	6.61	6.21	6.97	6.21	6.18	6.92	6.50	0.222	ns	160
PS 38:3	3.92	3.81	3.83	3.90	3.65	4.05	3.90	3.90	3.82	3.86	0.548	ns	161

SM d32:2	1.83	1.82	1.78	2.08	1.83	1.81	1.70	1.59	1.90	1.84	0.548	ns	162
PC 36:5	2.20	2.30	2.29	2.66	2.42	2.50	2.27	2.30	2.25	2.13	0.421	ns	163
PC 38:3	2.93	2.95	2.70	3.19	3.04	2.97	2.80	2.86	2.88	2.89	0.310	ns	164
PC 42:7	1.88	1.88	1.81	2.24	1.96	2.05	1.69	1.87	1.89	1.80	0.421	ns	165
PC 42:9	-0.45	-0.06	-0.21	0.08	-0.29	-0.20	-0.55	-0.19	-0.45	-0.29	0.421	ns	166
PI 36:4	5.72	5.71	5.57	5.99	5.50	5.84	5.57	5.75	6.13	5.86	0.310	ns	167
PC 39:2	-0.75	-0.81	-0.82	-0.48	-0.54	-0.72	-0.95	-0.70	-0.64	-0.79	0.841	ns	168
SM d43:2	3.97	3.88	3.91	4.02	4.01	4.20	4.01	3.87	4.13	4.11	0.310	ns	169
TAG 64:8	2.77	2.75	2.82	2.98	2.35	2.81	2.85	3.33	2.51	2.79	0.548	ns	170
TAG 47:2	3.10	2.94	3.33	3.73	3.25	3.19	3.22	3.56	2.59	2.74	0.421	ns	171
TAG 46:2	4.23	4.16	5.05	4.84	4.23	4.47	4.56	5.26	4.59	4.54	0.421	ns	172
PC 37:1	0.82	0.82	0.74	1.15	0.86	0.91	0.57	0.86	0.84	0.78	0.841	ns	173
PC 44:6	-0.44	-0.44	-0.61	-0.09	-0.39	-0.27	-0.65	-0.42	-0.44	-0.59	0.690	ns	174
PE 37:4	3.61	3.59	3.29	3.73	3.43	3.77	3.31	3.67	3.80	3.67	0.222	ns	175
PS 40:8	1.07	1.13	1.03	1.24	0.93	1.35	0.74	1.20	1.28	1.35	0.222	ns	176
PE 36:5	6.35	6.43	6.19	6.82	6.36	6.64	6.20	6.48	6.78	6.73	0.421	ns	177
PC 33:0	2.77	2.75	2.79	3.14	2.85	2.84	2.73	2.92	2.79	2.69	0.690	ns	178
SM d38:2	3.95	3.79	3.87	4.24	4.03	4.01	3.88	3.90	3.96	3.87	1.000	ns	179
PC 42:10	-1.88	-1.76	-1.93	-1.32	-1.70	-1.49	-1.67	-1.40	-1.69	-1.69	0.151	ns	180
PC 30:0	7.73	7.77	7.65	8.04	7.82	8.05	7.73	7.95	7.95	7.83	0.310	ns	181
PC 31:0	3.76	3.73	3.69	4.06	3.83	3.95	3.65	3.85	3.74	3.64	0.690	ns	182
PS 32:1	2.32	2.16	2.18	2.27	2.02	2.55	2.32	2.49	2.05	2.09	0.548	ns	183
SM d36:1	6.94	6.96	6.89	7.20	6.92	7.05	6.88	6.99	7.15	7.10	0.548	ns	184
PE 40:7	7.66	7.71	7.56	8.08	7.64	7.92	7.56	7.69	8.05	7.94	0.690	ns	185
PS 34:1	4.27	4.18	3.91	4.06	3.95	4.46	4.12	4.30	4.03	4.00	0.421	ns	186
PC 42:2	-0.15	-0.41	-0.14	-0.07	-0.26	0.18	-0.33	-0.01	0.01	-0.29	0.421	ns	187
PC 30:1	5.57	5.65	5.52	5.94	5.68	5.74	5.46	5.67	5.66	5.63	1.000	ns	188
PC 42:8	-1.04	-0.99	-0.91	-0.02	-0.84	-0.37	-0.94	-0.78	-0.44	-0.47	0.310	ns	189
PC 40:3	1.16	1.09	1.05	1.46	1.24	1.32	0.97	1.20	1.22	1.12	1.000	ns	190
SM d41:3	2.63	2.63	2.57	2.90	3.01	2.82	2.64	2.63	2.86	2.72	1.000	ns	191
PE 40:5	5.98	5.82	6.03	6.41	5.98	6.21	6.06	5.99	6.35	6.04	0.222	ns	192
PC 40:7	4.27	4.31	4.18	4.54	4.39	4.57	4.20	4.35	4.33	4.20	1.000	ns	193
PE 36:3	6.93	7.03	6.87	7.45	6.94	7.23	6.87	6.96	7.41	7.15	0.548	ns	194
PC 42:3	-1.19	-1.19	-1.13	-0.78	-0.94	-0.89	-1.12	-1.09	-1.05	-1.13	0.690	ns	195
PC 29:0	1.99	2.09	1.96	2.26	2.12	2.21	1.97	2.04	2.15	2.01	1.000	ns	196
PC 42:4	-0.86	-0.68	-1.00	-0.67	-0.77	-0.79	-0.94	-0.77	-0.89	-0.76	0.690	ns	197
PS 40:6	4.67	4.51	4.55	4.53	4.40	4.75	4.48	4.50	4.41	4.43	0.548	ns	198
PS 38:6	1.86	1.95	1.93	1.86	1.70	2.16	1.85	1.98	1.74	1.80	1.000	ns	199
PE 38:5	8.50	8.69	8.35	9.05	8.45	8.87	8.54	8.48	8.81	8.72	0.421	ns	200
PS 40:5	3.59	3.39	3.61	3.56	3.35	3.70	3.48	3.52	3.36	3.59	1.000	ns	201
PC 38:5	5.44	5.43	5.37	5.66	5.47	5.75	5.40	5.62	5.51	5.39	0.690	ns	202
PC 38:1	1.73	1.71	1.58	2.03	1.75	1.90	1.60	1.84	1.86	1.81	0.421	ns	203

PS 42:9	2.93	2.89	2.98	3.14	2.86	3.13	2.65	3.01	3.12	3.04	0.548	ns	204
SM d36:2	5.02	4.98	4.95	5.33	5.08	5.16	4.93	5.04	5.20	5.18	0.690	ns	205
TAG 47:1	3.84	3.89	4.02	4.30	4.12	4.14	4.11	4.47	3.82	3.91	0.841	ns	206
PC 40:4	1.98	2.16	2.11	2.30	2.27	2.33	2.00	2.20	2.21	2.11	0.841	ns	207
PC 40:8	-0.42	-0.34	-0.30	-0.02	-0.22	-0.04	-0.27	-0.10	-0.23	-0.39	0.690	ns	208
PC 44:5	-1.88	-2.06	-2.04	-1.72	-1.47	-1.62	-2.01	-1.73	-1.80	-1.86	0.690	ns	209
PI 40:7	4.03	3.98	3.81	4.33	3.79	4.09	3.78	3.78	4.13	4.01	0.841	ns	210
PS 34:2	2.44	2.32	2.31	2.46	2.16	2.68	2.34	2.57	1.93	2.12	1.000	ns	211
PE 38:6	7.39	7.63	7.23	7.86	7.36	7.58	7.14	7.48	8.00	7.43	0.841	ns	212
PE 38:7	4.32	4.42	4.10	4.72	4.32	4.52	3.95	4.22	4.62	4.47	1.000	ns	213
PC 40:5	3.75	3.72	3.71	4.02	3.82	3.92	3.63	3.80	3.90	3.79	0.841	ns	214
PE 36:4	7.02	7.13	6.92	7.50	7.05	7.19	6.97	7.03	7.35	7.18	0.690	ns	215
PC 36:1	6.36	6.34	6.28	6.64	6.42	6.51	6.21	6.46	6.53	6.43	0.548	ns	216
PC 40:2	1.03	1.01	1.06	1.37	1.11	1.23	1.02	1.20	1.13	1.09	0.548	ns	217
SM d36:0	2.79	2.85	2.99	3.03	2.74	3.09	3.09	2.99	2.47	2.80	0.690	ns	218
PS 36:2	3.83	3.70	3.73	3.85	3.62	3.98	3.78	3.92	3.52	3.60	1.000	ns	219

Experimental details, materials, and methods

Expression of recombinant ATGL constructs and preparation of cell lysates. Expi293 cells were cultivated in Expi293 Expression Medium (GIBCO, Thermo Fisher Scientific, Waltham, USA) at standard conditions (37°C, 5% CO_2, 95% humidified atmosphere, 125 rpm). DNA constructs containing ATGL from different species and chimeric ATGL variants in pcDNA3.1(+)myc-His C Vector were obtained from BioCat GmbH (Heidelberg, Germany). Cells were transfected with ExpiFectamine293 Transfection Kit (GIBCO) in Opti-Mem reduced serum medium (GIBCO) according to the manufacturer's instructions. After 48 h, cells were harvested by centrifugation and washed three times with PBS. Cells were disrupted in buffer A (0.25 M sucrose, 1 mM EDTA, 1 mM dithiothreitol, 20µg/ml leupeptin, 2 µg/ml antipain, 1 µg/ml pepstatin, pH 7.0) by sonication (Virsonic 475, Virtis, Gardiner, USA). Nuclei and debris were removed by centrifugation at 1,000 g and 4°C for 10 min. Protein concentrations were determined using Bio-Rad protein assay (Bio-Rad 785, Bio-Rad Laboratories, Munich, Germany) using BSA as standard.

Expression and purification of mouse CGI-58. CGI-58 was expressed and purified as described [1]. In brief, the coding sequence of mouse *Cgi-58* was cloned into pHis-Sumo and co-transformed with the plasmid pTf16 expressing the chaperone Tig into *E. coli* BL21(DE3). Cells were cultured overnight at 37°C and 180 rpm in the presence of 40 µg/ml kanamycin and 34 µg/ml chloramphenicol. Thereafter, cells were transferred into fresh LB medium, grown until OD_{600} of 0.6, and expression was induced by the addition of 0.5 mM IPTG. After overnight incubation at 30°C, cells were harvested at 4,500 x g and 4°C for 20 min, washed in 1x PBS, and resuspended in lysis buffer (20 mM Tris/HCl pH 7.8, 300 mM NaCl, 30 mM imidazole, 0.1% IGEPAL CA-630, 3.5 mM β-mercaptoethanol, 20 µg/ml leupeptin, 2 µg/ml antipain and 1 µg/ml pepstatin). Cells were incubated with 1 mg/ml lysozyme at 4°C for 20 min, disrupted by sonication, and lysates were centrifuged at 30,000 x g and 4°C for 40 min. His-CGI-58 was purified from the supernatant using an ÄKTA pure system (Cytiva, Uppsala, Sweden) equipped with a 5 ml HisTrap HP column.

Expression and purification of mouse G0S2. Chemically competent *E. coli* ArcticExpress (DE3) cells were transformed with the pST50-SMT3-TEV-mG0S2-His expression plasmid, plated on LB agar plates containing 100 µg/ml ampicillin, and incubated overnight at 37°C. The next day, 10 ml LB medium supplemented with 100 µg/ml ampicillin, 20 µg/ml gentamycin and 2% glucose were inoculated with a single colony and incubated overnight at 37°C. The following day, fresh LB medium was supplemented with 0.5% glucose, 10 mM $MgCl_2$, and 300 µl of silicone oil, and inoculated with the total volume of the ONC. No selection antibiotics were added to the expression cultures. , The cultures were incubated at 30°C and 120 rpm until they reached an OD_{600} of 0.5, to induce then protein expression by the addition of 0.1 mM IPTG at 10°C for 24 h. Cells were harvested by centrifugation at 6,000 x g and 4°C for 10 min and lysed by sonication in lysis buffer (20 mM Tris/HCl pH 7.8, 300 mM NaCl, 30 mM imidazole, 0.1% (v/v) IGEPAL CA-630, 1 mM TCEP) supplemented with 1 mM benzamidine and 0.1 mM PMSF. Soluble fractions of mouse G0S2 were loaded onto a 5 ml HisTrap HP column in an ÄKTA pure system (Cytiva). An additional washing step with 10 CV of wash buffer (20 mM TrisHCl pH 7.8, 300 mM NaCl, 30 mM imidazole, 10% (v/v) glycerol, 1 mM TCEP) was performed to remove residual IGEPAL CA-630. Proteins were eluted in a gradient of elution buffer (20 mM Tris/HCl pH 7.8, 300 mM NaCl, 250 mM imidazole, 10% (v/v) glycerol, 1 mM TCEP).

Immunoblotting. For expression analysis, 10 µg of protein per lane were subjected to SDS-PAGE, transferred to polyvinylidene fluoride (PVDF) transfer membrane (Roth GmbH, Karlsruhe, Germany) via Trans-Blot Turbo Transfer System (Bio-Rad), and blocked with 10% blotting grade milk powder (Roth) in TST (50 mM Tris/HCl, pH 7.4, 0.15 M NaCl, 0.1% Tween-20). Membranes were incubated with antibodies against ATGL (#2138S, CST, Danvers, USA), GAPDH (#2118S, CST), or His-Tag (#18184, Abcam, Cambridge, UK) prepared in 5% milk powder in TST. Antibody binding was detected with anti-rabbit (#7074, CST) or anti-mouse (#NA931V, GE Healthcare) HRP-linked secondary antibodies in 5% milk powder in TST and visualized using Clarity Western ECL Substrate and a ChemiDoc Touch Imaging System (both Bio-Rad).

Determination of TAG hydrolase activity. For the determination of TAG hydrolase activity, cell lysates in a total volume of 25 µl buffer A (0.25 M sucrose, 1 mM EDTA, 1 mM dithiothreitol, 20 µg/ml leupeptin, 2 µg/ml antipain, 1 µg/ml pepstatin, pH 7.0) were incubated with 25 µl substrate in a water bath at 37°C for 60 min. Substrate was prepared by emulsifying 330 µM triolein (40,000 cpm/nmol) and 45 µM phosphatidylcholine/phosphatidylinositol (3:1) in 100 mM potassium phosphate buffer (pH 7.0) by sonication and adjusted to 5% essentially FA-free BSA (MilliporeSigma, St. Louis, USA). After incubation, the reaction was terminated by adding 650 µl of methanol/chloroform/heptane (10:9:7) and 200 µl of 0.1 M potassium carbonate, 0.1 M boric acid (pH 10.5). After centrifugation at 800 x g for 15 min, the radioactivity in 200 µl of the upper phase was determined by liquid scintillation using Rotiszint eco plus (Roth) and a Tri-Carb 2900TR liquid scintillation analyzer (PerkinElmer, Waltham, USA). Counts from control incubations containing buffer A alone were subtracted and the rate of FA hydrolysis was calculated based on the specific radioactivity of the triolein substrate.

Co-immunoprecipitation. HEK293 cells were co-transfected with the indicated plasmid combinations using Metafectene (#T020-5.0; Biontex, Germany) according to the manufacturer's instructions. Twenty-four hours later, cells were harvested in IP/Lysis Buffer (50 mM Tris HCl pH 7.4, 150 mM NaCl, 1% NP-40, 1 mM EDTA, 20 µg/ml leupeptin, 2 µg/ml antipain, 1 µg/ml pepstatin) and incubated on a rotator at 4°C for 30 min. The samples were centrifuged at 14,000 x g and 4°C for 20 min. Lysates were either supplemented with 40 µM **NG-497** or DMSO as vehicle control. One mg of each lysate was incubated with 40 µl of Anti-FLAG M2 Affinity Gel (#A2220, Sigma-Aldrich, USA) on a rotator at 4°C for 3 h. Beads were washed three times with 500 µl IP/Lysis Buffer. Bound proteins were eluted by boiling beads in 2 x SDS sample buffer (0.1 M Tris HCl pH 6.8, 5% 2-mercaptoethanol, 4% sodium dodecyl sulfate, 20% glycerin, bromophenol blue) at 98°C for 10 min. For detection of protein expression, anti-Xpress (Thermo Fisher Scientific #R910-25), anti-mouse-HRP Trueblot (Rockland #18-8817-33), and anti-FLAG HRP (MiliporeSigma #A8592) antibodies were used.

Analysis of toxicity in HepG2 hepatocytes. For LDH-based viability assays, HepG2 cells were seeded in 96-well plates in DMEM (Gibco) containing 100 IU/ml penicillin, 100 IU/ml streptomycin (MilliporeSigma), and 3% heat-inactivated FCS. Cells were cultivated under standard conditions (37°C, 5% CO_2, 95% humidified atmosphere) and upon 80% confluency treated with different concentrations of **NG-497** dissolved in DMSO or Cisplatin dissolved in dimethylformamide (MilliporeSigma) as positive control for 24 h in fresh medium. Release of LDH into the medium was analyzed using the Cytotoxicity Detection Kit PLUS (LDH) (MilliporeSigma) according to the manufacturer's instructions. Cytotoxicity was calculated as relative LDH release as compared to fully lysed cells.

Serum stability of ATGL inhibitors. Inhibitors were incubated in 200 µl human serum for 0 and 3 h at 37°C and extracted in 1 ml MTBE/methanol (3/1, v/v) containing acetic acid/butylated hydroxytoluene (500nM). The extraction mixture was shaken at 1,400 x rpm for 1 h at RT and centrifuged for 10 min at 13,000 x rpm for phase separation. 700 µl of the upper, organic phase were collected, dried under a stream of nitrogen, and lipids resolved in 200 µl methanol/isopropanol/H_2O (6/3/1), for UPLC-qTOF analysis.

Chromatographic separation was modified after Knittelfelder et al.[2] using an AQUITY UPLC system (Waters Corporation, Milford, USA) equipped with a ZORBAX Eclipse XDB-C18- column (3 × 30 mm, 1.8 µm; Agilent Technologies, Santa Clara, USA) starting a 15 min gradient with 80% solvent A (water/methanol, 1/1, v/v) and 20% solvent B (2-propanol). Both solvents contained ammonium acetate (10 mM), formic acid (0.1 vol%) and phosphoric acid (8 µM) and the column was kept at 50°C. A SYNAPT™G1 qTOF HD mass spectrometer (Waters Corporation) equipped with an ESI source was used for analysis. The following source parameters were used: capillary temperature 100°C, desolvatization temperature: 400°C, N_2 as nebulizer gas. The capillary voltage was 2.6 kV in positive ionization mode. Data acquisition was done by the MassLynx 4.1 software (Waters Corporation).

Stability of NG-497 in HepG2 cells. 5 x 10^5 HepG2 cells were seeded in 6 well plates, next day pre-treated with 40 µM **NG-497** for 1 h, washed three times with medium, and fresh medium without **NG-497**was added to cells. Media and cells were harvested at indicated timepoints and extracted with MTBE/methanol (3:1, v/v) sovent mixture. Chromatographic separation was performed using an Agilent 1290 Infinity II UHPLC, equipped with a ZORBAX Eclipse XDB-C18- column (3 × 30 mm, 1.8 µm; Agilent Technologies, Santa Clara, USA), a flow rate of 0.3 mL/min, an injection volume of 1 µL, column temperature of 50 °C, and a mobile phase gradient: (B %) from 35 to 100% B within 9 min, where solvent (A) H_2O and (B) IPA, each containing 10 mM ammonium acetate, 7.7 µM phosphoric acid, and 1% formic acid. An Agilent 6470 triple-quadrupole mass spectrometer with an Agilent Jet Stream was used for detection. Inhibitors were analyzed in multiple reaction monitoring MRM mode, using the following transitions in positive mode; NG497; [MH]+ to 273.9 and 227.9 as quantifier and qualifier respectively. Unit resolution was used in both MS1 and MS2, the fragmentor voltage was set to 130 V, and the collision energy was set to 14 V and 26 V f for **NG-497** transitions. The ESI parameters were as follow: gas temp = 300°C; gas flow = 5 l/min; nebulizer = 30 psi; sheath gas temp = 400°C; capillary = 3500 V; nozzle voltage = 1000 V. Data processing was performed with Agilent MassHunter quantitative analysis software version 10.1 and Agilent MassHunter qualitative analysis software version 10.0. Data were normalized by calculating analyte/internal standard ratios (arbitrary unit, AU) and expressed as AU/mg or g cells protein content or AU/µl media.

Analysis of off-target inhibition. Enzymes were expressed in Expi293 cells and the enzymatic activity of lysates was determined using various substrates. Lysates of cells overexpressing LacZ were used as control. PNPLA6 and PNPLA7 enzymatic activity was analyzed using 1 mM (1-oleoyl) lysophosphatidylcholine, PNPLA8 activity using 2 mM dioleoyl phosphatidic acid, PNPLA9 activity using1 mM dioctanoyl-phosphatidylglycerol as substrate. Enzymatic assays were performed in buffer A (75 mM Tris/HCl, pH 8, 0.15 mM NaCl, 15% glycerol, 0.25% IGEPAL CA-630). Enzymatic activity of CES2, DDHD2, and HSL was analyzed using 1 mM p-nitrophenyl valerate (p-NPV) in 50 mM Tris/HCl, pH 7.5. The general lipase inhibitor *(R)*-bromoenol lactone (10 µM, R-BEL, Cayman Chemical #CAYM10006800, Ann Arbor, USA) was used as positive control for PNPLA6, PNPLA7, and PNPLA9, Orlistat (40 µM, MilliporeSigma #O4139) for PNPLA1 and PNPLA8, PMSF (10 µM, MilliporeSigma #P7626) for CES2, KLH45[3] (20 nM, MillliporeSigma #SML1998) for DDHD2, Hi 76-0079 (10 µM, NNC 0076-0000-0079 provided by Novo Nordisk, Bagsværd, Denmark) for HSL, and anti-LPL antibody[4] for post-heparin plasma.

Lysates were preincubated with 100 µM **NG-497** or control inhibitors for 10 min and incubated with respective substrates. Released free fatty acids were quantified via NEFA C kit (Fujifilm Wako Chemicals, Neuss, Germany) and release of p-nitrophenol was measured at 405 nm. Measurement of ω-*O*-acylceramide synthase activity of PNPLA1 was performed as previously described[6]. In brief, HEK293T cells were transfected with a vector expressing a multicistronic mRNA transcript encoding ELOVL4, FA ω-hydroxylase CYP4F22, and CERS3 promoting the formation of the ω-*O*-acylceramide precursor ω-hydroxy ceramide. Cells were co-transfected either with PNPLA1 and LacZ as a control or with PNPLA1 and CGI-58. The presence of CGI-58 strongly promotes the PNPLA1-dependent formation of ω-*O*-acylceramides[6]. Cellular ω-*O*-acylceramide formation was monitored using [1-^{14}C]linoleic acid as tracer. Cellular lipids were extracted and separated by TLC, and radiolabeled lipids were detected by spraying a fluorographic reagent containing scintillation cocktail on plates followed by exposure to a light-sensitive film. The activity of purified human pancreatic lipase (MilliporeSigma #SRE0028) was determined as previously described[7] in the presence or absence of **NG-497** (100µM) or Orlistat (20 µM). Circulating TG hydrolase activity was determined from post-heparin plasma using radiolabeled triolein and Intralipid (Fresenius Kabi, Bad Homburg, Germany) as substrate as previously described[5].

Lipidomic analysis. HepG2 cells were treated for 3 hours with 40 µM **NG-497** or DMSO as control. Subsequently, cells were washed three times with PBS, harvested, and extracted with 700 µl MTBE:MeOH (10:3, v:v) for 1 hour at 4 °C on a Thermomix shaker at 1,400 rpm. 200 µl H_2O were added, shaken for another 20 min, centrifuged for 10 min at 20,000 g for phase separation. 500 µl of the organic phase was transferred and evaporated under a stream of nitrogen. Lipids were resolved in 200 µl methanol/isopropanol/H_2O (14/5/2), for UHPLC-qTOF analysis. The aqueous phase was dried, the protein pellet incubated overnight with 400 µl NaOH/SDS (0.3 M/0.1%) and total protein content analyzed by the BCA protein assay (Thermo Fisher Scientific) according to the manufacturer's instructions.

Lipid samples were separated on an Agilent 1290 Infinite II UHPLC with gradient elution over a Zorbax Extend-C18 reversed phase column (2.1 x 50 mm, 1.8µm) at 50°C. Eluents of H_2O (A) and IPA (B), each containing 10 mM ammonium acetate, 7.7 µM phosphoric acid, and 1% formic acid, were used for sample elution. Baseline conditions were 60% A for 1.5 min, followed by three serial changes to 40% A within 1.5 minutes, to 12% A within 9 minutes, and within 2.5 minutes to 0% A, and held for 0.75 minutes, followed by an equilibration period of 0.5 minutes at 60% A. Elution flow was 400 µl/min and was vaporized and ionized in a Dual AJS ESI source with the following conditions: 300 °C gas temperature with a flow of 5 l/min drying gas, 30 psi nebulizer pressure, 400/350 °C (pos/neg mode) sheath gas temperature with a flow of 12 l/min, and a ionization voltage of 3500 and in negative mode: 300 °C gas temperature with a flow of 5 l/min drying gas, 30 psi nebulizer pressure, 350 °C sheath gas temperature with a flow of 12 l/min, and a ionization voltage of 3500. Samples were analyzed on an Agilent 6560 Ion Mobility Q-ToF mass spectrometer in Qtof-only mode over a scan range of 100 to 1700 m/z in negative mode and 150 to 1700 m/z in positive mode with automatic MS/MS generation. All samples were analyzed in triplicates using iterative acquisition mode to increase the number of MS/MS spectra for better annotation.

Raw data were first processed with the MassHunter lipid annotator from Agilent to identify lipids and generate a target list which was then used in the Lipid data analyzer 2.8.1[8] for peak picking and integration. Peak areas and lipid labels were manually curated. Data were normalized to internal standards and total protein content of samples. For data analysis R 4.1.2 and Rstudio 2021.09.1/372 was used with the dplyr package[9], figures were done with ggplot 2 package[10], and the lipidr workflow was used for multivariate analysis.[11]

Lipolysis in human primary and SGBS adipocytes. Human primary subcutaneous and omental preadipocytes (ZenBio, Durham, USA) were seeded in 96-well plates and differentiated according to the manufacturer's protocol. SGBS preadipocytes (Novo Department of Pediatrics and Adolescent Medicine, University of Ulm, Germany) were maintained in SGBS medium (DMEM/Nutrient Mix F12 (GIBCO, 1/1, v/v) with 8 µg/ml biotin (MilliporeSigma), 4 µg/ml pantothenic acid (MilliporeSigma), 100 IU/ml penicillin, and 100 IU/ml streptomycin (MilliporeSigma)) supplemented with 10% FCS, at 37°C in 5% CO_2, 95% humidified atmosphere. SGBS cells were seeded at 5,000 cells per well in 48-well plates. Upon confluency, differentiation was induced using SGBS medium supplemented with 0.01 mg/ml transferrin (MilliporeSigma), 1 mM cortisol (MilliporeSigma), 200 pM triiodothyronine (MilliporeSigma), 20 nM human insulin (MilliporeSigma), 0.25 mM dexamethasone (MilliporeSigma), 500 mM 3-isobutyl-1-methylxanthine (MilliporeSigma), and 2 mM rosiglitazone (Axxora, San Diego, CA). On day 4 of differentiation, the medium was replaced with an identical differentiation medium. On day 8 and 11 of differentiation, the medium was replaced by SGBS medium containing 0.01 mg/ml transferrin, 1 mM cortisol, 200 pM triiodothyronine, and 20 nM human insulin. SGBS cells were used on day 14.

For analysis of FA release, cells were preincubated with **NG-497**, Hi 76-0079, or DMSO as control for 1 h. Subsequently, the medium was replaced by DMEM with 2% FA-free BSA containing inhibitors as indicated. The release of FA in the medium was determined using commercial kits (NEFA C kit, Fujifilm Wako Chemicals). Glycerol release was analyzed using the Free Glycerol Reagent (Merck, Kenilworth, USA). Protein concentrations were determined using BCA reagent (Pierce) after extracting total lipids with hexane/isopropanol (3/2, v/v) and lysing the cells with 0.3 M NaOH/0.1% SDS. For analysis of cellular DAG content, lipid extracts were dried under a stream of nitrogen, redissolved in chloroform, and analyzed via TLC using hexane/diethyl ether/acetic acid (70/29/1, v/v/v) as mobile phase.

For lipolysis experiments with radiolabeled fatty acids, human subcutaneous adipocytes were prelabeled with 1 µCi [9,10-3H] oleic acid per well for 12 h in adipocyte maintenance medium (AM-1, ZenBio, Durham, USA). Under the applied conditions, ~ 30% of the label was incorporated into cellular lipids. Subsequently, cells were washed, preincubated with **NG-497**, and the release of radioactivity into the medium was determined in the presence or absence of Forskolin. Radioactivity in media was detected by liquid scintillation using Rotiszint eco plus (Roth) and a Micro beta 2 liquid scintillation analyzer (PerkinElmer). Cell-associated radioactivity was determined after lysis of cells with 0.3 M NaOH/0.1% SDS.

CRISPR/Cas9-mediated *ATGL* knockout in human HepG2 hepatocytes. To generate *ATGL* knockout HepG2 cells, three different gRNAs (gRNA_v1 (targeted exon 1): ACGTGGAACATCTCGTTCGC, gRNA_v2 (targeted exon 2): CTTCTCGCGGGGAAACATCG, gRNA_v3 (targeted exon 9): TGGCGCGCATCACCAGGTAC) targeting the human *PNPLA2* gene were cloned into the *Bbs*I site of pSpCas9(BB)-2A-Puro V2.0 (Addgene plasmid #62988). Correct insertion was verified by Sanger sequencing. The resulting constructs were transfected into HepG2 cells using the Turbofect Reagent (#R0531, Thermo Fisher Scientific) according to the manufacturer's instructions. Cells transfected with empty pSpCas9(BB)-2A-Puro V2.0 served as negative control. 24 h after transfection, puromycin was added at a concentration of 4 µg/ml to eliminate non-transfected cells. 48 h later, the remaining cells were singularized in 96-well plates by seeding 1 cell/well. After clonal expansion for three weeks, the resulting cell lines were screened for ATGL deletion by immunoblotting using an ATGL-specific antibody (#2138S, CST). gRNA_v1 construct was chosen for further analysis.

Determination of cellular acylglycerol content. Cells were harvested in buffer A, total lipids extracted with chloroform/methanol (2/1, v/v) for 1 h at RT on a top-over wheel, and phase separation was achieved by centrifugation at 2,000 x g for 10min at RT. The organic phase was collected, dried under a stream of nitrogen, and dissolved in 2% Triton X-100 by sonication. Acylglycerol levels were determined using TG Infinity reagent (Thermo Fisher Scientific) and glycerol as standard.

Analysis of cellular respiration. 1.5×10^4 HepG2 cells per well were seeded in Seahorse XF96 plates (Agilent Technologies) in DMEM containing 10% FCS, 100 IU/ml penicillin, and 100 IU/ml streptomycin, and cells were cultured overnight under standard conditions (37°C, 5% CO_2, 95% humidified atmosphere). Next day, cells were washed with 1x PBS and adjusted in Seahorse growth medium (DMEM 5030, 2 mM Glutamax, 25 mM glucose or 10 mM galactose, 10% FCS, 100 IU/ml penicillin, and 100 IU/ml streptomycin, pH 7.4) overnight. For assessing the extracellular acidification rate (ECAR) and oxygen consumption rate (OCR), cells were washed with 1x PBS and incubated for 1 h in Seahorse growth medium containing DMSO as vehicle control (0.1% final concentration) or 40 µM **NG-497**. Subsequently, the medium was replaced with Seahorse XF medium (DMEM 5030, 2 mM Glutamax, 10 mM galactose or 25 mM glucose, pH 7.4), equilibrated at 37°C in a non-CO_2 incubator for 1 h following manufacturer's instructions. Port A, B, C of the Seahorse XF96 plates were loaded with Oligomycin (1 µM final concentration), Carbonyl cyanide-p-trifluoromethoxyphenylhydrazone (FCCP) (1 µM), Antimycin/Rotenone (1 µM) prepared in Seahorse XF medium, respectively. Immediately after measurement in a SeaHorse XFe96 Analyzer (Agilent Technologies), cells were washed with 1x PBS and lysed in 0.3 M NaOH/0.1% SDS. Protein content was determined using the BCA reagent (Pierce) and used for OCR normalization. Respiratory parameters were calculated as follows: Non-mitochondrial oxygen consumption (NMOC) as minimum rate measured after Antimycin/Rotenone injection. Basal respiration (BR) as last rate measured bevor Oligomycin injection minus NMOC. Maximal respiration (MR) as maximal rate after FCCP injection minus NMOC. Proton leak (PL) as minimum rate after Oligomycin injection minus NMOC. Spare respiratory capacity (SRC) as MR minus BR. ATP production (ATPP) as BR minus minimal rate after Oligomycin injection.

3D homology modelling of ATGL. Homology models of mouse and human ATGL (residues 1-288) were generated using the I-TASSER server[12]. Homology modelling of ATGL is challenging since available templates (PlpD, PDB-ID: 5FQU[13]; VipD, PDB-ID: 4AKF[14]; patatin, PDB-ID: 1OXW[15]; iPLA2beta, PDB-ID: 6AUN[16]; GIVD Cytosolic Phospholipase A2, PDB-ID: 5IXC[17]; ExoU; PDB-ID: 4QMK[18]) have sequence identities of only 12-20% even when restricting the query sequences to 288 residues. All calculated models showed the same overall fold within the residues of the patatin-domain, with markedly decreasing accuracy in regions C-terminal of residue 180. Accordingly, the all-atom RMSD (ca. 4000 atoms) of the 4 best aligned human and mouse models are in the range of 2.6 Å (4 models). After 5 cycles of iterative refinement and elimination of atoms with per-atom deviations over two standard deviations from the mean deviation, the alignment scores of the remaining atoms (ca. 200-260 atoms) are in the range of 0.9 Å (0.6Å for human ATGL models). These best-aligned residues cover the atoms of the identified inhibitor binding pockets in all models providing good confidence in our model in this region. The representative models of human and mouse ATGL shown in this work have an RMSD of 0.5 Å (202 atoms aligned). Nevertheless, side chain placement varies amongst the models. Therefore, we give only a qualitative description of the overall shape of the identified binding pockets. We used PyMOL (The PyMOL Molecular Graphics System 2.4.1, Schrödinger, New York, NY) for the analysis of homology models and generation of structure-related information.

Statistical Analysis. Figures were prepared using GraphPad Prism 8 (GraphPad Software, San Diego, USA). Datasets are presented as means ± S.D. Statistical significance between two groups was determined by Student's unpaired, two-tailed *t*-test (*,

$p < 0.05$; **, $p < 0.01$; and ***, $p < 0.001$) or ANOVA followed by Bonferroni post hoc test for multiple comparison ($^{\#}$, $p < 0.05$; $^{\#\#}$, $p < 0.01$; and $^{\#\#\#}$, $p < 0.001$).

Synthesis and full spectroscopic characterization of inhibitors

General information

Reactions were carried out under air, unless indicated otherwise. For inert reactions, standard Schlenk techniques under an inert atmosphere of N_2 or Ar and anhydrous solvents were used. High pressure hydrogenation experiments were performed using the H-Cube^{TM} continuous hydrogenation unit (HC-2.SS) from Thales Nanotechnology Inc. running with a Knauer Smartline pump 100 and equipped with a 10 mL ceramic pump head. As hydrogenation catalyst 10 % Pd/C catalyst cartridges were used (Thales Nanotechnology inc., THS01111, 10 % Pd/C CatCartTM). Chemicals were purchased mainly from the companies ABCR, ACROS Organics, Alfa Aesar, Sigma Aldrich or TCI and were used without further purification, unless stated otherwise. For inert reactions, solvents were stored under an argon atmosphere, and stored over molecular sieves (4Å molecular sieves were used for CH_2Cl_2, 1,4-dioxane, DME, DMF, DMSO, Et_3N, pyridine, and THF. 3Å molecular sieves were used for ACN and EtOH). The following solvents were additionally dried and distilled under an argon atmosphere: $CH_2Cl_2(CaH_2)$, Et_3N (Na), EtOH (Na), THF (CaH_2). ACN for inert reactions was passed through an aluminium oxide column (solvent purification system: Puresolv^{TM} from Innovative Technology Inc.) under inert conditions.

Spectroscopic characterization

Specific rotation was measured at 20 °C with a wavelength of 589 nm with a Perkin Elmer Polarimeter 341. The described nuclear resonance spectra were acquired with the following instruments: Bruker AVANCE III with Autosampler: 300.36 MHz 1H-NMR, 75.53 MHz 13C-NMR; Varian Unity Inova: 499.91 MHz 1H-NMR, 125.69 MHz 13C-NMR, 470.35 MHz 19F-NMR. Chemical shifts δ [ppm] are referenced to residual protonated solvent signals as internal standard: CDCl3: δ= 7.26 ppm (1H), 77.16 ppm (13C), DMSO-d6: δ= 2.50 ppm (1H), 39.52 ppm (13C), and MeOD-d4: δ= 3.31 ppm (1H), 49.00 ppm (13C). Signal multiplicities are abbreviated as bs (broad singlet), d (dublet), dd (doublet of doublet), dq (doublet of quadruplet), dt (doublet of triplet), hept (heptett), m (multiplet), s (singlet), t (triplet), and q (quadruplet). The deuterated solvent, the chemical shifts δ in ppm (parts per million), and the coupling constants J in Hertz (Hz) are given. Deuterated solvents for nuclear resonance spectroscopy were purchased from Euriso top® (CDCl3, MeOD-d4) and Aldrich® (DMSO-d6). Data analysis was performed using the software "MestreNova". An automatic phase correction as well as an automated baseline correction (Whittaker Smoother) were performed for several spectra.

Chromatography

Analytical thin layer chromatography (TLC) was performed on Merck silica gel 60 F254 plates and spots were visualized by UV-light (λ = 254 and/or 366 nm), or by treatment with $KMnO_4$ solution (3.0 g $KMnO_4$ and 20.0 g K_2CO_3 dissolved in 300 mL of a 5 % NaOH solution). Column chromatography was performed using silica gel 60 Å (0.04-0.063 mm particle size) from Macherey-Nagel. High Resolution Mass Spectrometry (HRMS): TOF MS EI was performed on a Waters GCT premier micromass with an Electron Impact Ionization (EI)-source (70 eV) and samples were injected via direct insertion (DI). Melting points were determined on a Mel Temp® melting point apparatus (Electrothermal). Purifications via preparative HPLC were performed on a Dionex UltiMate 3000. The separation was carried out using a C-18 reversed-phase column of the type "Nucleodur® 100-5" by Macherey-Nagel at 30 °C, and detection was accomplished at a wavelength of λ = 210 nm. Three different methods were used: "method A": 0-3 min 98 % of a 0.01 % aqueous formic acid solution and 2 % CH_3CN, 3-15 min linear to 100 % CH_3CN, 15-18 min 100 % CH_3CN with a flow of 15 $mLmin^{-1}$; "method B": 0-3 min 98 % of H_2O and 2 % CH_3CN, 3-15 min linear to 100 % CH_3CN, 15-18 min 100 % CH_3CN with a flow of 15 $mLmin^{-1}$; "method C": 0-2 min 90 % of H_2O and 10 % CH_3CN, 2-12 min linear to 100 % CH_3CN, 12-14 min 100 % CH_3CN with a flow of 15 $mLmin^{-1}$.

General procedures

Equivalents of reagents and catalysts may vary by +/- 5 % or +/- 1 mol% compared to the given values.

1.1 General procedure Suzuki coupling SC1

A Schlenk tube was dried under vacuum and charged with 1.0 eq halogenated substrate, 1.1 eq boronic acid, 5 mol% $PdCl_2$(dppf), 2.1 eq CsF, and anhydrous DME (~5 mL/100 mg halogenated substrate). The mixture was degassed via three cycles of vacuum/inert gas and was stirred at 80 °C (oil-bath) overnight, after which time the reaction mixture was cooled to rt and optionally filtered through a pad of silica gel. Subsequently, the solvent was removed under reduced pressure and final purification via column chromatography yielded the pure product. Reaction control was performed via TLC analysis and/or GC-MS analysis.

1.2 General procedure esterification ES1

A Schlenk tube was dried under vacuum and charged with 1.0 eq of the carboxylic acid substrate, anhydrous THF (~2 mL/100 mg carboxylic acid substrate), and 1.5 eq of the corresponding alcohol. Subsequently, 1.1 eq EDC*HCl and 0.15 eq DMAP were added at 0 °C (ice-bath) and the mixture was stirred at rt overnight. Subsequently, the mixture was filtered when necessary and the solvent was removed under reduced pressure and final purification via column chromatography yielded the pure product. Reaction control was performed via TLC analysis and/or GC-MS analysis.

1.3 General procedure esterification ES2

A Schlenk tube was dried under vacuum and charged with 1.0 eq of the carboxylic acid substrate, anhydrous CH_2Cl_2, 3.0 eq EDC*HCl, 0.3 eq DMAP, and 3.1 eq of the corresponding alcohol. The mixture was stirred at rt overnight or over the weekend, the solvent was removed under reduced pressure, and final purification via column chromatography yielded the pure product. Reaction control was performed via TLC analysis.

1.4 General procedure saponification SA1

A Schlenk tube was charged with the ester substrate and ~10-20 mL MeOH/mmol substrate. Subsequently, 2.0-2.1 eq of a 2 M aqueous NaOH solution were added and the mixture was stirred overnight at 80-100 °C (oil-bath). The solvent was removed under reduced pressure and H_2O was added. The aqueous layer was optionally washed with CH_2Cl_2. Using 37m% HCl, the aqueous layer was acidified to pH=1 and extracted several times with EtOAc. Subsequently, the combined organic layers were dried over Na_2SO_4 or $MgSO_4$, filtered, and the solvent was removed under reduced pressure to give the pure product. Reaction control was performed via TLC analysis.

Ethyl 4`-ethoxybiphenyl-3-carboxylate (1)

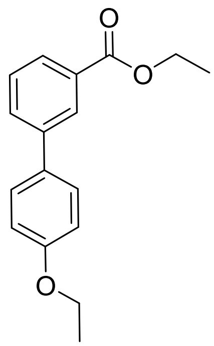

A Schlenk tube was dried under vacuum, filled with nitrogen and charged consecutively with 100 mg (70.0 µL, 437 µmol, 1.00 eq) ethyl-3-bromobenzoate, 72.5 mg (437 µmol, 1.00 eq) 4-ethoxyphenylboronic acid, 140 mg (918 µmol, 2.10 eq) CsF, 17.8 mg (22 µmol, 0.05 eq) $PdCl_2$(dppf)*DCM and 12 mL anhydrous DME. The suspension was degassed by vacuum/N_2 cycles and stirred at 80 °C. As GC-MS analysis indicated after 7 h full conversion of the starting material, the reaction mixture was cooled to rt and filtered through a pad of celite, which was rinsed with EtOAc. The solvent from the filtrate was removed under reduced pressure and purification by column chromatography (CH/EtOAc 15:1, size: 12.5 x 2.0 cm, 15 g silica gel) yielded 109.5 mg of a colorless solid.

$C_{17}H_{18}O_3$ [270.3]
yield: 109.5 mg (93 %), colorless solid
R_f (CH/ EtOAc 15:1): 0.40
^{1}H-NMR (300 MHz, $CDCl_3$): δ (ppm) = 8.25 (t, ^4J = 1.5 Hz, 1H, Ar-H), 7.98 (d, ^3J = 7.8 Hz, 1H, Ar-H), 7.74 (d, ^3J = 7.8 Hz, 1H, Ar-H), 7.56 (d, ^3J = 9.0 Hz, 2H, Ar-H), 7.48 (t, ^3J = 7.8 Hz, 1H, Ar-H), 6.98 (d, ^3J = 9.0 Hz, 2H, Ar-H), 4.41 (q, ^3J = 7.2 Hz, 2H, CH_2), 4.09 (q, ^3J = 6.9 Hz, 2H, CH_2), 1.47-1.39 (m, 6H, 2 CH_3).
^{13}C-NMR (75.5 MHz, $CDCl_3$): δ (ppm) = 166.6 (C=O), 158.8 (C_q), 141.0 (C_q), 132.5 (C_q), 131.0 (C_q), 130.9 (CH_{Ar}), 128.7 (CH_{Ar}), 128.2 (2 CH_{Ar}), 127.7 (CH_{Ar}), 127.6 (CH_{Ar}), 114.8 (2 CH_{Ar}), 63.8 (CH_2), 61.0 (CH_2), 14.8 (CH_3), 14.3 (CH_3).
m.p.: 46-47 °C
HRMS (EI^+): *m/z*: calcd for $C_{17}H_{18}O_3$ $[M]^+$: 270.1256; found: 270.1260.

Ethyl 6-bromopicolinate

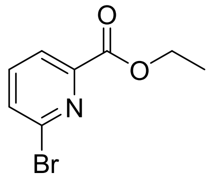

A 8 mL Schlenk tube was flushed with nitrogen and charged with 250 mg (1.24 mmol, 1.00 eq) 6-bromopicolinic acid suspended in 1.0 mL EtOH. After adding 200 µL (3.71 mmol, 3.00 eq) conc. H_2SO_4 the suspension was heated under reflux for 3 h. TLC analysis (CH/EtOAc 3:1) indicated full conversion of the starting material. The mixture was cooled to RT and and quenched by the addition of 5 mL saturated aqueous $NaHCO_3$ solution and then extracted with EtOAc (2 x 10 mL). The combined organic layers were dried over $MgSO_4$ and the solvent was removed under reduced pressure. Drying in high vacuum yielded the pure product which was used in the next step without further purification.

$C_8H_8O_2NBr$ [229.9]
yield: 249.5 mg (88 %), beige solid
R_f(CH/ EtOAc 3:1): 0.44
^{1}H-NMR (300 MHz, $CDCl_3$): δ (ppm) = 8.07 (dd, ^3J = 6.6 Hz, ^4J = 1.8 Hz, 1H, Ar-H), 7.72-7.64 (m, 2H, Ar-H), 4.46 (q, ^3J = 7.2 Hz, 2H, CH_2), 1.42 (t, ^3J = 7.2 Hz, 3H, CH_3).
^{13}C-NMR (75.5 MHz, $CDCl_3$): δ (ppm) = 163.9 (C=O), 149.1 (C_q), 142.1 (C_q-Br), 139.1 (CH_{Ar}), 131.6 (CH_{Ar}), 123.9 (CH_{Ar}), 62.3 (CH_2), 14.2 (CH_3).
m.p.: 32 °C

Ethyl 6-(4-ethoxyphenyl)picolinate (2)

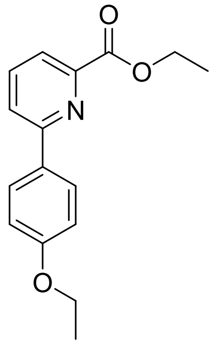

A Schlenk tube was dried under vacuum, filled with nitrogen and charged consecutively with 100 mg (435 µmol, 1.00 eq) ethyl-6-bromopicolinate, 72.2 mg (435 µmol, 1.00 eq) 4-ethoxyphenylboronic acid, 139 mg (914 µmol, 2.10 eq) CsF, 37.3 mg (45 µmol, 0.05 eq) $PdCl_2$(dppf)*DCM and 12 mL anhydrous DME. The suspension was degassed by vacuum/N_2 cycles and stirred at 80 °C. As GC-MS analysis indicated after 4.5 h full conversion of the starting material, the reaction mixture was cooled to rt and filtered through a pad of celite, which was rinsed with EtOAc. The solvent from the filtrate was removed under reduced pressure and the crude product purified via column chromatography (CH/EtOAc 3:1, size: 17.0 x 2.0 cm, 25 g silica gel) to yield 96.0 mg of a beige solid.

$C_{16}H_{17}O_3N$ [271.3]
yield: 96.0 mg (81 %), beige solid
R_f (CH/ EtOAc 3:1): 0.47
^{1}H-NMR (300 MHz, $CDCl_3$): δ (ppm) = 8.03 (d, ^3J = 9.0 Hz, 2H, Ar-H), 7.99-7.96 (m, 1H, Ar-H), 7.84-7.82 (m, 2H, Ar-H), 6.99 (d, ^3J = 9.0 Hz, 2H, Ar-H), 4.48 (q, ^3J = 7.2 Hz, 2H, CH_2), 4.09 (q, ^3J = 6.9 Hz, 2H, CH_2), 1.48-1.42 (m, 6H, 2 CH_3).
^{13}C-NMR (75.5 MHz, $CDCl_3$): δ (ppm) = 165.5 (C=O), 160.2 (C_q), 157.2 (C_q), 148.1 (C_q), 137.5 (CH_{Ar}), 130.8 (C_q), 128.5 (2 CH_{Ar}), 122.7 (CH_{Ar}), 122.5 (CH_{Ar}), 114.7 (2 CH_{Ar}), 63.5 (CH_2), 61.8 (CH_2), 14.8 (CH_3), 14.3 (CH_3).
m.p.: 103-106 °C
HRMS (EI^+): *m/z*: calcd for $C_{16}H_{17}O_3N$ $[M]^+$: 271.1208; found: 271.1198.

Methyl 6-(4-ethoxyphenyl)picolinate (NG-442)

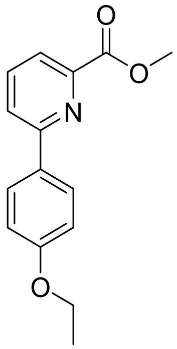

The coupling of methyl 6-bromopyridine-2-carboxylate with 4-ethoxyphenylboronic acid was performed following the general procedure SC1 with the modification that 1.0 g of the bromine substrate were dissolved in 25 mL DME.

Yield= 1.086 g yellowish solid (4.22 mmol, 91 %).
R_f= 0.34 (cyclohexane/EtOAc= 4/1; UV).
^{1}H-NMR (300 MHz, $CDCl_3$): δ (ppm) = 8.08-7.90 (m, 3 H), 7.89-7.75 (m, 2 H), 6.98 (d, *J*= 8.6 Hz, 2 H), 4.17–3.90 (m, 5 H), 1.52– 1.32 (t, *J*= 6.87 Hz, 3 H)
^{13}C-NMR,APT (76 MHz, $CDCl_3$): δ (ppm) = 166.2, 160.4, 157.5, 148.0, 137.7, 131.0, 128.6, 123.0, 122.7, 114.9, 63.7, 52.9, 14.9.
m.p.= 102-104 °C.
HRMS (EI-MS) *m/z*: for $C_{15}H_{15}NO_3$: calcd: 257.1052, found: 257.1050, Δm= 0.8 ppm.

6-(4-Ethoxyphenyl)picolinic acid (NG-482)

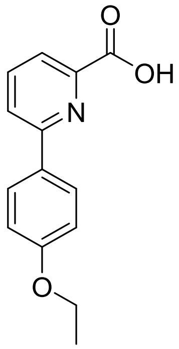

The saponification of methyl 6-(4-ethoxyphenyl)picolinate (**NG-442**) was performed following the general procedure SA1.

Yield= 470.8 mg slightly yellowish solid (1.935 mmol, 96 %)
R_f= 0.57 (CH_2Cl_2/MeOH= 9/1+some drops HOAc; UV).
^{1}H-NMR (300 MHz, $CDCl_3$): δ= 8.18-7.85 (m, 5 H), 7.04 (d, *J*= 8.8 Hz, 2 H), 4.12 (q, *J*= 7.0 Hz, 2 H), 1.47 (t, *J*= 7.0 Hz, 3 H).

Isopropyl 6-(4-ethoxyphenyl)picolinate (3)

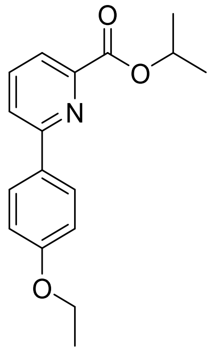

The esterification of 6-(4-ethoxyphenyl)picolinic acid (**NG-384**) with 2-propanol was performed following the general procedure ES1.

Yield= 21.3 mg slightly yellow solid (0.0747 mmol, 52 %).
R_f= 0.21 (cyclohexane/EtOAc= 10/1; $KMnO_4$).
^{1}H-NMR (300 MHz, $CDCl_3$): δ (ppm) = 8.04 (d, *J*= 8.6 Hz, 2 H), 7.98–7.90 (m, 1 H), 7.83 (d, *J*= 4.2 Hz, 2 H), 6.99 (d, *J*= 8.6 Hz, 1 H), 5.44–5.23 (m, 1 H), 4.09 (q, *J*= 6.9 Hz, 2 H), 1.55-1.30 (m, 9 H).
^{13}C-NMR,APT (76 MHz, $CDCl_3$): δ (ppm) = 165.0, 160.4, 157.3, 148.5, 137.7, 130.9, 128.7, 122.7, 122.6, 114.8, 69.5, 63.7, 22.0, 14.9.
m.p.= 96-98 °C.
HRMS (EI-MS) *m/z*: for $C_{17}H_{19}NO_3$: calcd: 285.1365, found: 285.1361, Δm= 1.4 ppm.

Ethyl 6-(4-ethoxyphenyl)-4-methylpicolinate (NG-423)

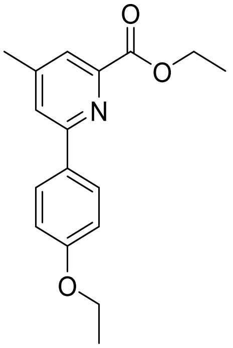

The coupling of ethyl 6-chloro-4-methylpyridine-2-carboxylate with 4-ethoxyphenylboronic acid was performed following the general procedure SC1 with the modification that 2.2 eq CsF were used and that the reaction mixture was stirred 4 times overnight. An additional crystallization was performed for purification.

Yield= 52.7 mg colorless crystals (0.185 mmol, 37 %).
R_f= 0.50 (cyclohexane/EtOAc= 3/1; UV, $KMnO_4$).
^{1}H-NMR (300 MHz, $CDCl_3$): δ (ppm) = 8.00 (d, *J*= 8.6 Hz, 2 H), 7.82 (s, 1 H), 7.65 (s, 1 H), 6.98 (d, *J* = 8.6 Hz, 2 H), 4.47 (q, *J*= 7.1 Hz, 2 H), 4.09 (q, *J*= 6.9 Hz, 2 H), 2.46 (s, 3 H), 1.45 (2xt, 6 H).
^{13}C-NMR,APT (76 MHz, $CDCl_3$): δ (ppm) = 165.8, 160.4, 157.3, 149.1, 148.0, 130.9, 128.7, 123.8, 123.8, 114.8, 63.7, 61.9, 21.4, 14.9, 14.5.
m.p.: 90-91 °C.
HRMS (EI-MS) *m/z*: for $C_{17}H_{19}NO_3$: calcd= 285.1365, found= 285.1375, Δm= 3.9 ppm.

Isopropyl 6-(4-ethoxyphenyl)-4-methylpicolinate (4)

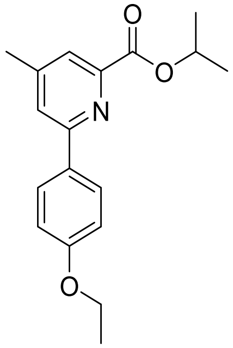

The saponification of ethyl 6-(4-ethoxyphenyl)-4-methylpicolinate (**NG-423**) was performed following the general procedure SA1. The esterification with 2-propanol was performed following the general procedure ES2 with the modification that 0.27 eq DMAP were used.

Yield= 15.0 mg colorless solid (0.0501 mmol, 47 % calc. over 2 steps).
R_f= 0.38 (cyclohexane/EtOAc= 5/1; $KMnO_4$).
^{1}H-NMR (300 MHz, $CDCl_3$): δ (ppm) = 8.02 (d, *J*= 8.7 Hz, 2 H), 7.78 (s, 1 H), 7.64 (s, 1 H), 6.97 (d, *J*= 8.7 Hz, 2 H), 5.45-5.20 (m, 1 H), 4.09 (q, *J*= 6.9 Hz, 2 H), 2.46 (s, 3 H), 1.50-1.32 (m, 9 H).
^{13}C-NMR,APT (76 MHz, $CDCl_3$): δ (ppm) = 165.3, 160.3, 157.3, 148.8, 148.5, 131.2, 128.6, 123.7, 123.4, 114.7, 69.4, 63.7, 22.1, 21.4, 14.9.
m.p.: 85-88 °C.
HRMS (EI-MS) *m/z*: for $C_{18}H_{21}NO_3$: calcd= 299.1521, found= 299.1521.

Methyl 6-(4-ethoxyphenyl)-4-methoxypicolinate (NG-470)

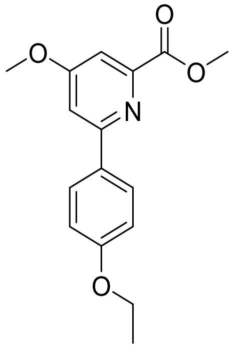

The coupling of methyl 6-chloro-4-methoxypicolinate with 4-ethoxyphenylboronic acid was performed following the general procedure SC1.

Yield= 91.0 mg colorless solid (0.317 mmol, 64 %).
R_f= 0.36 (cyclohexane/EtOAc= 3/1; UV, $KMnO_4$).
^{1}H-NMR (300 MHz, $CDCl_3$): δ (ppm) = 7.96 (d, *J*= 8.7 Hz, 2 H), 7.56 (d, *J*= 2.1 Hz, 1 H), 7.31 (d, *J*= 2.1 Hz, 1 H), 6.97 (d, *J*= 8.7 Hz, 2 H), 4.16–3.86 (m, 8 H), 1.44 (t, *J*= 7.0 Hz, 3 H).
^{13}C-NMR,APT (76 MHz, $CDCl_3$): δ (ppm) = 167.6, 165.9, 160.6, 159.0, 149.3, 130.6, 128.9, 114.8, 109.2 (2x), 63.7, 56.0, 53.1, 14.9.
m.p.: 94-103 °C.
HRMS (EI-MS) *m/z*: for $C_{16}H_{17}NO_4$: calcd= 287.1158, found= 287.1162, Δm= 1.4 ppm.

Isopropyl 6-(4-ethoxyphenyl)-4-methoxypicolinate (NG-497)

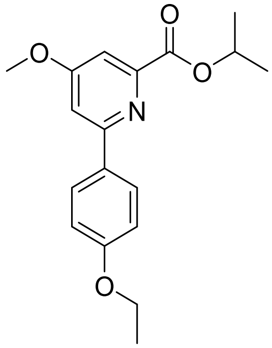

A Schlenk tube was charged with 75.0 mg (0.261 mmol) methyl 6-(4-ethoxyphenyl)-4-methoxypicolinate (**NG-470**) and 1.5 mL MeOH. Subsequently, 183 µL 2M aqueous NaOH (0.366 mmol) were added and the mixture was stirred overnight at 80 °C (oil-bath), after which time TLC analysis indicated all starting material to be consumed. The solvent was removed under reduced pressure and 5 mL H_2O were added. Using 37% HCl, the aqueous layer was acidified to pH=1 and was extracted with EtOAc (5x5 mL). Subsequently, the combined organic layers were dried over $MgSO_4$, filtered through cotton, and the solvent was removed under reduced pressure to deliver 45.8 mg of a gum-like substance.
In a round-bottom flask equipped with an Ar-inlet, were placed 36.5 mg of the crude gum-like substance, 1 mL anhydrous CH_2Cl_2, 76.8 mg (0.401 mmol) EDC*HCl, 5.1 mg (41.7 µmol) DMAP, and 32.9 µL (0.427 mmol) *i*-PrOH. The mixture was stirred at rt overnight, until which time TLC indicated all starting material to be consumed. The solvent was removed under reduced pressure and the crude product was purified via column chromatography (cyclohexane/EtOAc= 5/1) and 22.3 mg (0.0707 mmol, 34 % calc. over 2 steps) of **NG-497** were isolated as a colorless solid.

R_f= 0.29 (cyclohexane/EtOAc= 5/1; UV).
^{1}H-NMR (300 MHz, $CDCl_3$): δ (ppm) = 8.00 (d, *J*= 8.6 Hz, 2 H), 7.58-7.45 (m, 1 H), 7.35-7.25 (m, 1 H), 6.97 (d, *J*= 8.6 Hz, 2 H), 5.42–5.20 (m, 1 H), 4.09 (q, *J*= 6.9 Hz, 2 H), 3.96 (s, 3 H), 1.54–1.28 (m, 9 H).
^{13}C-NMR,APT (76 MHz, $CDCl_3$): δ (ppm) = 167.4, 164.8, 160.5, 158.8, 149.9, 130.7, 128.8, 114.7, 109.1, 108.5, 69.8, 63.7, 55.8, 22.0, 14.9.
m.p.: 79-81 °C.
HRMS (EI-MS) *m/z*: for $C_{18}H_{21}NO_4$: calcd= 315.1471, found= 315.1475, Δm= 1.3 ppm.

NMR spectra of ethyl 4`-ethoxybiphenyl-3-carboxylate (1)

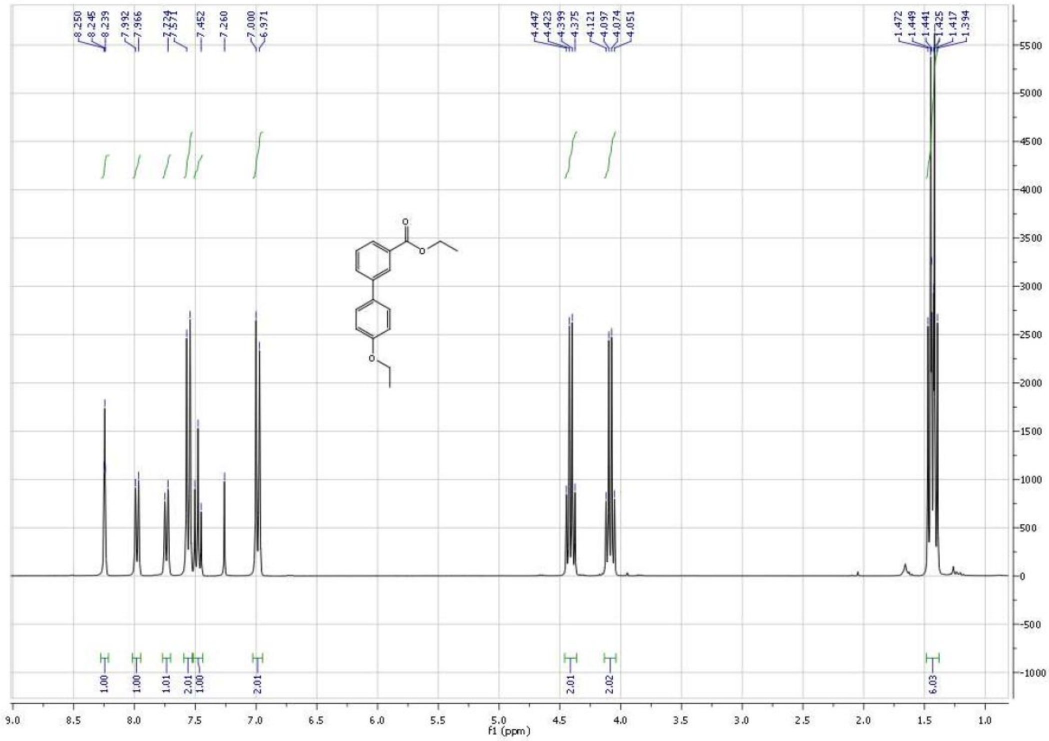

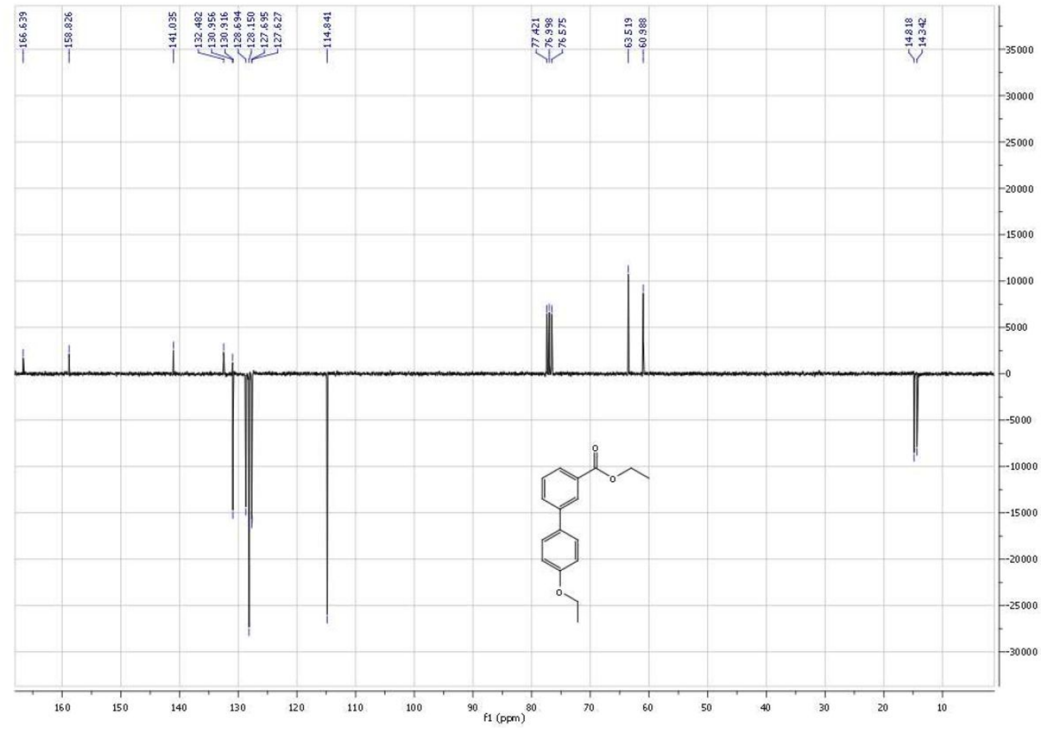

NMR spectra of ethyl 6-(4-ethoxyphenyl)picolinate (2)

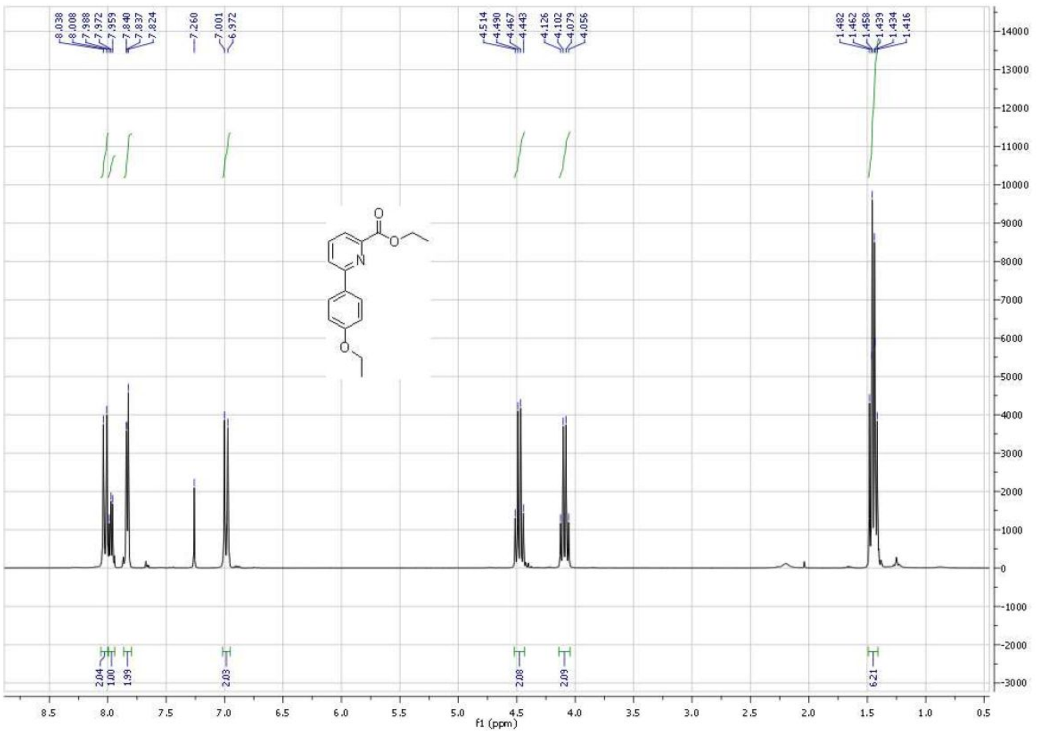

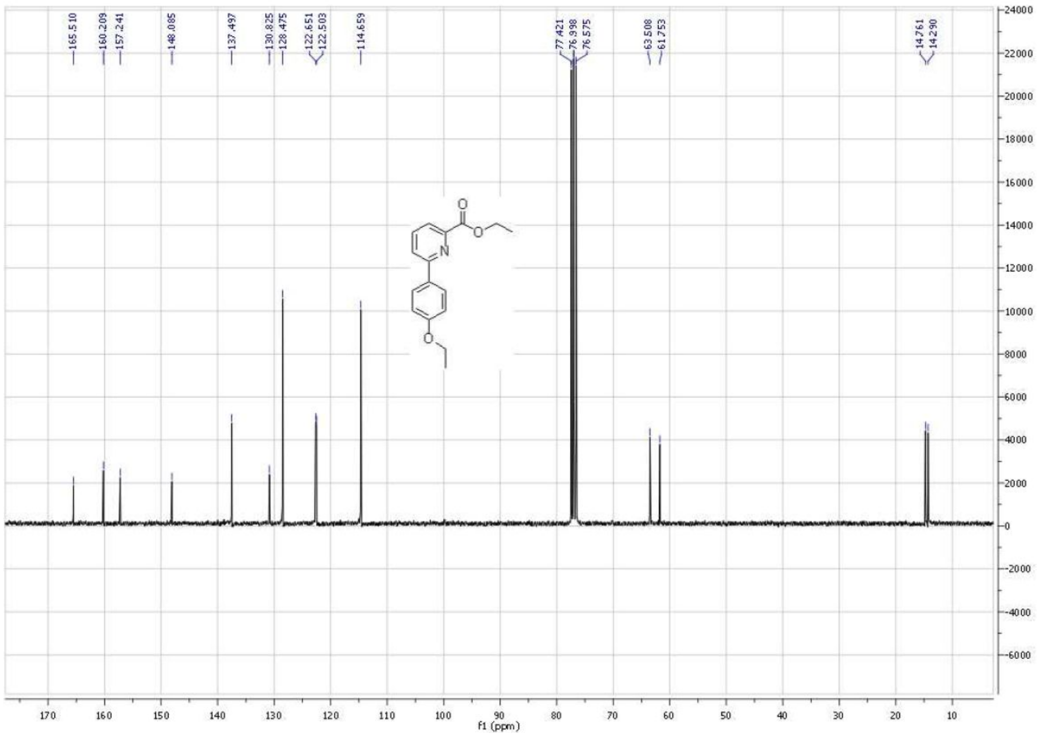

NMR spectra of ethyl 6-(4-ethoxyphenyl)picolinate (NG-442)

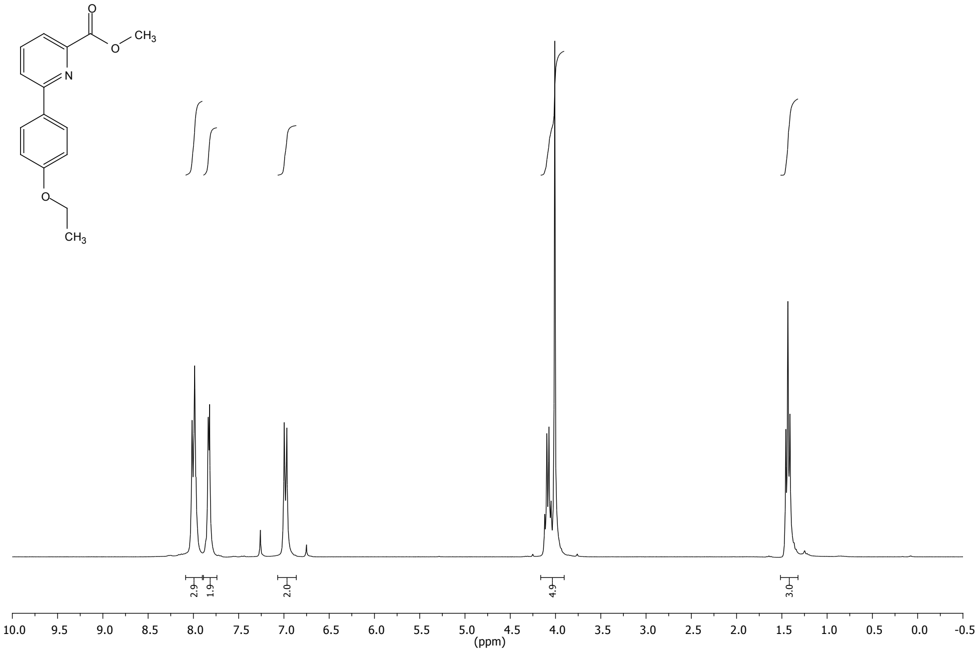

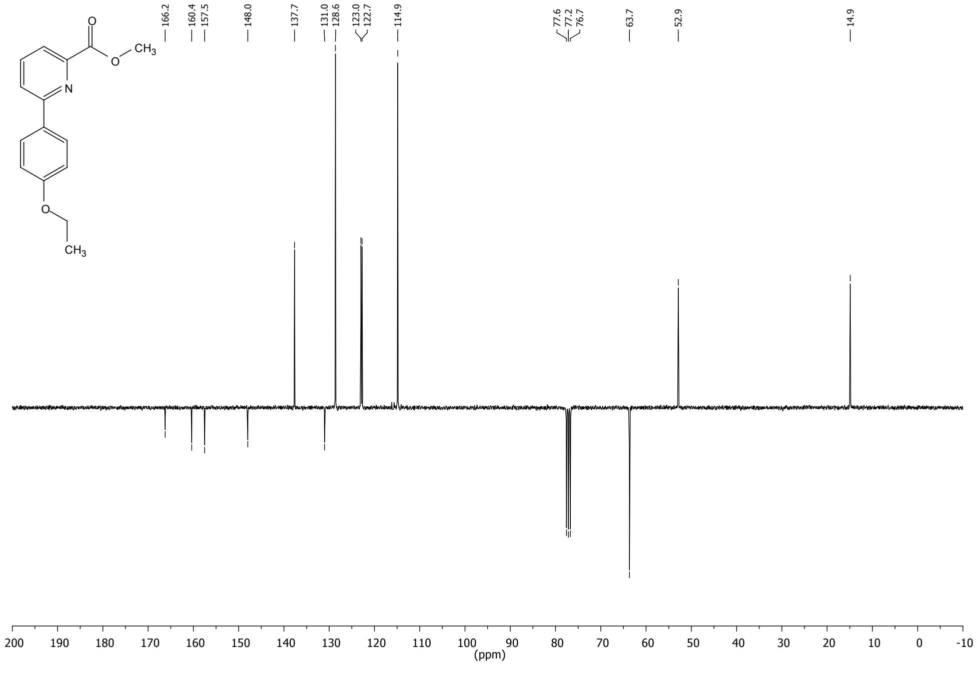

^{1}H NMR spectrum of 6-(4-Ethoxyphenyl)picolinic acid (NG-482)

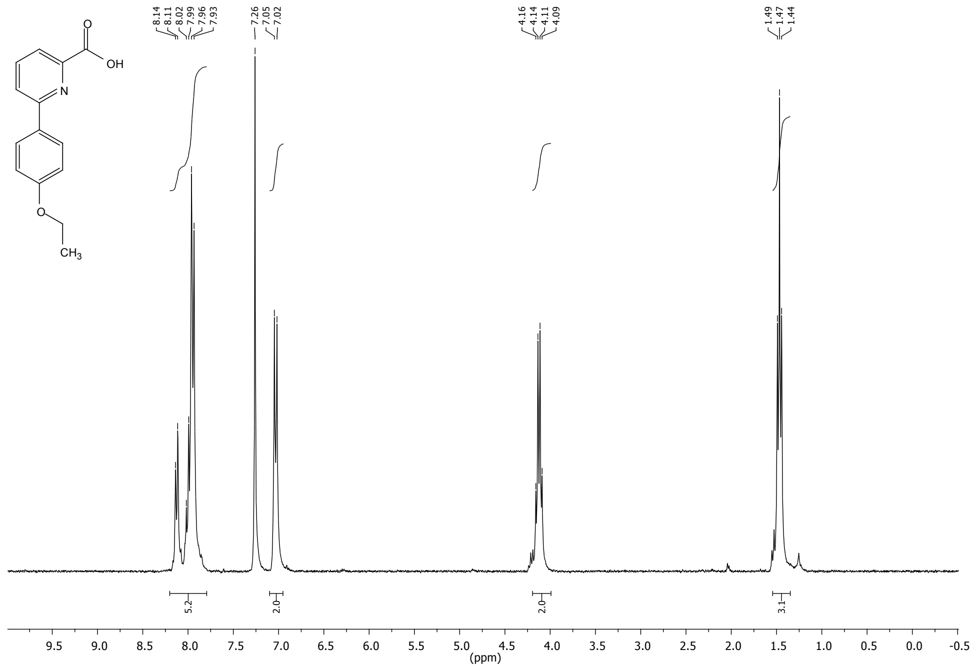

NMR spectra of isopropyl 6-(4-ethoxyphenyl)picolinate (3)

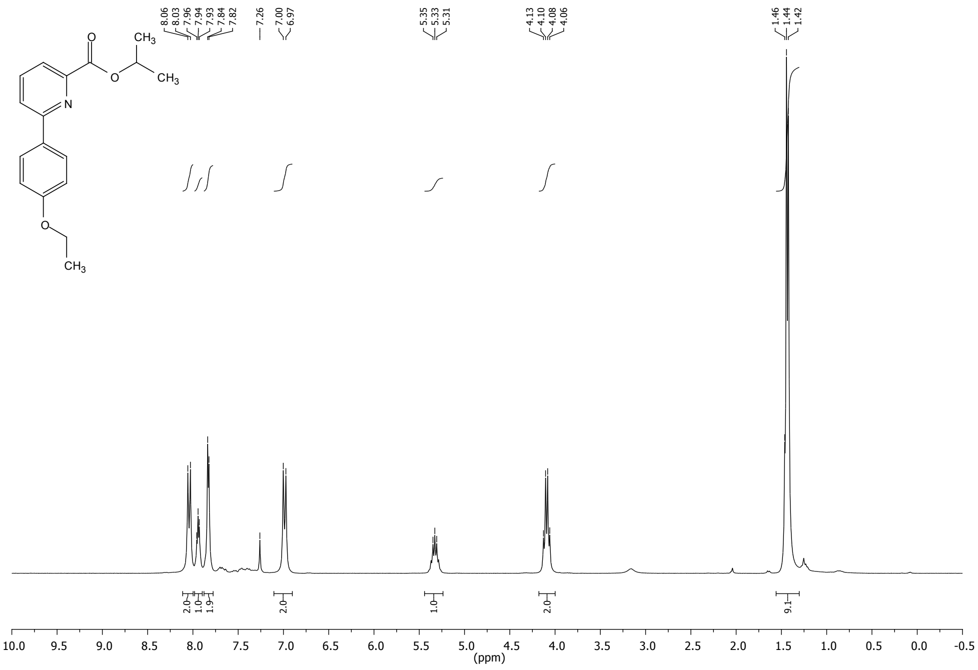

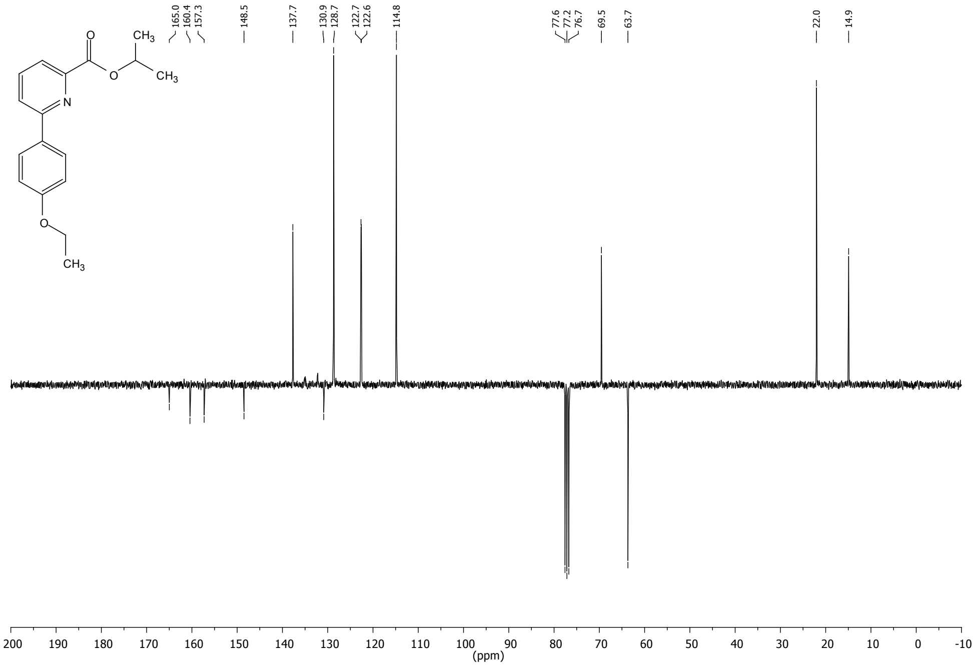

NMR spectra of ethyl 6-(4-ethoxyphenyl)-4-methylpicolinate (NG-423)

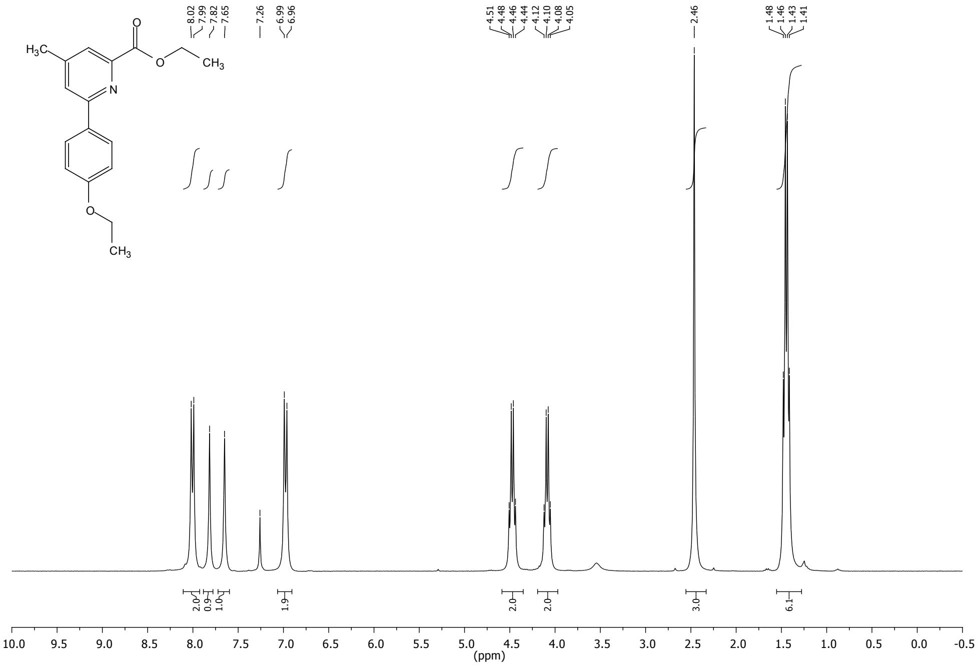

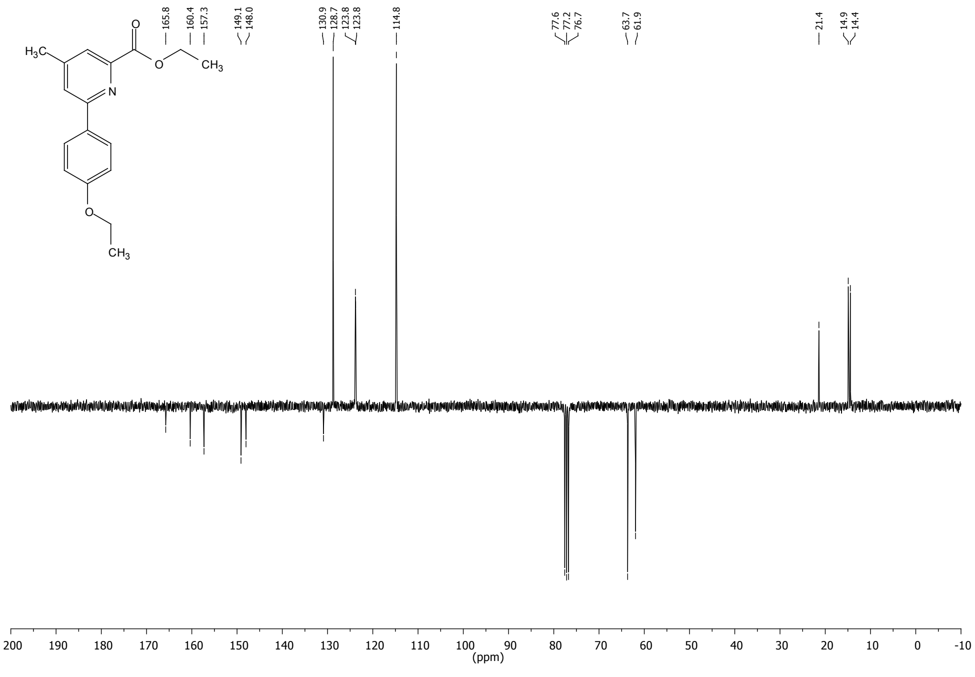

NMR spectra of isopropyl 6-(4-ethoxyphenyl)-4-methylpicolinate (4)

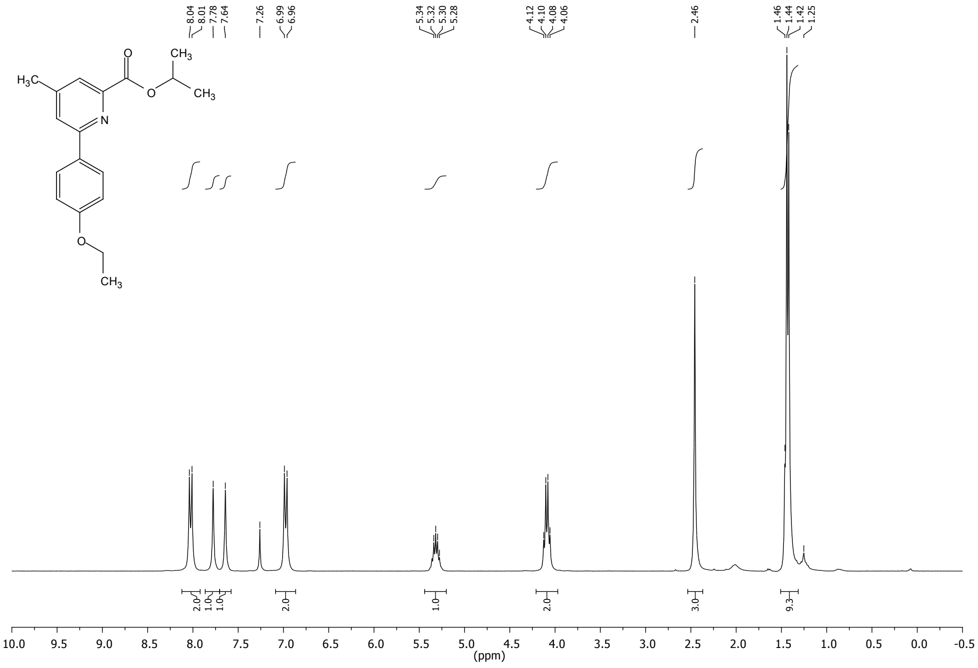

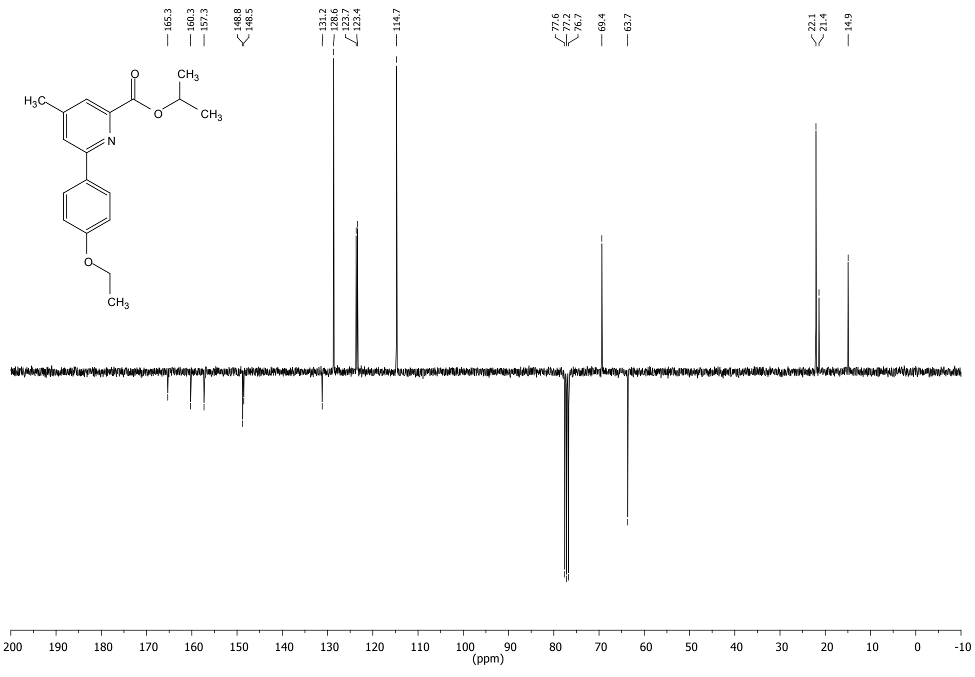

NMR spectra of methyl 6-(4-ethoxyphenyl)-4-methoxypicolinate (NG-470)

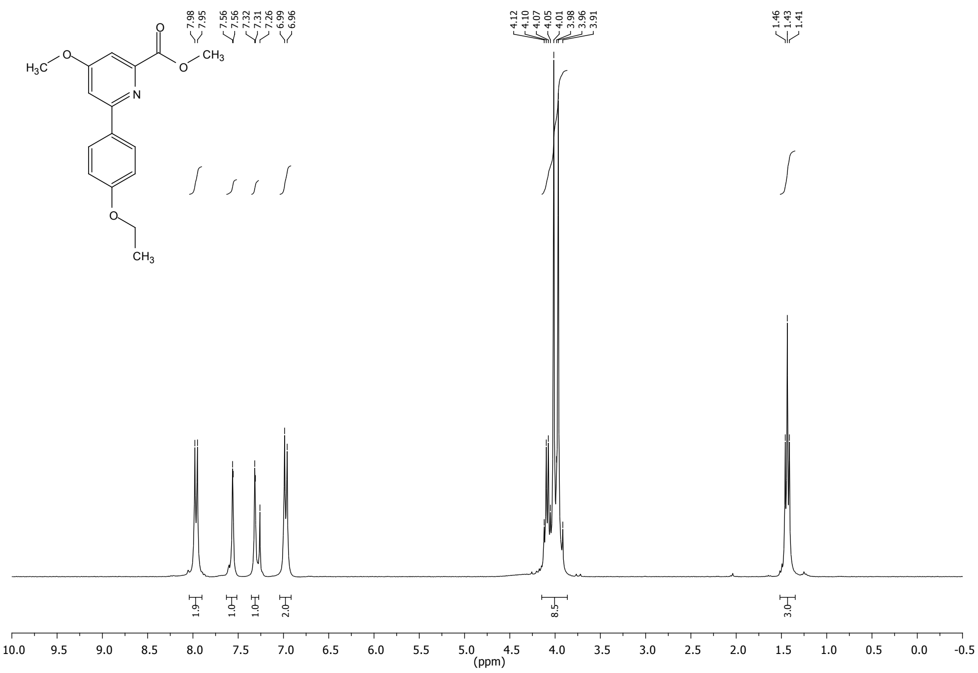

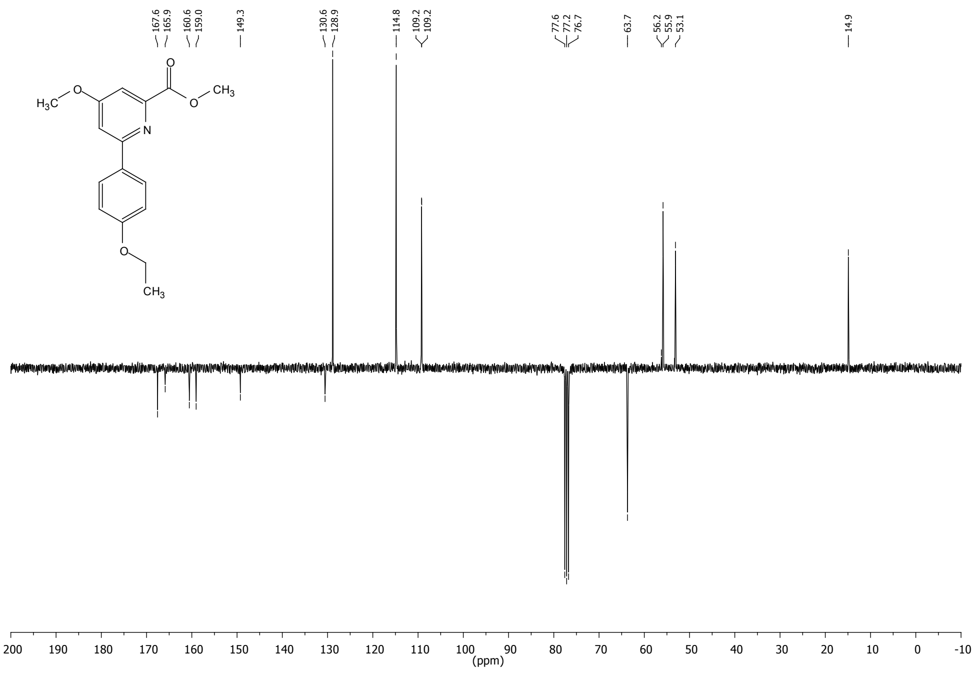

NMR spectra of isopropyl 6-(4-ethoxyphenyl)-4-methoxypicolinate (NG-497)

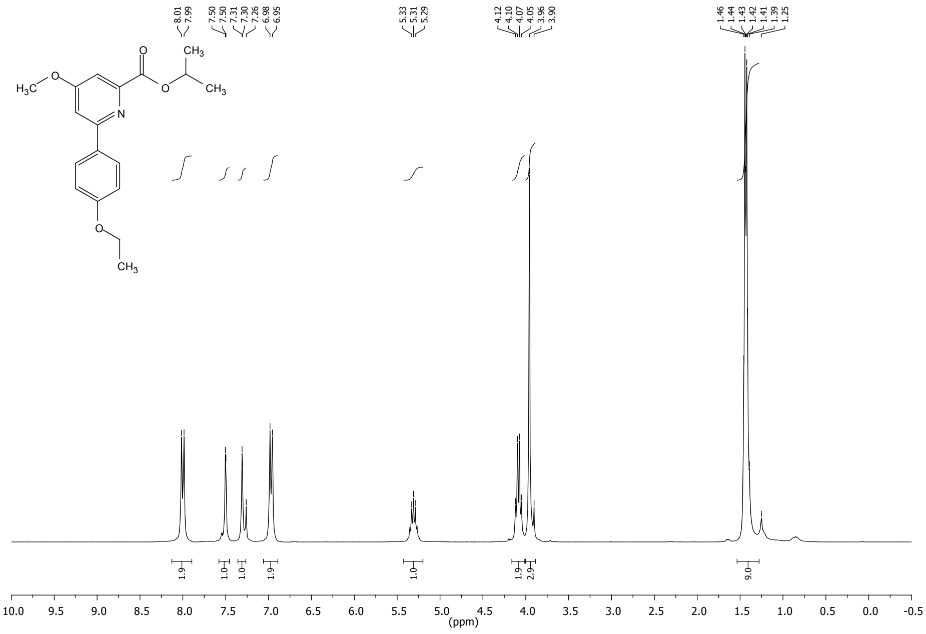

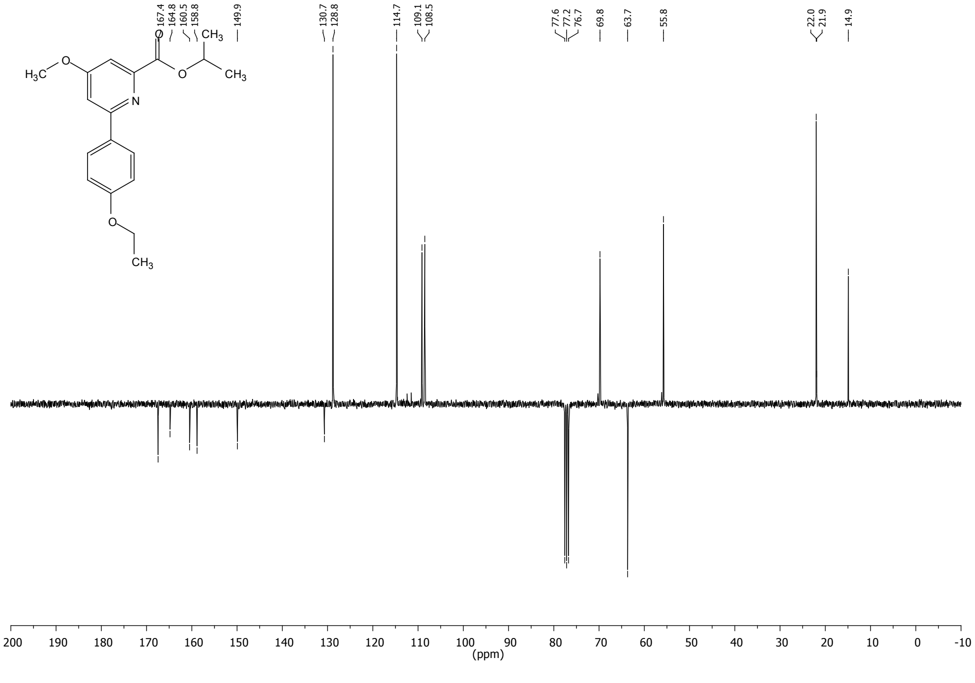

Supplemental references

(1) Hofer, P.; Boeszoermenyi, A.; Jaeger, D.; Feiler, U.; Arthanari, H.; Mayer, N.; Zehender, F.; Rechberger, G.; Oberer, M.; Zimmermann, R.; Lass, A.; Haemmerle, G.; Breinbauer, R.; Zechner, R.; Preiss-Landl, K. Fatty Acid-Binding Proteins Interact with Comparative Gene Identification-58 Linking Lipolysis with Lipid Ligand Shuttling. *J. Biol. Chem.* **2015**, *290* (30), 18438–18453. https://doi.org/10.1074/jbc.M114.628958.

(2) Knittelfelder, O. L.; Weberhofer, B. P.; Eichmann, T. O.; Kohlwein, S. D.; Rechberger, G. N. A Versatile Ultra-High Performance LC-MS Method for Lipid Profiling. *J. Chromatogr. B. Analyt. Technol. Biomed. Life Sci.* **2014**, *951–952*, 119–128. https://doi.org/10.1016/j.jchromb.2014.01.011.

(3) Inloes, J. M.; Hsu, K.-L.; Dix, M. M.; Viader, A.; Masuda, K.; Takei, T.; Wood, M. R.; Cravatt, B. F. The Hereditary Spastic Paraplegia-Related Enzyme DDHD2 Is a Principal Brain Triglyceride Lipase. *Proc. Natl. Acad. Sci. U. S. A.* **2014**, *111* (41), 14924–14929. https://doi.org/10.1073/pnas.1413706111.

(4) Liu, M. S.; Ma, Y.; Hayden, M. R.; Brunzell, J. D. Mapping of the Epitope on Lipoprotein Lipase Recognized by a Monoclonal Antibody (5D2) Which Inhibits Lipase Activity. *Biochim. Biophys. Acta - Lipids Lipid Metab.* **1992**, *1128* (1), 113–115. https://doi.org/10.1016/0005-2760(92)90264-V.

(5) Schreiber, R.; Hofer, P.; Taschler, U.; Voshol, P. J.; Rechberger, G. N.; Kotzbeck, P.; Jaeger, D.; Preiss-Landl, K.; Lord, C. C.; Brown, J. M.; Haemmerle, G.; Zimmermann, R.; Vidal-Puig, A.; Zechner, R. Hypophagia and Metabolic Adaptations in Mice with Defective ATGL-Mediated Lipolysis Cause Resistance to HFD-Induced Obesity. *Proc. Natl. Acad. Sci. U. S. A.* **2015**, *112* (45), 13850–13855. https://doi.org/10.1073/pnas.1516004112.

(6) Kien, B.; Grond, S.; Haemmerle, G.; Lass, A.; Eichmann, T. O.; Radner, F. P. W. ABHD5 Stimulates PNPLA1-Mediated -O-Acylceramide Biosynthesis Essential for a Functional Skin Permeability Barrier. *J. Lipid Res.* **2018**, *59* (12), 2360–2367. https://doi.org/10.1194/jlr.M089771.

(7) Mayer, N.; Schweiger, M.; Romauch, M.; Grabner, G. F.; Eichmann, T. O.; Fuchs, E.; Ivkovic, J.; Heier, C.; Mrak, I.; Lass, A.; Höfler, G.; Fledelius, C.; Zechner, R.; Zimmermann, R.; Breinbauer, R. Development of Small-Molecule Inhibitors Targeting Adipose Triglyceride Lipase. *Nat. Chem. Biol.* **2013**, *9*, 785–787. https://doi.org/10.1038/nchembio.1359.

(8) Hartler, J.; Triebl, A.; Ziegl, A.; Trötzmüller, M.; Rechberger, G. N.; Zeleznik, O. A.; Zierler, K. A.; Torta, F.; Cazenave-Gassiot, A.; Wenk, M. R.; Fauland, A.; Wheelock, C. E.; Armando, A. M.; Quehenberger, O.; Zhang, Q.; Wakelam, M. J. O.; Haemmerle, G.; Spener, F.; Köfeler, H. C.; Thallinger, G. G. Deciphering Lipid Structures Based on Platform-Independent Decision Rules. *Nat. Methods* **2017**, No. October. https://doi.org/10.1038/nmeth.4470.

(9) Wickman, H.; François, R.; Henry, L.; Muller, K. Dlpyr: A Grammar of Data Manipulation. *CRAN Repository*. 2021, pp 1–2. https://doi.org/https://cran.r-project.org/web/packages/dplyr/index.html.

(10) Valero-Mora, P. M. Ggplot2: Elegant Graphics for Data Analysis. *J. Stat. Softw.* **2010**, *35* (Book Review 1). https://doi.org/10.18637/jss.v035.b01.

(11) Mohamed, A.; Molendijk, J.; Hill, M. M. Lipidr: A Software Tool for Data Mining and Analysis of Lipidomics Datasets. *J. Proteome Res.* **2020**, *19* (7), 2890–2897. https://doi.org/10.1021/acs.jproteome.0c00082.

(12) Yang, J.; Yan, R.; Roy, A.; Xu, D.; Poisson, J.; Zhang, Y. The I-TASSER Suite: Protein Structure and Function Prediction. *Nat. Methods* **2014**, *12* (1), 7–8. https://doi.org/10.1038/nmeth.3213.

(13) Da Mata Madeira, P. V.; Zouhir, S.; Basso, P.; Neves, D.; Laubier, A.; Salacha, R.; Bleves, S.; Faudry, E.; Contreras-Martel, C.; Dessen, A. Structural Basis of Lipid Targeting and Destruction by the Type v Secretion System of Pseudomonas Aeruginosa. *J. Mol. Biol.* **2016**, *428* (9), 1790–1803. https://doi.org/10.1016/j.jmb.2016.03.012.

(14) Ku, B.; Lee, K.-H.; Park, W. S.; Yang, C.-S.; Ge, J.; Lee, S.-G.; Cha, S.-S.; Shao, F.; Heo, W. Do; Jung, J. U.; Oh, B.-H. VipD of Legionella Pneumophila Targets Activated Rab5 and Rab22 to Interfere with Endosomal Trafficking in Macrophages. *PLoS Pathog.* **2012**, *8* (12), e1003082. https://doi.org/10.1371/journal.ppat.1003082.

(15) Rydel, T. J.; Williams, J. M.; Krieger, E.; Moshiri, F.; Stallings, W. C.; Brown, S. M.; Pershing, J. C.; Purcell, J. P.; Alibhai, M. F. The Crystal Structure, Mutagenesis, and Activity Studies Reveal That Patatin Is a Lipid Acyl Hydrolase with a Ser-Asp Catalytic Dyad. *Biochemistry* **2003**, *42* (22), 6696–6708. https://doi.org/10.1021/bi027156r.

(16) Malley, K. R.; Koroleva, O.; Miller, I.; Sanishvili, R.; Jenkins, C. M.; Gross, R. W.; Korolev, S. The Structure of IPLA2β Reveals Dimeric Active Sites and Suggests Mechanisms of Regulation and Localization. *Nat. Commun.* **2018**, *9* (1), 1–11. https://doi.org/10.1038/s41467-018-03193-0.

(17) Wang, H.; Klein, M. G.; Snell, G.; Lane, W.; Zou, H.; Levin, I.; Li, K.; Sang, B. C. Structure of Human GIVD Cytosolic Phospholipase A2 Reveals Insights into Substrate Recognition. *J. Mol. Biol.* **2016**, *428* (13), 2769–2779. https://doi.org/10.1016/j.jmb.2016.05.012.

(18) Tyson, G. H.; Halavaty, A. S.; Kim, H.; Geissler, B.; Agard, M.; Satchell, K. J.; Cho, W.; Anderson, W. F.; Hauser, A. R. A Novel Phosphatidylinositol 4,5-Bisphosphate Binding Domain Mediates Plasma Membrane Localization of ExoU and Other Patatin-like Phospholipases. *J. Biol. Chem.* **2015**, *290* (5), 2919–2937. https://doi.org/10.1074/jbc.M114.611251.
